# Stability of 20 Biogenic Amino Acids in Concentrated Sulfuric Acid: Implications for the Habitability of Venus' Clouds

**DOI:** 10.1089/ast.2023.0082

**Published:** 2024-04-10

**Authors:** Maxwell D. Seager, Sara Seager, William Bains, Janusz J. Petkowski

**Affiliations:** ^1^ Department of Chemistry and Biochemistry, Worcester Polytechnic Institute, Worcester, Massachusetts, USA.; ^2^ Nanoplanet Consulting, Concord, Massachusetts, USA.; ^3^ Department of Earth, Atmospheric and Planetary Sciences, Massachusetts Institute of Technology, Cambridge, Massachusetts, USA.; ^4^ Department of Physics, Massachusetts Institute of Technology, Cambridge, Massachusetts, USA.; ^5^ Department of Aeronautics and Astronautics, Massachusetts Institute of Technology, Cambridge, Massachusetts, USA.; ^6^ School of Physics & Astronomy, Cardiff University, Cardiff, United Kingdom.; ^7^ Rufus Scientific, Royston, United Kingdom.; ^8^ Faculty of Environmental Engineering, Wroclaw University of Science and Technology, Wroclaw, Poland.; ^9^ JJ Scientific, Warsaw, Poland.

**Keywords:** Venus, Habitability, NMR, Sulfuric acid, Amino acids

## Abstract

Scientists have long speculated about the potential habitability of Venus, not at the 700K surface, but in the cloud layers located at 48–60 km altitudes, where temperatures match those found on Earth's surface. However, the prevailing belief has been that Venus' clouds cannot support life due to the cloud chemical composition of concentrated sulfuric acid—a highly aggressive solvent. In this work, we study 20 biogenic amino acids at the range of Venus' cloud sulfuric acid concentrations (81% and 98% w/w, the rest water) and temperatures. We find 19 of the biogenic amino acids we tested are either unreactive (13 in 98% w/w and 12 in 81% w/w) or chemically modified in the side chain only, after 4 weeks. Our major finding, therefore, is that the amino acid backbone remains intact in concentrated sulfuric acid. These findings significantly broaden the range of biologically relevant molecules that could be components of a biochemistry based on a concentrated sulfuric acid solvent.

## 1. Introduction

The Venus clouds may be a potential habitat for life, despite the inhospitable conditions on the surface (see *e.g.,* Morowitz and Sagan, 1967; Schulze-Makuch and Irwin, 2006; Grinspoon and Bullock, 2007; Limaye *et al.*, 2018; Kotsyurbenko *et al*., 2021; Mogul *et al*., 2021a; Patel *et al*., 2021; Seager *et al*., 2021; Bains *et al*., 2023). The cloud layers on Venus are persistent and extend vertically from around 48–70 km above the surface (∼70 km in low latitudes and ∼65 km in polar latitudes), and they span the entire planet. Moreover, these cloud layers possess the essential prerequisites for supporting life (Baross *et al*., 2007), that is, suitable temperatures for covalent bonds, a liquid environment (comprising cloud droplets), and an energy source in the form of sunlight (see discussion in Seager *et al*., 2021; Bains *et al*., 2023). The notion that life could exist in a cloud environment is supported by analogy with Earth's aerial biosphere (Vaïtilingom *et al*., 2012; Amato *et al*., 2019) and our Venus life cycle concept, which explains how microbial-type life forms could potentially remain aloft indefinitely without descending to the lower, fatally hot atmospheric layers (Seager *et al*., 2021).

In contrast to this promising habitability viewpoint, the Venus clouds constitute a very hostile environment for Earth life. First, the clouds are predominantly composed of liquid concentrated sulfuric acid, a highly aggressive solvent that is thought to destroy most or all biochemicals (Bains *et al*., 2021c). Indeed, concentrated sulfuric acid is orders of magnitude more acidic than the most acidic environments on Earth that host acid-adapted microorganisms [*e.g.,* the hot, acidic, metal-rich Dallol pools in Ethiopia host polyextremophile organisms (Kotopoulou *et al*., 2018; Belilla *et al*., 2019; Gómez *et al*., 2019), but these pools are at pH ∼1, 10 orders of magnitude less acidic than concentrated sulfuric acid]. Second, there is almost no available water, and water is essential for all Earth-based life. Although the cloud droplets are composed of up to 30% water by volume (Hoffman *et al*., 1980; Krasnopolsky, 2015; Titov *et al*., 2018), the water is locked away in strong hydrogen bonds to sulfuric acid, rendering the water mostly unavailable for solvation or hydrogen bonding to other molecules. Further, the atmospheric conditions outside of the droplets appear to contain minimal water vapor, resulting in an extremely arid environment (Krasnopolsky, 2015). For further details on water availability and mitigating circumstances, see the works of Bains *et al.* (2021a; 2023), Hallsworth *et al.* (2021), Mogul *et al.* (2021a), and Rimmer *et al*. (2021).

We advocate the notion that the droplets of highly concentrated sulfuric acid might be able to support a form of life different from that of Earth. This is based on the idea that life could use sulfuric acid instead of water as a solvent—a concept supported by survival of complex organic molecules in highly concentrated sulfuric acid (Seager *et al*., 2023) and chemical reactions in highly concentrated sulfuric acid that generate a diverse set of organic molecules (Benner and Spacek, 2021; Spacek, 2021; Spacek and Benner, 2021). This perspective challenges the conventional planetary science view that only simple organic chemistry with limited functionality could be stable in concentrated sulfuric acid. The presence of a diverse organic chemistry in concentrated sulfuric acid has long been recognized outside the realm of planetary science. In the oil refining industry, concentrated sulfuric acid is employed to process crude oil and yields a byproduct known as “red oil,” which contains a wide array of organic compounds, including aromatic molecules that are stably dissolved in the concentrated sulfuric acid (Miron and Lee, 1963; Albright *et al*., 1972; Huang *et al*., 2015).

Spacek and Benner (Benner and Spacek, 2021; Spacek, 2021; Spacek and Benner, 2021) demonstrated that a rich organic chemistry spontaneously arises in concentrated sulfuric acid from simple precursor molecules, such as formaldehyde. Interestingly, even gas phase CO and CO_2_ can serve as seed molecules for this chemistry (Benner and Spacek, 2021; Spacek, 2021; Spacek and Benner, 2021), and these gases themselves could originate from photochemical processes in the atmosphere of Venus. Seager *et al*. (2023) conducted studies showing that nucleic acid bases, including adenine, cytosine, guanine, thymine, and uracil, as well as 2,6-diaminopurine and the “core” nucleic acid bases purine and pyrimidine, remain stable in sulfuric acid within the temperature and concentration range of the Venus clouds for at least 2 weeks. Complex organic chemistry is, of course, not life, but there is no life without it. The stability of complex organic chemistry in a specific environment can be considered a prerequisite to habitability. Thus, we aim at investigating the stability of biologically relevant organic molecules in concentrated sulfuric acid.

We are motivated to study amino acids because they are one of the key fundamental building blocks of life on Earth. While there are hundreds of known amino acids and thousands of more potential ones (Brown *et al.*, 2023), we focus on the 20 amino acids coded by Earth life's canonical genetic code, which we refer to as the 20 “biogenic” amino acids. Nine of the 20 biogenic amino acids have also been found in meteoritic material (Koga and Naraoka, 2017), which suggests a continuous supply to Venus. Our goal is to determine whether amino acids are, or are not, rapidly destroyed in concentrated sulfuric acid. We, therefore, use pure concentrated sulfuric acid for this foundational test, leaving the trace components in the Venus clouds and related chemistry for future work.

## 2. Materials and Methods

We purchased a set of L-amino acids from Millipore-Sigma, in the form of a set of biogenic L-amino acids with ≥98% purity (Cat. No. LAA21-1KT). Cysteine was in the form of cysteine hydrochloride and lysine in the form of lysine monohydrochloride. L-Methionine sulfoxide (Cat. No. M1126), L-Methionine sulfone (Cat. No. M0876), and *S*-sulfo-L-cysteine (Cat. No. C2196) all with purity ≥98% were purchased from Millipore-Sigma. L-Cysteine sulfinic acid (purity ≥98%; Cat. No. sc-203620), L-Cysteic acid (purity ≥98%; Cat. No. sc-485621), and *O*-sulfo-L-serine (purity >99%; Cat. No. sc-295959), were purchased from Santa Cruz Biotechnology, Inc. Iron (II) oxide (FeO) (purity ≥99.6%; Cat. No. 400866) and formic acid (purity ≥98%; Cat. No. 33015) were purchased from Sigma Aldrich. The compounds were used without further purification. We used D_2_SO_4_ from ACROS Organics (sulfuric acid-d_2_ for NMR, 98 wt.% in D_2_O, 99.5+ atom % D) and D_2_O (deuteration degree min 99.9%) from MagniSolv.

We prepared our NMR samples by dissolving 25–80 mg of the amino acids into 600 μL of solvent D_2_SO_4_ in D_2_O in glass vials. We used 10–40 mg of compounds for the 1D ^1^H and ^13^C NMR. We used 50–80 mg for some of the 2D NMR. When required, we heated sealed glass vials in a hot water bath (∼80°C for a few minutes) to promote dissolution of the compounds. We transferred the solution to 5 mm NMR tubes and stored the tubes for 12–18 h before NMR measurements. After NMR measurements, we stored the solutions in the NMR tubes, where the storage room temperature varied from about 18–24°C. To acquire NMR data, we used a Bruker Avance III-HD 400 MHz spectrometer equipped with a Prodigy liquid nitrogen cryoprobe (BBO) at 25°C. We acquired 1D ^1^H, ^13^C, and 2D ^1^H-^13^C HMQC NMR spectra to confirm the structures and, hence, stability of the compounds in 98% w/w and 81% w/w D_2_SO_4_ in D_2_O. In all cases, we locked on D_2_SO_4_. The D_2_SO_4_ peak is at 11.46 ± 0.02 ppm in 98% w/w D_2_SO_4_ and at 11.99 ± 0.02 ppm in 81% w/w D_2_SO_4_.

We used MNova software (Mestrelab Research) to process and analyze the NMR data (Willcott, 2009). The original data for all NMR experiments are available for download as Supplementary Datasets from Zenodo at https://zenodo.org/record/8381013. Institutional Review Board (IRB) Statement: Not applicable.

## 3. Results: Stability and Reactivity of Biogenic Amino Acids in Concentrated Sulfuric Acid

Our major finding is that the backbone of the amino acid molecule remains intact in concentrated sulfuric acid. We studied the 20 biogenic amino acids in concentrated sulfuric acid at the concentration range found in Venus' clouds (81% w/w and 98% w/w, the rest water) at room temperature. We found that 19 of the biogenic amino acids are either unreactive or chemically modified in the side chain only after 4 weeks in 98% w/w or 81% w/w concentrated sulfuric acid at room temperature and pressure. We note that for all the biogenic amino acids, the amino, and carboxyl groups are fully protonated, as they are in water with acidic pH (see Supplementary Data).

**FIG. 1.**
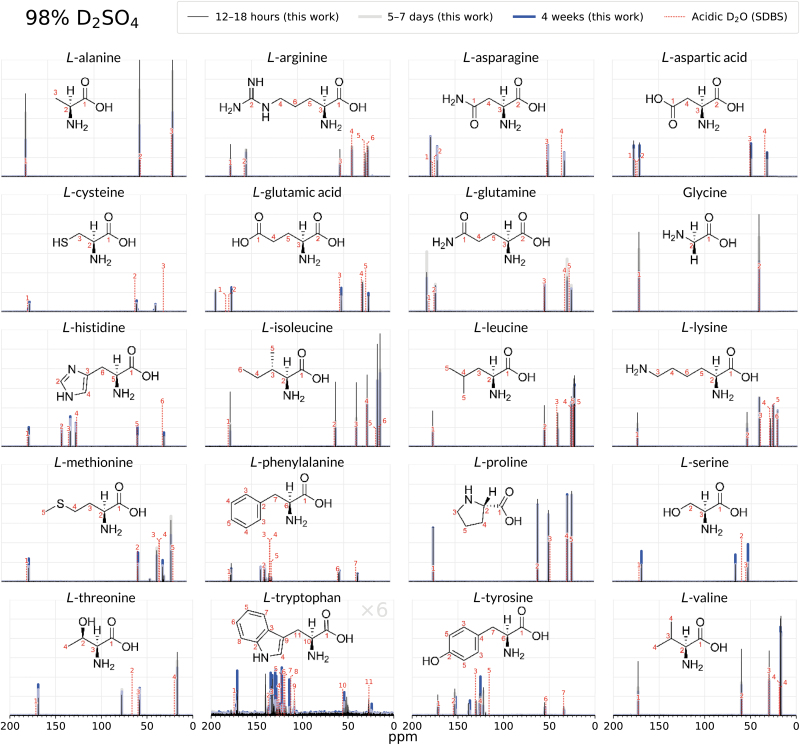
Comparison of the 1D ^13^C NMR spectra of 20 biogenic amino acids after 12–18 h (black), 5–8 days (grey), and after 4 weeks (blue) of incubation in 98% D_2_SO_4_/2% D_2_O (by weight). Thirteen amino acids (arginine, histidine, lysine, aspartic acid, glutamic acid, asparagine, glutamine, glycine, proline, alanine, isoleucine, leucine, valine) remain stable and unchanged for 4 weeks in 98% w/w D_2_SO_4_, as shown by the virtually identical peaks in the 4-week spectra and the 12–18 h spectra. A comparison with NMR peaks for each compound in acidic D_2_O (dashed lines; Li et al., 2020; Saito et al., 2006) further confirms the structural integrity of the amino acids and additionally shows that the overall molecular structure is not affected by the concentrated sulfuric acid solvent. The side chains of amino acids methionine, cysteine, serine, threonine, phenylalanine, tyrosine, and tryptophan undergo rapid chemical modification, as shown by emergence of additional NMR spectral peaks. For this set, with the exception of tryptophan, the amino acid backbone is intact, as illustrated by the unchanged NMR spectral peaks belonging to the α-carbon and the carboxylic group. Note that tryptophan intensities in sulfuric acid are multiplied by 6 so the peaks are visible at the scale of the figure. SDBS is the Spectral Database for Organic Compounds (Saito et al., 2006). See the Supplementary Data for more detailed NMR figures for each molecule.

**FIG. 2.**
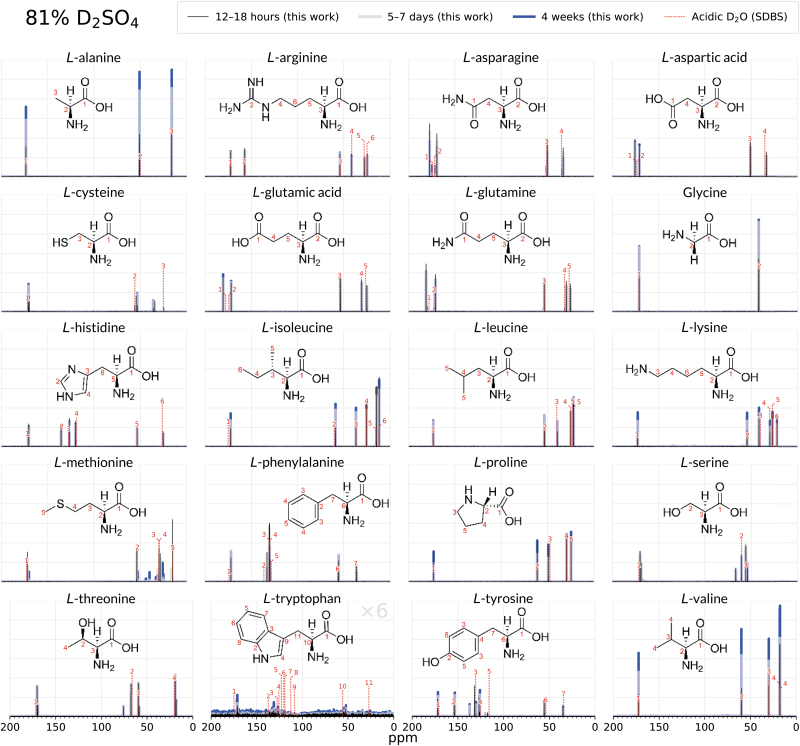
Comparison of the 1D ^13^C NMR spectra of 20 biogenic amino acids after 12–18 h (black), 5–8 days (grey), and after 4 weeks (blue) of incubation in 81% D_2_SO_4_/19% D_2_O (by weight). Twelve amino acids (arginine, histidine, lysine, aspartic acid, glutamic acid, glycine, proline, alanine, isoleucine, leucine, valine, phenylalanine) remain stable and unchanged for 4 weeks in 81% w/w D_2_SO_4_, as shown by the virtually identical peaks in the 4-week spectra and the 12–18 h spectra. A comparison with NMR peaks for each compound in acidic D_2_O (dashed lines; Li et al., 2020; Saito et al., 2006) further confirms the structural integrity of the amino acids and additionally shows that the overall molecular structure is not affected by the concentrated sulfuric acid solvent. The amino acids methionine, cysteine, serine, threonine, asparagine, glutamine, tyrosine, and tryptophan side chains undergo rapid chemical modification, as shown by emergence of additional NMR spectral peaks. For this set, with the exception of tryptophan, the amino acid backbone is intact, as illustrated by the unchanged NMR spectral peaks belonging to the α-carbon and carboxylic group. Note that tryptophan intensities in sulfuric acid are multiplied by 6 so the peaks are visible at the scale of the figure. SDBS is the Spectral Database for Organic Compounds (Saito et al., 2006). See the Supplementary Data for more detailed NMR figures for each molecule.

**FIG. 3.**
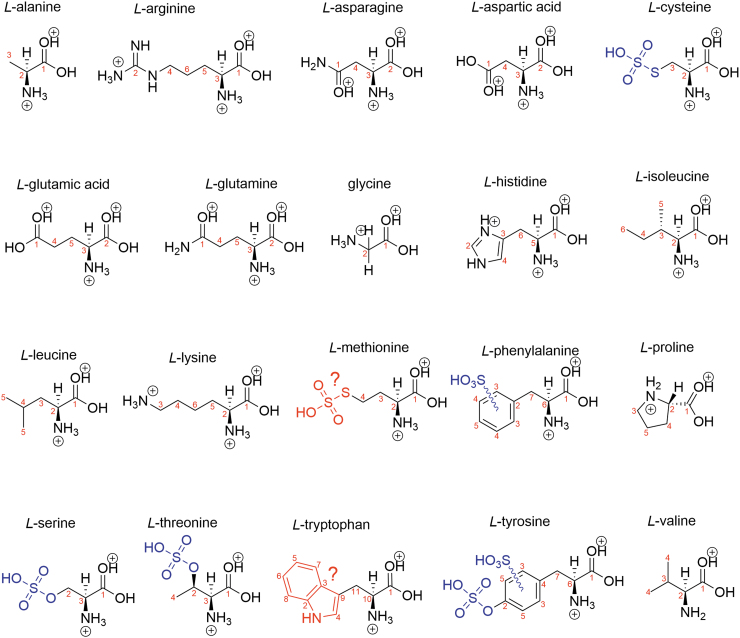
Biogenic amino acid structure in 98% w/w concentrated sulfuric acid with the rest water. Blue shows modifications, and red shows suspected modifications. Black is the structure of a protonated amino acid.

**FIG. 4.**
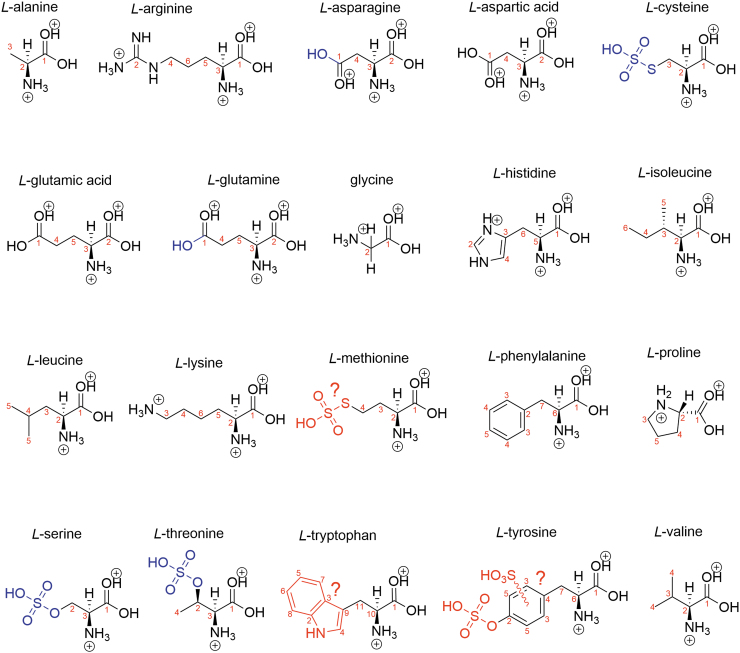
Biogenic amino acid structure in 81% w/w concentrated sulfuric acid with the rest water. Blue shows modifications, and red shows suspected modifications. Black is the structure of a protonated amino acid.

We found that 11 of the biogenic amino acids are stable in both 81% w/w and 98% w/w sulfuric acid. Nine are reactive, eight of which are stable to further change after rapid formation of side-chain derivatives in concentrated sulfuric acid. We now describe details in the text, Table 1 and Figs. 1–4. Out of the nine amino acids that are reactive in concentrated sulfuric acid, two are chemically modified in a similar way in concentrated sulfuric acid as they are in water (asparagine and glutamine). In 81% w/w sulfuric acid, both asparagine and glutamine are slowly converted to aspartic acid (in 1 week) and glutamic acid (in 4 weeks), a hydrolytic reaction that also happens more slowly in water (Supplementary Fig. S27). In 98% w/w concentrated sulfuric acid, asparagine and glutamine are unchanged (*i.e.,* not converted into aspartic acid and glutamic acid), presumably due to the much lower water activity. Four of the chemically modified amino acids are sulfated (*i.e.,* addition of an SO_3_H group to an oxygen or sulfur atom) in concentrated sulfuric acid, and two are sulfonated (addition of an SO_3_H group to a carbon atom), both of which are chemical modifications that cannot happen in pure water.

**Table 1.** Summary of the Reactivity of Amino Acids in Concentrated Sulfuric Acid at Room Temperature After 4 Weeks Based on the ^13^C NMR

Amino acid	Reactivity of amino acids in 98% w/w D_2_SO_4_	Reactivity of amino acids in 81% w/w D_2_SO_4_	Reactivity of amino acids in aqueous (H_2_O) solutions
Alanine	NR	NR	NR
Arginine	NR	NR	NR
Asparagine	NR	Deamidation	Slow deamidation (Pace et al., 2013)
Aspartic acid	NR	NR	NR
Cysteine	*S*-sulfation of thiol group (Reitz et al., 1946)	*S*-sulfation of thiol group (Reitz et al., 1946)	Sulfur oxidation (Garrido Ruiz et al., 2022)
Glutamine	NR	Deamidation	Very slow deamidation (Riggs et al., 2019)
Glutamic acid	NR	NR	NR
Glycine	NR	NR	NR
Histidine	NR	NR	NR
Isoleucine	NR	NR	NR
Leucine	NR	NR	NR
Lysine	NR	NR	NR
Methionine	Demethylation and *S*-sulfation (Andrews and Bruce, 1951)	Demethylation and *S*-sulfation (Andrews and Bruce, 1951)	Sulfur oxidation (Kim et al., 2014)
Phenylalanine	Sulfonation of phenyl ring (Habeeb, 1961; Reitz et al., 1946)	NR	NR
Proline	NR	NR	NR
Serine	*O*-sulfation of hydroxyl group (Reitz et al., 1946)	*O*-sulfation of hydroxyl group (Reitz et al., 1946)	NR
Threonine	*O*-sulfation of hydroxyl group (Reitz et al., 1946)	*O*-sulfation of hydroxyl group (Reitz et al., 1946)	NR
Tryptophan	Diverse cross-linked species formation (*e.g.,* Ramachandran and McConnell, 1955)	Diverse cross-linked species formation (*e.g.,* Ramachandran and McConnell, 1955)	Oxidation in the presence of ROS (Bellmaine et al., 2020; Simat and Steinhart, 1998)
Tyrosine	*O*-sulfation of hydroxyl group and/or sulfonation of phenyl ring (Habeeb, 1961; Reitz et al., 1946)	*O*-sulfation of hydroxyl group and/or oxidation of phenyl ring (Habeeb, 1961; Reitz et al., 1946)	Oxidation of the phenyl ring (Burzio and Waite, 2002; Recky et al., 2021)
Valine	NR	NR	NR

NR = no reaction; ROS = reactive oxygen species.

Serine and threonine are sulfated at the hydroxyl alcohol functional group (Supplementary Fig. S32), which is consistent with previously reported NMR spectra of sulfoserine and sulfothreonine (Rose *et al*., 1994). The sulfation of serine and threonine is less efficient in 81% w/w than in 98% w/w sulfuric acid. This conclusion is supported by the two populations of ^13^C carbon peaks in the NMR spectra in 81% w/w concentration that correspond to the two populations of serine and threonine—sulfated and native. This is an expected result, as sulfation of a hydroxyl is a dehydration reaction, and 98% w/w concentrated sulfuric acid is a more powerful dehydrating agent than 81% w/w concentrated sulfuric acid. We note that serine and threonine are deliberately sulfated by some life on Earth as a post-translational modification of proteins to modify biological functions of target proteins (Medzihradszky *et al*., 2004).

In cysteine, the thiol functional group is sulfated in concentrated sulfuric acid and yields *S*-sulfocysteine (Supplementary Fig. S28). This conclusion is supported by the comparison of 4-week incubated cysteine to the ^1^H and ^13^C NMR spectra of native *S*-sulfocysteine in concentrated sulfuric acid (Supplementary Fig. S28). Methionine is likely demethylated to homocysteine and subsequently sulfated to yield *S*-sulfohomocysteine in concentrated sulfuric acid (Supplementary Fig. S31). Such a demethylation reaction of methionine has previously been proposed to occur in concentrated sulfuric acid (Andrews and Bruce, 1951).

We now turn to the final three amino acids that are reactive in concentrated sulfuric acid. Phenylalanine appears to be sulfonated at multiple locations in the ring in 98% w/w sulfuric acid within 1 day, as shown by the additional carbon peaks in the “aromatic region” of the ^13^C NMR spectra. In contrast, in 81% w/w sulfuric acid, the phenylalanine structure remains unchanged for at least 4 weeks. The modification of the phenylalanine aromatic ring in 98% w/w is likely not an oxidation, as oxidation of aromatics is not known to happen in concentrated sulfuric acid (Liler, 1971). Tyrosine appears to undergo several chemical changes over time in 98% w/w concentrated sulfuric acid, likely due to sulfation of the hydroxyl alcohol functional group (Liler, 1971) and/or sulfonation of the phenyl ring, producing multiple populations of modified molecules, as illustrated by the appearance of a multitude of additional peaks on the ^13^C NMR spectrum. The early studies on the chemical reactivity of proteins and amino acids in 98% w/w sulfuric acid also support the sulfation and sulfonation of the phenyl ring of tyrosine (Reitz *et al*., 1946; Habeeb, 1961). Tyrosine in 81% w/w concentrated sulfuric acid likely becomes sulfated at the hydroxyl alcohol functional group and possibly sulfonated as well. We note that tyrosine in water is oxidized (*e.g.,* Burzio and Waite, 2002; Recky *et al*., 2021); whether such oxidation in concentrated sulfuric acid happens remains to be ascertained.

Only one of the chemically modified amino acids is highly reactive in concentrated sulfuric acid. Tryptophan undergoes rapid and complex reactivity that likely involves the formation of highly diverse cross-linked species^*^ as, for example, noted in passing by Ramachandran and McConnell (1955). We note that in water tryptophan can readily be oxidized by reactive oxygen species such as singlet oxygen that can be generated from atmospheric oxygen by UV light or reaction with metals, such as iron, to react with a variety of species (Simat and Steinhart, 1998; Bellmaine *et al*., 2020).

The remaining 11 biogenic amino acids (alanine, arginine, aspartic acid, glutamic acid, glycine, histidine, isoleucine, leucine, lysine, proline, and valine) show no detectable reactivity and degradation of the tested compound after incubation in concentrated sulfuric acid for at least 4 weeks in both 98% w/w and 81% w/w concentrated sulfuric acid at room temperature. These 11 biogenic amino acids are also stable in water. We demonstrate their stability by comparing their ^13^C NMR spectral peak shifts in concentrated sulfuric acid with published literature values obtained in acidic water (see Supplementary Tables S1–S20) (Saito et al., 2006) and show they are the same and do not change with time (Figs. 1 and 2). We further confirm the amino acid structures via ^1^H NMR and ^1^H-^13^C 2D HMQC NMR (see Supplementary Figs. S1–S4).

## 4. Discussion

Our major finding is that the backbone structure of 19 of the 20 biogenic amino acids is unperturbed in concentrated sulfuric acid (the exception being tryptophan). Eleven are stable in concentrated acid; the other eight are modified only in the side chain. Concentrated sulfuric acid's chemical properties differ significantly from that of water, so much so that our results may appear unexpected. Our findings help to challenge the prevailing misconception in the astrobiology and biology communities that organic chemicals are uniformly unstable in concentrated sulfuric acid.^†^ In fact, experiments demonstrating stability of organic compounds in concentrated sulfuric acid research date back to the 1920s, though unconnected to Venus' clouds. Examples of such early research include stability and reactivity studies of proteins (Bischoff and Sahyun, 1929; Reitz *et al*., 1946; Habeeb, 1961), lipids (Steigman and Shane, 1965), and various other organics, for example, Albright *et al*. (1972) and Miron and Lee (1963). See also the work of Bains *et al*. (2021b, 2021c) for a recent review of reactivity and stability of organic chemicals in concentrated sulfuric acid.

The Venus clouds are expected to have trace components and be different from pure “test tube” conditions. Extensive *in situ* and Earth-ground based observations over the past several decades show a variety of gases, metals, and other compounds detected in the cloud layers and cloud particles (see the recent review article of Petkowski *et al*., 2023 and the original work of Petrianov *et al*., 1981; Andreichikov, 1987a, 1987b; Mogul *et al.*, 2021b; Zolotov *et al*., 2023).

Before addressing reactivity in complex mixtures, we need preliminary fundamental experiments, hence this work's focus on pure concentrated sulfuric acid. Some of the trace species in the Venusian clouds, however, may react with amino acids. If there is reactivity, we want to acknowledge that, for life to function, there is a crucial balance between biochemical's reactivity and stability. Life relies on chemicals that are mostly on the verge of spontaneous chemical reactivity, so that minimal catalysis can allow the reactions of metabolism. Extremely stable compounds, such as fluorocarbon “forever chemicals,” tend to be toxic because biochemistry cannot process them (Brunn *et al*., 2023). Testing with trace species is a topic to be expanded upon in future work. To initiate such studies, we tested the stability of two key amino acids, alanine and glycine, in the presence of two key contaminants, CO (released from formic acid) and FeO, at room temperature, and found that the amino acids are stable under these conditions as well as in pure concentrated sulfuric acid (tested for up to 1 week) (see Supplementary Data: Section S5 and Supplementary Figs. S33–S35 for details).

Regarding the modification of the amino acid side chains seen in our experiments, our own life routinely modifies side chains of amino acids. Out of the 20 amino acids in our protein-building repertoire, only the side chains of small hydrophobic amino acids alanine, valine, leucine, and isoleucine are not modified by life. The rest are often modified (a phenomenon called post-translational modification of amino acids). Further, often one amino acid can be modified in many different ways (*e.g.,* lysine can be either methylated or acetylated or even phosphorylated). Such amino acid modifications give life an ability to modify protein function, including the ability to give existing proteins entirely new functions in the cell.

There are a few key points relating our amino acid studies in concentrated sulfuric acid to Venus' cloud habitability. There is the question of which amino acids any hypothetical Venus life might use. We chose to test the stability and reactivity of the 20 biogenic amino acids used by life on Earth as they are a well-defined set of compounds that our life uses. Nine of the biogenic amino acids have also been identified in meteoritic material (Koga and Naraoka, 2017); seven of these fall into the category of unreactive amino acids; and two are sulfated (serine and threonine) and then are stable to further reactivity in concentrated sulfuric acid. Therefore, presumably a small but steady supply of some amino acids could be delivered via meteoritic infall to the Venus clouds. There are 500 known amino acids and thousands of more possibilities (Brown *et al*., 2023). Our work shows the backbone of the amino acid is stable in concentrated sulfuric acid, pointing toward a large variety of amino acids to be stable in concentrated sulfuric acid.

Our work also informs the possible origins of life on Venus, if life exists there. In one scenario, life may have originated in water oceans hypothesized to have existed on Venus' surface for up to billions of years, before evaporating (Way *et al.*, 2016; Way and Del Genio, 2020). As water became very scarce, life would have adapted its biochemistry from water to a concentrated sulfuric acid environment. In an opposing scenario, Venus may always have been too warm to host water oceans (Turbet *et al*., 2021) and may have had concentrated sulfuric acid in its clouds as a dominant liquid for most of its geological history, in which case prebiotic chemistry would have to generate life in concentrated sulfuric acid. This second scenario might be thought implausible, if the traditional view of concentrated sulfuric acid as inimical to complex chemistry is held. Our work shows that either scenario could be plausible routes to the origin of life, as we show that 11 of our 20 amino acids are unmodified in both 98% w/w and 81% w/w sulfuric acid, so that a set of unreactive amino acids to utilize is readily available in concentrated sulfuric acid no matter how life ends up there.

Our goal was to check whether amino acids are or are not rapidly destroyed in concentrated sulfuric acid. Modifications that do occur to amino acid side chains in concentrated sulfuric acid generally happen quickly, within hours to days. We stopped monitoring the reactivity of amino acids in concentrated sulfuric acid after 4 weeks of incubation at room temperature, as our experiments showed no further change in stability. (The one exception was tryptophan, which degraded completely in concentrated sulfuric acid; and after 4 weeks, new dominant chemical species emerged from the reaction's unknown products.) We note that not all terrestrial biochemicals are stable in water. For example, asparagine spontaneously converts to aspartic acid in proteins on a timescale of months even at neutral pH (Yang and Zubarev, 2010). Other amino acids, depending on the reaction conditions, may oxidize on a timescale of hours to days (cysteine, methionine, tryptophan, and tyrosine) (Grassi and Cabrele, 2019). Longer term stability studies of amino acids in concentrated sulfuric acid may be of interest for future work.

A final key point regarding the connection between our results and Venus' habitability has to do with further questions that include racemization of amino acid chiral centers (*e.g.,* Bada, 1985), the possibility of utilization of amino acids (or similar building blocks) in large polymeric structures, and, as mentioned, amino acid stability in non-pure sulfuric acid droplets as the Venus cloud particles likely are. These are also questions for future work.

Concentrated sulfuric acid's properties differ vastly from aqueous or diluted acid solutions, which challenge typical organic and biochemistry assumptions. Studying organic chemistry in pure sulfuric acid requires a direct, unbiased approach, without prior preconceptions and assumptions, as highlighted by Spacek and Benner's works (Benner and Spacek, 2021; Spacek, 2021; Spacek and Benner, 2021). Our work furthers this concept by presenting unexpected findings regarding the reactivity and stability of amino acids—one of potential life's building blocks in this unique solvent.

Venus, our neighboring planet, lies conveniently close, which allows us to directly probe its cloud particles through space missions. In the 1970s and 1980s NASA's Pioneer Venus *in situ* missions and several Soviet descent craft and landers studied the clouds but did not unambiguously determine the composition of all types of cloud particles or search for the presence of organic chemicals. Presently, NASA and ESA have plans to dispatch missions to Venus by the end of this decade (de Oliveira *et al*., 2018; Garvin *et al*., 2022; Smrekar *et al*., 2022), and Rocket Lab plans to launch a small mission in 2025 (French *et al*., 2022). Ultimately, a sample return from the Venusian atmosphere may be necessary to robustly ascertain the presence of life, if indeed life exists there (Schulze-Makuch and Irwin, 2002; Shibata *et al.*, 2017; Seager *et al*., 2022; Limaye and Garvin, 2023).

We are at the dawn of a new branch of Astrobiology and a new branch of organic chemistry. We close with a call to action to study organic chemistry in alternative solvents from water, which is crucial for the true understanding of the extent of the habitability of the Galaxy.

## Supplementary Material

Supplemental data

## Data Availability

Original data are deposited in Zenodo data repository at https://zenodo.org/record/8381013.

## Abbreviations Used

NR: no reaction
ROS: reactive oxygen species

## Notes

^*^
We note that the O_2_ from the atmosphere could contribute to the destruction of tryptophan in concentrated sulfuric acid.

^†^
Perhaps such misconceptions stem from the improper extrapolation of the results of a popular experiment of sugar's reactivity with concentrated sulfuric acid (Pines *et al*., 2012).

## Notes

Associate Editor: Sherry L. Cady
