# Supplementary material for: Stability of 20 Biogenic Amino Acids in Concentrated Sulfuric Acid: Implications for the Habitability of Venus' Clouds
Source: Astrobiology. 2024 Apr 10;24(4):386–96. doi: 10.1089/ast.2023.0082 (PMC11035925; doi:10.1089/ast.2023.0082)
Supplement: Supplemental data [file Suppl_Data.docx]

**Supplementary Information

Stability of Biogenic Amino Acids in Concentrated Sulfuric Acid: Implications for the Habitability of Venus’ Clouds**

Maxwell D. Seager^1,2,#^, Sara Seager ^3,4,5,#,*^, William Bains ^3,6,7^ and Janusz J. Petkowski ^3,8,9#^

^1^ Nanoplanet Consulting, Concord, MA 01742, USA

^2^ Department of Chemistry and Biochemistry, Worcester Polytechnic Institute, Worcester, MA 01609, USA

^3^ Department of Earth, Atmospheric and Planetary Sciences, Massachusetts Institute of Technology, 77 Massachusetts. Avenue., Cambridge, MA 02139, USA

^4^ Department of Physics, Massachusetts Institute of Technology, 77 Massachusetts. Avenue., Cambridge, MA 02139, USA

^5^ Department of Aeronautics and Astronautics, Massachusetts Institute of Technology, 77 Massachusetts. Avenue., Cambridge, MA 02139, USA

^6^ School of Physics & Astronomy, Cardiff University, 4 The Parade, Cardiff CF24 3AA, UK

^7^ Rufus Scientific, Melbourn, Herts SG8 6ED, UK

^8^ Faculty of Environmental Engineering, Wroclaw University of Science and Technology, 50-370 Wroclaw, Poland

^9^ JJ Scientific, Mazowieckie, Warsaw 02-792, Poland

******* Correspondence: [seager@mit.edu](mailto:seager@mit.edu)

# contributed equally to this work

Supplementary Information Text

In this Supplementary Information file, we present experimental data beyond the ^13^C NMR presented in the main text. The goal is to provide detailed experimental results to further support the conclusions of results described in the main paper. We support results from the ^13^C NMR experiments (Figure S7-S16) with ^1^H NMR (Figure S17-S26) and 2D NMR (Figure S1-S4) experiments to further demonstrate the long term, four week, integrity of the amino acids’ structures in the sulfuric acid concentrations (81% w/w and 98% w/w, the rest water) found in Venus’ clouds.

Specifically, we provide 1D ^1^H NMR and 2D ^1^H-^13^C Heteronuclear Multiple-Quantum Correlation spectroscopy (HMQC) that shows which hydrogen and carbon atoms in the analyzed molecule are directly bonded to each other in solution. We begin with a more detailed description of chemical stability or modification of each of the 20 biogenic amino acids.

To simplify the description of the ^13^C peak assignments we use the term "around" to indicate the chemical shift positions of individual spectral peaks, or their split variants. Over the span of days to weeks, the peaks on the ^13^C NMR spectra of amino acids incubated in 98% w/w and 81% w/w sulfuric acid tend to undergo splitting. Such splitting is likely due to H/D exchange between the solvent and the dissolved chemical or possibly due to other solvent effects and is not an indication of the overall instability of the entire amino acid structure.

**S1. Assessment of Chemical Stability and Reactivity of Biogenic Amino Acids in 98% (w/w) Concentrated Sulfuric Acid.**

Below we summarize carbon peak assignments and the overall results of the ^13^C NMR experiments for each of the 20 biogenic amino acids in the 98% w/w sulfuric acid at room temperature.

Alanine has 3 carbon atoms in its structure. The 3 carbons match the 3 carbon peaks visible in the ^13^C NMR spectrum (Figure S7A). The downfield peak at 173.96 ppm corresponds to the carboxyl group carbon. We identify the alpha carbon at around 51 ppm which matches the alpha carbon peak in D_2_O. Other carbon spectral peaks also are consistent with the carbon peaks recorded in D_2_O (Table S1). The amino acid is stable for at least four weeks in 98% w/w sulfuric acid (Figure S7A).

Arginine has 6 carbon atoms in its structure. The 6 carbons match the 6 carbon peaks visible in the ^13^C NMR spectrum (Figure S7B). The most downfield shifted peak around 172.7 ppm corresponds to the carboxyl group carbon. The second downfield peak at 155.79 ppm corresponds to the carbon of the guanidine group of the arginine side chain. We can identify the alpha carbon at around 54.4 ppm which matches the alpha carbon peak in D_2_O. Other carbon spectral peaks also are consistent with the carbon peaks recorded in D_2_O (Table S2). The amino acid is stable for at least four weeks in 98% w/w sulfuric acid (Figure S7B).

Asparagine has 4 carbon atoms in its structure. The 4 carbons match the 4 carbon peaks visible in the ^13^C NMR spectrum (Figure S7C). The most downfield shifted peak at 176.53 ppm corresponds to the carbon of the side chain amide group. The second downfield peak around 169.3 ppm corresponds to the carboxyl group carbon. We can identify the alpha carbon at around 49 ppm which matches the alpha carbon peak in D_2_O. Other carbon spectral peaks also are consistent with the carbon peaks recorded in D_2_O (Table S3). The amino acid is stable for at least four weeks in 98% w/w sulfuric acid and does not undergo deamidation reaction, in contrast to 81% w/w (Figure S27).

Aspartic acid has 4 carbon atoms in its structure. The 4 carbons match the 4 carbon peaks visible in the ^13^C NMR spectrum (Figure S7D). The most downfield shifted peak at 176.86 ppm corresponds to the carbon of the side chain carboxyl group. The second downfield peak around 170.7 ppm corresponds to the carboxyl group carbon. We can identify the alpha carbon at around 50 ppm which matches the alpha carbon peak in D_2_O. Other carbon spectral peaks also are consistent with the carbon peaks recorded in D_2_O (Table S4). The amino acid is stable for at least four weeks in 98% w/w sulfuric acid (Figure S7D).

Cysteine has 3 carbon atoms in its structure. The 3 carbons match the 3 carbon peaks visible in the ^13^C NMR spectrum (Figure S8A). The downfield peak around 169.8 ppm corresponds to the carboxyl group carbon. We can identify the alpha carbon at around 54 ppm which matches the alpha carbon peak in D_2_O. The carbon peak C3 adjacent to the cysteine thiol group is significantly shifted downfield as compared to C3 peak in D_2_O (Table S5). The downfield shift is consistent with the deshielding of the C3 carbon due to the sulfation of the thiol group in 98% w/w sulfuric acid to *S*-sulfo-cysteine (Figure S28). To confirm that cysteine is indeed *S*-sulfated we have compared the ^13^C and ^1^H NMR spectra of cysteine incubated in 98% w/w D_2_SO_4_ for 12-18 h and four weeks to the ^13^C and ^1^H NMR spectra of native *S*-sulfocysteine collected in 98% w/w D_2_SO_4_. Both ^13^C and ^1^H NMR spectra are virtually identical confirming the sulfation of cysteine in concentrated sulfuric acid (Figure S28). This conclusion is also supported by early literature reports on the modifications of amino acids, including sulfation of cysteine, in concentrated sulfuric acid (Reitz et al., 1946). The *S*-sulfation of the thiol group of cysteine proceeds quite readily and after 12-18h cysteine is completely *S*-sulfated (Figure S28)^[[1]](#footnote-1)^. The sulfation of the side chain thiol group does not lead to the instability and reactivity of the entire amino acid structure and can be classified as a side-chain modification. For completeness we have also ruled out the oxidation of cysteine to sulfinic and sulfonic acid in 98% w/w sulfuric acid (Figure S29). The ^13^C and ^1^H NMR spectra of sulfinic and sulfonic acid collected in 98% w/w sulfuric acid do not match the spectra of cysteine incubated in 98% w/w sulfuric acid, ruling out the direct oxidation of cysteine as a possible side chain modification in concentrated sulfuric acid (Figure S29). The published ^13^C and ^1^H NMR spectra of cysteine sulfinic (Hu et al., 2014) and cysteine sulfonic acid (Stanulis and Barron, 2022; Weierbach et al., 2023) collected in D_2_O also do not match our cysteine spectra, further ruling out direct oxidation as a possible side chain modification of cysteine in concentrated sulfuric acid. Note that the oxidation of cysteine to sulfenic, sulfinic, and sulfonic acid does happen in water, both in vitro as well as in vivo (see e.g. (Garrido Ruiz et al., 2022)).

Glutamine has 5 carbon atoms in its structure. The 5 carbons match the 5 carbon peaks visible in the ^13^C NMR spectrum (Figure S8B). The most downfield shifted peak at 180.53 ppm corresponds to the carbon of the side chain amide group. The second downfield peak around 171 ppm corresponds to the carboxyl group carbon. We can identify the alpha carbon at around 53 ppm which matches the alpha carbon peak in D_2_O. Other carbon spectral peaks also are consistent with the carbon peaks recorded in D_2_O (Table S6). The amino acid is stable for at least four weeks in 98% w/w sulfuric acid and does not undergo deamidation reaction, in contrast to 81% w/w (Figure S27).

Glutamic acid has 5 carbon atoms in its structure. The 5 carbons match the 5 carbon peaks visible in the ^13^C NMR spectrum (Figure S8C). The most downfield shifted peak at around 189 ppm corresponds to the carbon of the side chain carboxyl group. The second downfield peak around 171.5 ppm corresponds to the carboxyl group carbon. We can identify the alpha carbon at around 53 ppm which matches the alpha carbon peak in D_2_O. Other carbon spectral peaks also are consistent with the carbon peaks recorded in D_2_O (Table S7). The amino acid is stable for at least four weeks in 98% w/w sulfuric acid (Figure S8C).

Glycine has 2 carbon atoms in its structure. The 2 carbons match the 2 carbon peaks visible in the ^13^C NMR spectrum (Figure S8D). The downfield peak around 171 ppm corresponds to the carboxyl group carbon. The second peak at around 41.6 ppm also matches the corresponding carbon peak in D_2_O (Table S8). The amino acid is stable for at least four weeks in 98% w/w sulfuric acid (Figure S8D).

Histidine has 6 carbon atoms in its structure. The 6 carbons match the 6 carbon peaks visible in the ^13^C NMR spectrum (Figure S9A). The downfield peak around 170.5 ppm corresponds to the carboxyl group carbon. The other 3 downfield peaks, C2, C3, C4, in the “aromatic region” of the spectrum correspond to the 3 carbons of the imidazole ring. We can identify the alpha carbon at around 53 ppm which matches the alpha carbon peak in D_2_O. Other carbon spectral peaks also are consistent with the carbon peaks recorded in D_2_O (Table S9). The amino acid is stable for at least four weeks in 98% w/w sulfuric acid (Figure S9A).

Isoleucine has 6 carbon atoms in its structure. The 6 carbons match the 6 carbon peaks visible in the ^13^C NMR spectrum (Figure S9B). The downfield peak at 172.95 ppm corresponds to the carboxyl group carbon. We can identify the alpha carbon at around 59 ppm which matches the alpha carbon peak in D_2_O. Other carbon spectral peaks also are consistent with the carbon peaks recorded in D_2_O (Table S10). The amino acid is stable for at least four weeks in 98% w/w sulfuric acid (Figure S9B).

Leucine has 6 carbon atoms in its structure. The 6 carbons match the 6 carbon peaks visible in the ^13^C NMR spectrum (Figure S9C). The downfield peak around 174 ppm corresponds to the carboxyl group carbon. The symmetry of the two carbons C5 in the leucine side chain is broken but it matches the peaks recorded in D_2_O. We can identify the alpha carbon at around 53.5 ppm which matches the alpha carbon peak in D_2_O. Other carbon spectral peaks also are consistent with the carbon peaks recorded in D_2_O (Table S11). The amino acid is stable for at least four weeks in 98% w/w sulfuric acid (Figure S9C).

Lysine has 6 carbon atoms in its structure. The 6 carbons match the 6 carbon peaks visible in the ^13^C NMR spectrum (Figure S9D). The downfield peak around 172.8 ppm corresponds to the carboxyl group carbon. We can identify the alpha carbon at around 54.5 ppm which matches the alpha carbon peak in D_2_O. Other carbon spectral peaks also are consistent with the carbon peaks recorded in D_2_O (Table S12). The amino acid is stable for at least four weeks in 98% w/w sulfuric acid (Figure S9D).

Methionine has 5 carbon atoms in its structure. The 5 carbons do not entirely match the number of carbon peaks visible in the ^13^C NMR spectrum (Figure S10A). The downfield peak around 170.5 ppm corresponds to the carboxyl group carbon. We can identify the alpha carbon at around 52.7 ppm which matches the alpha carbon peak in D_2_O (Table S13). Oxidation of the side chain sulfur atom is likely not the reason for the emergence of the additional peaks on the ^13^C and ^1^H NMR spectra. We have ruled out oxidation of methionine to methionine sulfoxide and methionine sulfone in 98% w/w sulfuric acid (Figure S30). The ^13^C and ^1^H NMR spectra of methionine sulfoxide and methionine sulfone collected in 98% w/w sulfuric acid do not match the NMR spectra of methionine incubated in 98% w/w sulfuric acid, ruling out the oxidation of methionine as a possible side chain modification in concentrated sulfuric acid (Figure S30). The published ^13^C and ^1^H NMR spectra of methionine sulfoxide (Aguiar et al., 2019; Emmanuel et al., 2017; Karunakaran-Datt and Kennepohl, 2009; Skvortsov et al., 2003; Xu et al., 2021) and methionine sulfone (Elsherbini et al., 2019; Emmanuel et al., 2017; Karunakaran-Datt and Kennepohl, 2009; Saito et al., 2006; Skvortsov et al., 2003) collected in D_2_O also do not match our methionine spectra, further ruling out oxidation as a possible side chain modification. We note however that in contrast to concentrated sulfuric acid the oxidation of methionine does happen in water, both in vitro and in vivo (see e.g. (Kim et al., 2014; Liang et al., 2012; Sen et al., 2017)).
We hypothesize that in concentrated sulfuric acid methionine undergoes demethylation to homocysteine. As a result of the demetylation of methionine’s side chain the emerging free thiol group of homocysteine can readily undergo *S*-sulfation, analogously to *S*-sulfation of cysteine thiol group (Figure S31). Methionine demethylation in concentrated sulfuric acid has been suggested before as one of the possible reactions of methionine in this solvent (Andrews and Bruce, 1951; Butz and du Vigneaud, 1932).

Phenylalanine has 9 carbon atoms in its structure. The 9 carbons do not match the carbon peaks visible in the ^13^C NMR spectrum (Figure S10B). The side chain aromatic ring of phenylalanine likely gets sulfonated resulting in many additional signals in the “aromatic” region of the spectrum. The additional peaks correspond to a variety of sulfonated products that complicate the reliable NMR assignment. The downfield peak around 172 ppm corresponds to the carboxyl group carbon. We can identify the alpha carbon at around 55 ppm which matches the alpha carbon peak in D_2_O (Table S14) and can also tentatively assign carbon C7 to peak around 35 ppm (Table S14). These assignments further confirm the stability of the amino acid backbone structure and support the conclusion that any reactivity of phenylalanine is confined to the side chain aromatic ring. With the data at hand we cannot reliably assign the carbon atoms of the aromatic ring.

Proline has 5 carbon atoms in its structure. The 5 carbons match the 5 carbon peaks visible in the ^13^C NMR spectrum (Figure S10C). The downfield peak around 173.7 ppm corresponds to the carboxyl group carbon. We can identify the alpha carbon at around 60.5 ppm which matches the alpha carbon peak in D_2_O. Other carbon spectral peaks also are consistent with the carbon peaks recorded in D_2_O (Table S15). The amino acid is stable for at least four weeks in 98% w/w sulfuric acid (Figure S10C).

Serine has 3 carbon atoms in its structure. The 3 carbons match the 3 carbon peaks visible in the ^13^C NMR spectrum (Figure S10D), although we note that the hydroxyl group of serine is readily sulfated in 98% w/w sulfuric acid (Figure S32). To confirm that serine is indeed sulfated we have compared the ^13^C and ^1^H NMR spectra of serine incubated in 98% w/w D_2_SO_4_ for four weeks to the ^13^C and ^1^H NMR spectra of native *O*-sulfoserine collected in 98% w/w D_2_SO_4_ and D_2_O. Both ^13^C and ^1^H NMR spectra are virtually identical confirming the sulfation of serine in concentrated sulfuric acid (Figure S32). Sulfation of serine also agrees with previous studies on sulfation of aliphatic alcohols in concentrated sulfuric acid (Iraci et al., 2002; Kane and Leu, 2001) and the early NMR studies on sulfoserine and sulfothreonine (Rose et al., 1994). The carbon peak C2 adjacent to the serine hydroxyl group is significantly shifted downfield as compared to C2 peak in D_2_O (Table S16). The downfield shift is consistent with the deshielding of the C2 carbon due to the sulfation of the OH group in 98% w/w sulfuric acid. Sulfation does not result in the instability of the entire amino acid structure, rather it can be classified as chemical modification of the amino acid side chain. The downfield peak around 168.6 ppm corresponds to the carboxyl group carbon. We can identify the alpha carbon at around 53.5 ppm which matches the alpha carbon peak in D_2_O (Table S16).

Threonine has 4 carbon atoms in its structure. The 4 carbons match the 4 carbon peaks visible in the ^13^C NMR spectrum (Figure S11A), although we note that the hydroxyl group of threonine is likely sulfated. Sulfation of threonine agrees with previous studies on sulfation of aliphatic alcohols in concentrated sulfuric acid (Iraci et al., 2002; Kane and Leu, 2001) and the early NMR studies on sulfoserine and sulfothreonine (Rose et al., 1994). The carbon peak C2 adjacent to the threonine hydroxyl group is significantly shifted downfield as compared to C2 peak in D_2_O (Table S17). The downfield shift is consistent with the deshielding of the C2 carbon due to the sulfation of the OH group in 98% w/w sulfuric acid. Sulfation does not result in the instability of the entire amino acid structure, rather it can be classified as chemical modification of the amino acid side chain. The downfield peak around 169 ppm corresponds to the carboxyl group carbon. We can identify the alpha carbon at around 58 ppm which matches the alpha carbon peak in D_2_O (Table S17).

Tryptophan has 11 carbon atoms in its structure. The ^13^C NMR spectrum shows that tryptophan is unstable 98% w/w sulfuric acid (Figure S11B). Upon dissolution in 98% w/w sulfuric acid tryptophan solution gained a bright yellow color. After 3 weeks the tryptophan solution turned into a dark red beetroot-like color, and subsequent change to dark green color a week later (i.e. after four weeks total). Tryptophan undergoes rapid and complex reactivity that likely involves the formation of highly diverse cross-linked species, as e.g. noted in passing by (Ramachandran and McConnell, 1955)). Our results are generally consistent with the early studies on the reactivity of tryptophan in 98% w/w sulfuric acid. For example the early work of Habeeb suggests a modification and a complete degradation of the indole ring of tryptophan in 98% w/w sulfuric acid (Habeeb, 1961).

Tyrosine has 9 carbon atoms in its structure, 7 of which are unique. The 7 unique carbons do not match the 9 carbon peaks visible in the ^13^C NMR spectrum (Figure S11C). The sulfation of the side chain OH group and sulfonation at the single site in the phenol ring of tyrosine is possible in 98% w/w sulfuric acid. Both sulfation and sulfonation of the aromatic ring could explain the 6 carbon peaks in the “aromatic region” of the spectrum. The early studies on the chemical reactivity of proteins and amino acids in 98% w/w sulfuric acid also support the sulfation and sulfonation of the phenyl ring of tyrosine (Habeeb, 1961; Reitz et al., 1946). The downfield peak around 172 ppm corresponds to the carboxyl group carbon. We can identify the alpha carbon at around 55 ppm which matches the alpha carbon peak in D_2_O (Table S19) and can also tentatively assign carbon C7 to peak around 34 ppm (Table S19). These assignments further confirm the stability of the amino acid backbone structure and support the conclusion that any reactivity of tyrosine is confined to the side chain aromatic ring. However due to the overall reactivity of tyrosine’s side chain in the 98% w/w sulfuric acid we cannot reliably assign the aromatic ring carbon peaks to their respective carbon atoms in the tyrosine structure.

Valine has 5 carbon atoms in its structure. The 5 carbons match the 5 carbon peaks visible in the ^13^C NMR spectrum (Figure S11D). The downfield peak around 172.98 ppm corresponds to the carboxyl group carbon. The symmetry of the two C4 carbons in the side chain is broken but it matches the peaks recorded in D_2_O. We can identify the alpha carbon at around 60 ppm which matches the alpha carbon peak in D_2_O. Other carbon spectral peaks also are consistent with the carbon peaks recorded in D_2_O (Table S20). The amino acid is stable for at least four weeks in 98% w/w sulfuric acid (Figure S11D).

**S2. Assessment of Chemical Stability and Reactivity of Biogenic Amino Acids in 81% (w/w) Concentrated Sulfuric Acid.**

Below we summarize carbon peak assignments and the overall results of the ^13^C NMR experiments for each of the 20 biogenic amino acids in the 81% w/w sulfuric acid at room temperature.

Alanine has 3 carbon atoms in its structure. The 3 carbons match the 3 carbon peaks visible in the ^13^C NMR spectrum (Figure S12A). The downfield peak at around 173.6 ppm corresponds to the carboxyl group carbon. We identify the alpha carbon at around 51 ppm which matches the alpha carbon peak in D_2_O. Other carbon spectral peaks also are consistent with the carbon peaks recorded in D_2_O (Table S1). The amino acid is stable for at least four weeks in 81% w/w sulfuric acid (Figure S12A).

Arginine has 6 carbon atoms in its structure. The 6 carbons match the 6 carbon peaks visible in the ^13^C NMR spectrum (Figure S12B). The most downfield shifted peak around 172.4 ppm corresponds to the carboxyl group carbon. The second downfield peak at 157.16 ppm corresponds to the carbon of the guanidine group of the arginine side chain. We can identify the alpha carbon at around 54.4 ppm which matches the alpha carbon peak in D_2_O. Other carbon spectral peaks also are consistent with the carbon peaks recorded in D_2_O (Table S2). The amino acid is stable for at least four weeks in 81% w/w sulfuric acid (Figure S12B).

Asparagine has 4 carbon atoms in its structure. The 4 carbons match the 4 carbon peaks visible in the ^13^C NMR spectrum (Figure S12C). The most downfield shifted peak at 177.32 ppm corresponds to the carbon of the side chain amide group. The second downfield peak around 169.5 ppm corresponds to the carboxyl group carbon. We can identify the alpha carbon at around 50 ppm which matches the alpha carbon peak in D_2_O. Other carbon spectral peaks also are consistent with the carbon peaks recorded in D_2_O (Table S3). We note that over time (few days to four weeks) asparagine undergoes deamidation in 81% w/w sulfuric acid, to aspartic acid, as it does so in aqueous solutions in vitro and in vivo (e.g. (Pace et al., 2013)). Deamidation is indicated by small additional peaks on the ^13^C spectrum that correspond to chemical shifts of aspartic acid carbons (Figure S27).

Aspartic acid has 4 carbon atoms in its structure. The 4 carbons match the 4 carbon peaks visible in the ^13^C NMR spectrum (Figure S12D). The most downfield shifted peak at 175.55 ppm corresponds to the carbon of the side chain carboxyl group. The second downfield peak around 170.76 ppm corresponds to the carboxyl group carbon. We can identify the alpha carbon at 50.76 ppm which matches the alpha carbon peak in D_2_O. Other carbon spectral peaks also are consistent with the carbon peaks recorded in D_2_O (Table S4). The amino acid is stable for at least four weeks in 81% w/w sulfuric acid (Figure S12D).

Cysteine has 3 carbon atoms in its structure. The 3 carbons match the 3 carbon peaks visible in the ^13^C NMR spectrum (Figure S13A). The downfield peak around 170 ppm corresponds to the carboxyl group carbon. We can identify the alpha carbon at around 54 ppm which matches the alpha carbon peak in D_2_O. The carbon peak C3 adjacent to the cysteine thiol group is significantly shifted downfield, as compared to C3 peak in D_2_O (Table S5). The downfield shift is consistent with the deshielding of the C3 carbon due to the *S*-sulfation of the thiol group in 81% w/w sulfuric acid to cysteine *S*-sulfate. In contrast to *S*-sulfation in 98% w/w the *S*-sulfation in 81% w/w proceeds gradually over time, as indicated by multiple peaks around C1, C2 and C3. The sulfation of the side chain thiol group does not lead to the instability and reactivity of the entire amino acid structure and can be classified as a side-chain modification. See also discussion of the *S*-sulfation of cysteine in 98% w/w sulfuric acid in section S1.

Glutamine has 5 carbon atoms in its structure. The 5 carbons match the 5 carbon peaks visible in the ^13^C NMR spectrum (Figure S13B). The most downfield shifted peak at 181.29 ppm corresponds to the carbon of the side chain amide group. The second downfield peak at 171.09 ppm corresponds to the carboxyl group carbon. We can identify the alpha carbon at around 53.3 ppm which matches the alpha carbon peak in D_2_O. Other carbon spectral peaks also are consistent with the carbon peaks recorded in D_2_O (Table S6). We note that over time (around four weeks) glutamine undergoes deamidation in 81% w/w sulfuric acid, to glutamic acid, as it does so in aqueous solutions in vitro and in vivo (e.g. (Riggs et al., 2019)). Deamidation is indicated by small additional peaks on the ^13^C spectrum, taken after around four weeks, that correspond to chemical shifts of glutamic acid carbons (Figure S27).

Glutamic acid has 5 carbon atoms in its structure. The 5 carbons match the 5 carbon peaks visible in the ^13^C NMR spectrum (Figure S13C). The most downfield shifted peak at 180.57 ppm corresponds to the carbon of the side chain carboxyl group. The second downfield peak at 171.89 ppm corresponds to the carboxyl group carbon. We can identify the alpha carbon at 53.69 ppm which matches the alpha carbon peak in D_2_O. Other carbon spectral peaks also are consistent with the carbon peaks recorded in D_2_O (Table S7). The amino acid is stable for at least four weeks in 81% w/w sulfuric acid (Figure S13C).

Glycine has 2 carbon atoms in its structure. The 2 carbons match the 2 carbon peaks visible in the ^13^C NMR spectrum (Figure S13D). The downfield peak around 171 ppm corresponds to the carboxyl group carbon. The second peak at around 42 ppm also matches the corresponding carbon peak in D_2_O (Table S8). The amino acid is stable for at least four weeks in 81% w/w sulfuric acid (Figure S13D).

Histidine has 6 carbon atoms in its structure. The 6 carbons match the 6 carbon peaks visible in the ^13^C NMR spectrum (Figure S14A). The downfield peak at 170.44 ppm corresponds to the carboxyl group carbon. The other 3 downfield peaks, C2, C3, C4, in the “aromatic region” of the spectrum correspond to the 3 carbons of the imidazole ring. We can identify the alpha carbon at 53.42 ppm which matches the alpha carbon peak in D_2_O. Other carbon spectral peaks also are consistent with the carbon peaks recorded in D_2_O (Table S9). The amino acid is stable for at least four weeks in 81% w/w sulfuric acid (Figures S14A, S24A).

Isoleucine has 6 carbon atoms in its structure. The 6 carbons match the 6 carbon peaks visible in the ^13^C NMR spectrum (Figure S14B). The downfield peak around 172 ppm corresponds to the carboxyl group carbon. We can identify the alpha carbon at around 59 ppm which matches the alpha carbon peak in D_2_O. Other carbon spectral peaks also are consistent with the carbon peaks recorded in D_2_O (Table S10). The amino acid is stable for at least four weeks in 81% w/w sulfuric acid (Figures S14B, S24B).

Leucine has 6 carbon atoms in its structure. The 6 carbons match the 6 carbon peaks visible in the ^13^C NMR spectrum (Figure S14C). The downfield peak around 173 ppm corresponds to the carboxyl group carbon. The symmetry of the two carbons C5 in the leucine side chain is broken but it matches the peaks recorded in D_2_O. We can identify the alpha carbon at around 53.5 ppm which matches the alpha carbon peak in D_2_O. Other carbon spectral peaks also are consistent with the carbon peaks recorded in D_2_O (Table S11). The amino acid is stable for at least four weeks in 81% w/w sulfuric acid (Figures S14C, S24C).

Lysine has 6 carbon atoms in its structure. The 6 carbons match the 6 carbon peaks visible in the ^13^C NMR spectrum (Figure S14D). The downfield peak around 172.5 ppm corresponds to the carboxyl group carbon. We can identify the alpha carbon at around 54.5 ppm which matches the alpha carbon peak in D_2_O. Other carbon spectral peaks also are consistent with the carbon peaks recorded in D_2_O (Table S12). The amino acid is stable for at least four weeks in 81% w/w sulfuric acid (Figures S14C, S24D).

Methionine has 5 carbon atoms in its structure. The 5 carbons match the number of carbon peaks visible in the ^13^C NMR spectrum (Figure S15A) collected after 12-18h. The additional, smaller, peaks that emerge after several days are likely a result of the demethylation of the sulfur atom in the methionine side chain (Andrews and Bruce, 1951; Butz and du Vigneaud, 1932), and possibly subsequent sulfation of the thiol group (see also discussion of the possible demethylation and the *S*-sulfation of homocysteine in 98% w/w sulfuric acid in section S1 above). The downfield peak at 172.08 ppm corresponds to the carboxyl group carbon. We can identify the alpha carbon at 53.82 ppm which matches the alpha carbon peak in D_2_O (Table S13).

Phenylalanine has 9 carbon atoms in its structure. The 9 carbons of phenylalanine match the carbon peaks visible in the ^13^C NMR spectrum (Figure S15B). In contrast to the ^13^C NMR results in 98% w/w sulfuric acid (Figure S10B) phenylalanine in 81% w/w appears to be stable and unreactive. The carbon atoms of the side chain aromatic ring of phenylalanine give clear signals in the “aromatic” region of the spectrum, consistent with the carbon peaks recorded in D_2_O (Table S14). The downfield peak around 172 ppm corresponds to the carboxyl group carbon. We note that in contrast to phenylalanine in 98% w/w, which likely gets sulfonated in 98% w/w sulfuric acid (Figure S10B), in 81% w/w we do not get sulfonation. Phenylalanine is stable and unchanged for at least four weeks in 81% w/w sulfuric acid (Figures S15B, S25B), ruling out spontaneous sulfonation as a plausible modification of aromatic rings in 81% w/w.

Proline has 5 carbon atoms in its structure. The 5 carbons match the 5 carbon peaks visible in the ^13^C NMR spectrum (Figure S15C). The downfield peak around 173 ppm corresponds to the carboxyl group carbon. We can identify the alpha carbon at around 61 ppm which matches the alpha carbon peak in D_2_O. Other carbon spectral peaks also are consistent with the carbon peaks recorded in D_2_O (Table S15). The amino acid is stable for at least four weeks in 81% w/w sulfuric acid (Figures S15B, S24B).

Serine has 3 carbon atoms in its structure. The 3 carbons match the 3 carbon peaks visible in the ^13^C NMR spectrum (Figure S15D), although we note that the hydroxyl group of serine is likely sulfated. Sulfation of serine agrees with previous studies on sulfation of aliphatic alcohols in concentrated sulfuric acid (Iraci et al., 2002; Kane and Leu, 2001) and the early NMR studies on sulfoserine and sulfothreonine (Rose et al., 1994). There are two distinct carbon peaks that correspond to carbon C2 that is adjacent to the serine hydroxyl (OH) group. One of the two peaks is significantly shifted downfield as compared to the C2 peak in D_2_O (Table S16). The downfield shift is consistent with the deshielding of the C2 carbon due to the sulfation of the serine OH group in 81% w/w sulfuric acid. The sulfation appears to be less efficient in 81% than in 98% w/w (Figure S10D). Sulfation does not result in the instability of the entire amino acid structure, rather it can be classified as chemical modification of the amino acid side chain. The downfield peak around 170 ppm corresponds to the carboxyl group carbon. We can identify the alpha carbon at around 55 ppm which matches the alpha carbon peak in D_2_O (Table S16).

Threonine has 4 carbon atoms in its structure. The 4 carbons match the 4 carbon peaks visible in the ^13^C NMR spectrum (Figure S16A), although we note that the hydroxyl group of threonine is likely sulfated. Sulfation of threonine agrees with previous studies on sulfation of aliphatic alcohols in concentrated sulfuric acid (Iraci et al., 2002; Kane and Leu, 2001) and the early NMR studies on sulfoserine and sulfothreonine (Rose et al., 1994). There are two distinct carbon peaks that correspond to carbon C2 that is adjacent to the threonine hydroxyl (OH) group. One of the two peaks is significantly shifted downfield as compared to the C2 peak in D_2_O (Table S17). The downfield shift is consistent with the deshielding of the C2 carbon due to the sulfation of the threonine OH group in 81% w/w sulfuric acid. The sulfation appears to be less efficient in 81% than in 98% w/w (Figure S11A). Sulfation does not result in the instability of the entire amino acid structure, rather it can be classified as chemical modification of the amino acid side chain. The downfield peak around 170 ppm corresponds to the carboxyl group carbon. We can identify the alpha carbon at around 59 ppm which matches the alpha carbon peak in D_2_O (Table S17).

Tryptophan has 11 carbon atoms in its structure. The ^13^C NMR spectrum shows that, similarly to the results obtained for 98% w/w, tryptophan is unstable and highly reactive in 81% w/w sulfuric acid (Figure S16B).

Tyrosine has 9 carbon atoms in its structure, 7 of which are unique. Similarly to ^13^C NMR in 98% w/w, the 7 unique carbons do not match the 9 carbon peaks visible in the ^13^C NMR spectrum recorded in 81% w/w acid (Figure S16C). The downfield peak around 172 ppm corresponds to the carboxyl group carbon. We can identify the alpha carbon at around 55 ppm which matches the alpha carbon peak in D_2_O (Table S19) and can also tentatively assign carbon C7 to peak 35 ppm (Table S19). These assignments further confirm the stability of the amino acid backbone structure and support the conclusion that any reactivity of tyrosine is confined to the side chain aromatic ring. The sulfation and sulfonation of the ring could explain the 6 carbon peaks in the “aromatic region” of the spectrum. We note that in aqueous solutions tyrosine is oxidized, resulting in the addition of hydroxyl groups (OH) to the phenyl ring of tyrosine. Example of such oxidation is the formation of 3,4-dihydroxyphenylalanine (DOPA) or 3,4,5-trihydroxyphenylalanine (TOPA) (Burzio and Waite, 2002; Recky et al., 2021). Whether similar oxidation in concentrated sulfuric acid happens remains to be seen.

Valine has 5 carbon atoms in its structure. The 5 carbons match the 5 carbon peaks visible in the ^13^C NMR spectrum (Figure S16D). The downfield peak around 172.4 ppm corresponds to the carboxyl group carbon. The symmetry of the two C4 carbons in the side chain is broken but it matches the peaks recorded in D_2_O. We can identify the alpha carbon at around 60 ppm which matches the alpha carbon peak in D_2_O. Other carbon spectral peaks also are consistent with the carbon peaks recorded in D_2_O (Table S20). The amino acid is stable for at least four weeks in 81% w/w sulfuric acid (Figures S16D, S26D).

**S3. Molecular Structure Determination of Biogenic Amino Acids with 2D ^1^H-^13^C HMQC NMR in 98% w/w Concentrated Sulfuric Acid.**

We further confirm the integrity of the structure of each of the individual biogenic amino acids in concentrated sulfuric acid with 2D ^1^H-^13^C HMQC NMR (Figures S1-S4). The 2D ^1^H-^13^C HMQC NMR technique can identify which hydrogen and carbon atoms in the analyzed molecule are directly bonded to each other in solution, therefore further confirming the integrity of the amino acid structure.

We provide a detailed description of the alanine C and H atom assignment as an example of how to interpret the 2D HMQC data (Figure S1A).

Alanine has only two carbons with directly attached hydrogen, and this is for C2 and C3. Our 2D ^1^H-^13^C HMQC shows a signal that corresponds to the C2 carbon peak at 50.95 ppm and H (attached to C2) at 4.62 ppm. The 2D ^1^H-^13^C HMQC also confirms the assignment of C3 at 15.17 ppm and H3, at 1.98 ppm, attached to C3. Our assignment agrees with the literature data collected in acidic D_2_O (Table S1).

The HMQC results (Figures S1-S4) confirm that structures of the 13 amino acids (arginine, histidine, lysine, aspartic acid, glutamic acid, asparagine, glutamine, glycine, proline, alanine, isoleucine, leucine, valine) remain stable and unchanged upon dissolution in 98% w/w concentrated sulfuric acid at room temperature. The HMQC results also confirm the overall structures of serine, threonine and cysteine. The 3 amino acids (cysteine, serine and threonine) are stable but undergo chemical modification of the side chain in concentrated sulfuric acid.

We note that we have not analyzed in detail the 2D ^1^H-^13^C HMQC NMR spectra of methionine, tyrosine, phenylalanine and tryptophan as those four amino acids likely undergo complex modification and reactivity in concentrated sulfuric acid. We do however, for completeness, provide the original 2D ^1^H-^13^C HMQC NMR data for all 20 amino acids as Supplementary Dataset S2.

**S4. Stability of the Biogenic Amino Acids in 98% and 81% w/w Concentrated Sulfuric Acid After Four Weeks Incubation.**

To confirm the long-term stability of all 20 biogenic amino acids in concentrated sulfuric acid we have incubated 30 mg of each amino acid in 81% w/w and 98% w/w D_2_SO_4_ for four weeks, stored in the NMR tubes with room temperature varying from about 18 to 24 °C. After the four weeks incubation we acquired 1D ^13^C NMR spectra of each amino acid, at each of the sulfuric acid concentrations, and compared them to the original 1D ^13^C NMR spectra collected after 12-18 h and 5-8 days.

The 12-18 h and the four weeks NMR spectra look virtually identical for all tested amino acids with the exception of asparagine and glutamine in 81% w/w concentrated sulfuric acid that get converted to aspartic acid and glutamic acid respectively. Reactive amino acids (methionine, cysteine, serine, threonine, tyrosine, tryptophan) appear to react after 12-18 h, and again only the side chains undergo chemical modification.

The NMR spectra collected after four weeks confirm the long-term stability and lack of reactivity of arginine, histidine, lysine, aspartic acid, glutamic acid, glycine, proline, alanine, isoleucine, leucine, valine amino acids in concentrated sulfuric acid solvent (Figure S5 and Figures S6).

We have also used the 2D ^1^H-^13^C HMQC NMR (Figures S1-S4), as well as ^1^H NMR spectra collected in D_2_O from the literature for comparison (Saito et al., 2006), to assign the H atoms of the tested amino acids in concentrated sulfuric acid. As for ^13^C NMR spectra, after the four weeks incubation we acquired 1D ^1^H NMR spectra of each amino acid, at each of the sulfuric acid concentrations, and compared them to the original 1D ^1^H NMR spectra collected after 12-18 h and 5-8 days (Figure S17-S26).

The results of the ^1^H NMR experiment are consistent with the conclusions of the ^13^C NMR results confirm the long-term stability and lack of reactivity of arginine, histidine, lysine, aspartic acid, glutamic acid, glycine, proline, alanine, isoleucine, leucine, valine amino acids in concentrated sulfuric acid solvent. We provide original 1D ^1^H and ^13^C NMR data collected in 81% w/w and 98% w/w at all three time intervals for all 20 amino acids as Supplementary Dataset S1.

**S5. Stability of the Biogenic Amino Acids Glycine and Alanine in 98% and 81% w/w Concentrated Sulfuric Acid in the Presence of Formic Acid and Iron(II) Oxide (FeO).**

The Venus cloud aerosols are not composed of pure concentrated sulfuric acid. A variety of gases, metal ions, and other non-volatile compounds are dissolved in liquid concentrated sulfuric acid droplets, making the cloud aerosols a chemically complex mixture (Mogul et al., 2021; Zolotov et al., 2023). To test if some of the trace reactive species present in Venus’ clouds react with amino acids we have took two simplest amino acids, alanine and glycine, and mixed them in 1:1 molar ratio with formic acid (H_2_CO_2_; HCOOH) and iron(II) oxide (FeO). We find that the tested amino acids are stable for at least a week in concentrated sulfuric acid mixed with these additives (Figures S33-35).

We choose formic acid as an additive for two reasons. Firstly, upon dissolution in concentrated sulfuric acid formic acid (HCOOH) readily decomposes to carbon monoxide (CO) and water (H_2_O) (Koch-Haaf reaction (1) below). Released CO can then readily react with any organic carbocations, if carbocations are present in the reaction mixture (Koch and Haaf, 1958). A carbocation is an ion with a positively charged carbon atom. Many organic molecules form carbocations upon dissolution in concentrated sulfuric acid thus, if amino acids form carbocations, the released CO should react with them. Secondly, this type of reaction is directly relevant for Venus’ cloud conditions as CO is a trace gas in Venus’ atmosphere (e.g., (Marcq et al., 2018)) and could in principle readily dissolve in the cloud droplets and react with other dissolved components. In the “test tube” conditions, at room temperature, we do not see any signs of reactivity of glycine and alanine with HCOOH and CO (Figure S33). The ^13^C and ^1^H NMR spectra clearly show the dissolved HCOOH in 98% w/w acid. HCOOH shows up at 175.22 ppm and 8.80 ppm, on ^13^C and ^1^H NMR spectra respectively, while its decomposition product, the dissolved CO, is visible at 184.40 ppm on ^13^C NMR spectra (Figure S33). The ^13^C and ^1^H NMR peak assignments for HCOOH and CO have been carried out by comparison to the literature chemical shift values for HCOOH (^1^H: 8.18 ppm; ^13^C: 166.80 ppm (Ponder and Richards, 1993)) and CO (^13^C: 184.0 ppm (Stoebenau and Jordan, 2003)). The decomposition of HCOOH to CO appears to be complete after one week, with CO outgassing from the solution, leaving intact alanine and glycine (Figure S33). We note that we have performed our reactivity test at room temperature, under ambient pressure conditions. Reactivity tests under high temperature (around 100 °C) corresponding to the very bottom of the cloud deck is a topic of a separate study.

 $(1)$

Iron(II) Oxide (FeO) is a likely component of Venusian surface rocks, as reported by the Venera and VeGa landers measurements (Surkov et al., 1986, 1984). Minerals containing FeO can in principle be lifted from the surface or delivered to the clouds via meteoritic infall where they can dissolve in sulfuric acid droplets and react with other dissolved components. Iron(II) is a reduced, reactive form of iron often thought, in the form of pyrite (FeS_2_), to be instrumental in the origin of life on Earth (e.g. (Popper, 1990))^[[2]](#footnote-2)^. We therefore asked if the mineral FeO mixed with dissolved amino acids glycine and alanine would promote their reactivity in concentrated sulfuric acid. Our ^13^C and ^1^H NMR experiments (Figure S34-35) demonstrate that after one-week incubation in concentrated sulfuric acid we do not see any reactivity of tested amino acids in the presence of concentrated sulfuric acid mixed with FeO solid. Note that, as expected, the addition of the FeO to the concentrated sulfuric acid results in broadening of the NMR peaks. Such broadening and suppression of the signal is especially pronounced in the carboxyl carbon peak on the ^13^C NMR spectra. FeO is a known doping agent that enhances relaxation (e.g. (Jeon et al., 2021)) which is responsible for the reduced intensity of the carbonyl peak and the general broadening of the other peaks. The carboxyl carbon peak broadening and suppression is not indicative of detrimental reactivity, as all other expected peaks of alanine and glycine, in both ^13^C and ^1^H NMR experiments, are accounted for, and no new peaks emerge as a result of addition of FeO to the mixture.


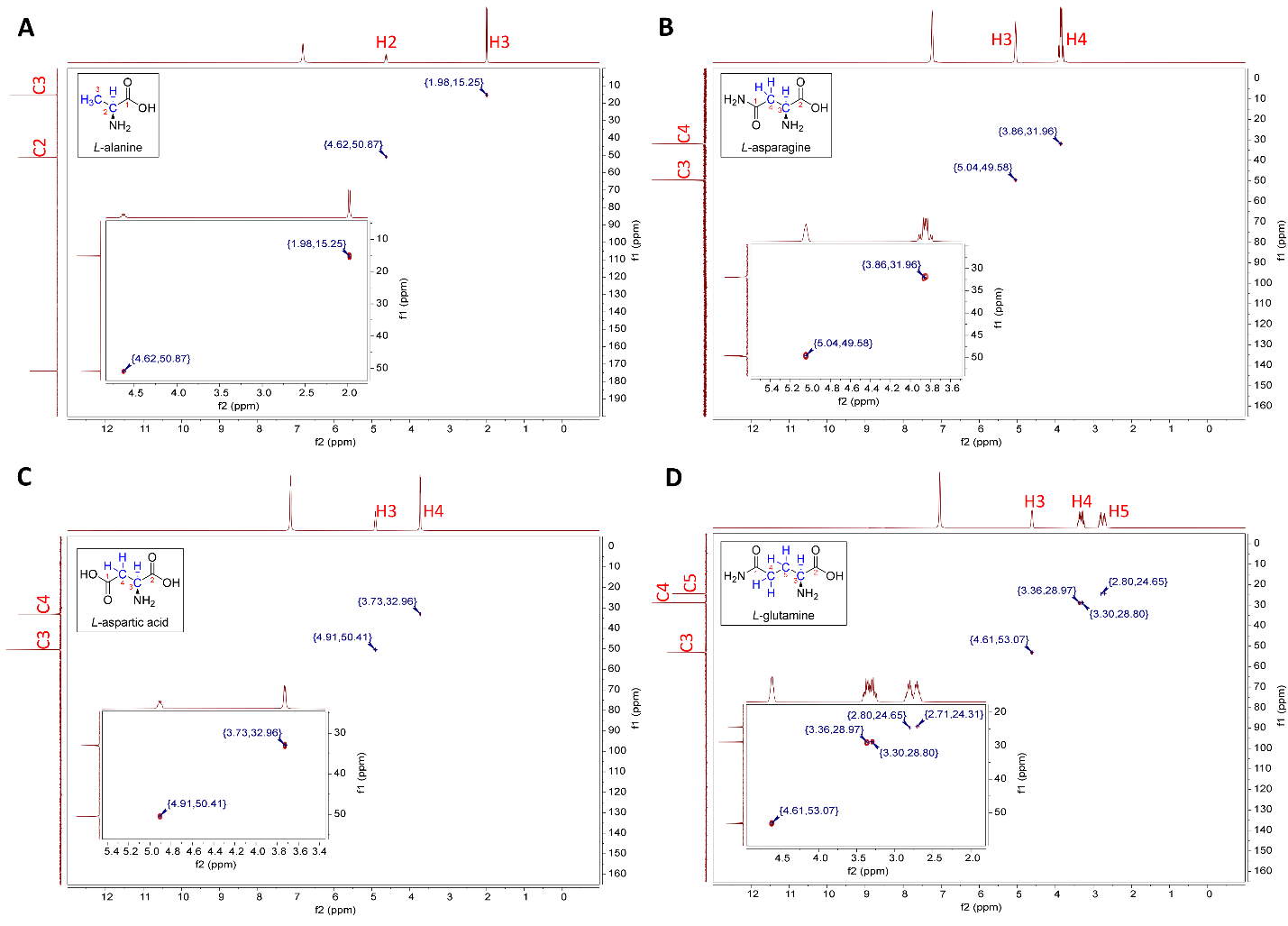
**Figure S1.** NMR spectra for biogenic amino acids: alanine, asparagine, aspartic acid and glutamine in concentrated sulfuric acid (98% D_2_SO_4_ and 2% D_2_O, by weight) at room temperature after 12-18 h incubation. The solvent signal is suppressed for clarity. The f1 axis: 1D ^13^C, the f2 axis: 1D ^1^H. The 2D ^1^H-^13^C HMQC NMR shows direct bonding between H and C atoms in the amino acid structure (marked in blue in the illustrative amino acid structure). The NMR experiments confirm the stability of the tested amino acids in 98% w/w sulfuric acid. **A)** The 2D ^1^H-^13^C HMQC NMR of alanine. **B)** The 2D ^1^H-^13^C HMQC NMR of asparagine. **C)** The 2D ^1^H-^13^C HMQC NMR of aspartic acid. **D)** The 2D ^1^H-^13^C HMQC NMR of glutamine. The ^13^C assignments (left axis) come from comparison with the literature values from D_2_O (Tables S1-S20; (Saito et al., 2006)). The ^1^H assignments (top axis) also come from D_2_O experiments (Tables S1-S20; (Saito et al., 2006)). The identification of H-C bonds comes from the “spots” at the intersection of the ^1^H and ^13^C axes. Because the relationships between H and C atoms derived from the 2D NMR match the designations in the molecular structure (top left inset), this confirms the structure and hence stability of the tested amino acids in 98% w/w concentrated sulfuric acid. For consistency the C atoms have been numbered accordingly to the convention used in (Saito et al., 2006).


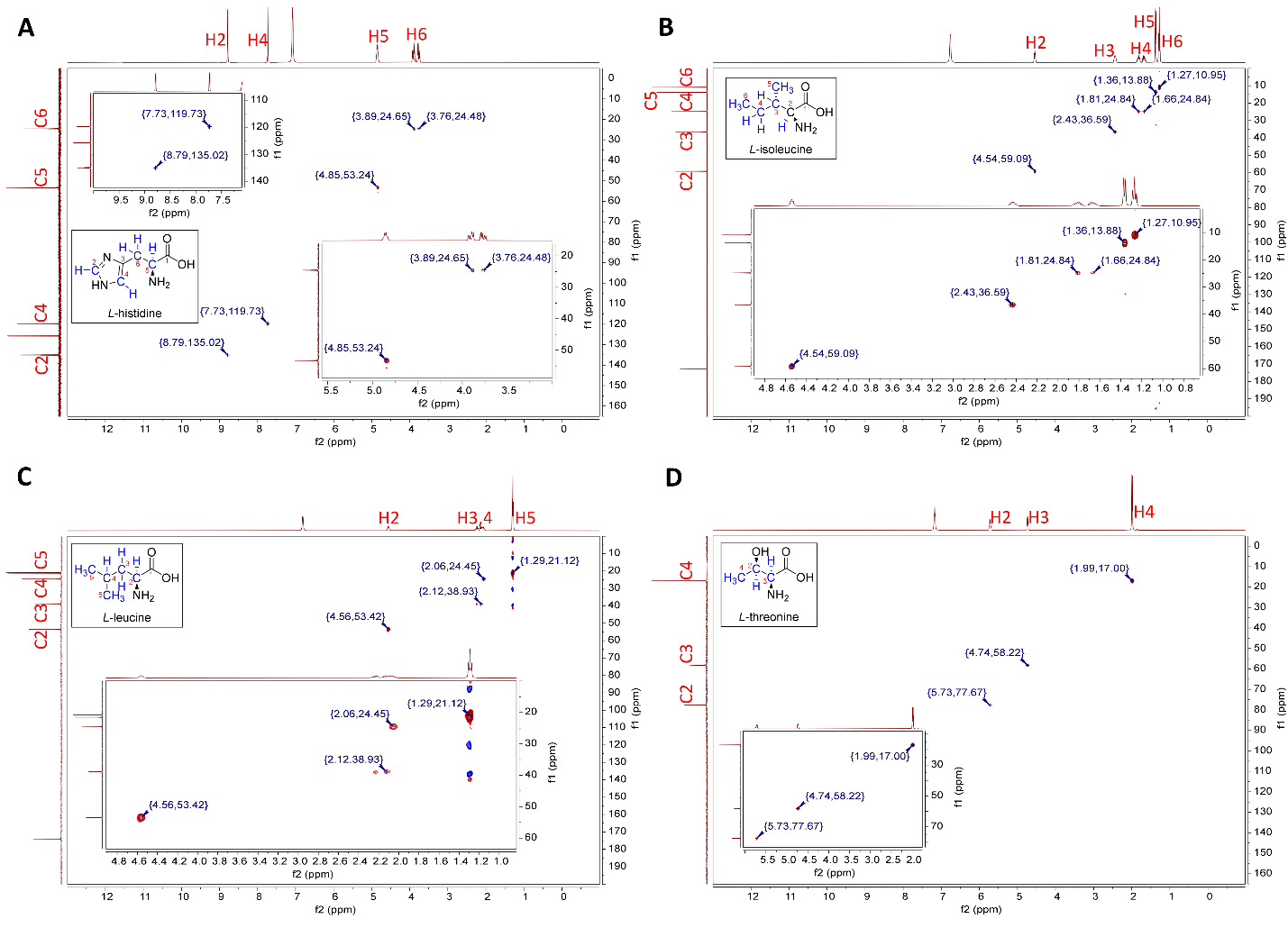
**Figure S2.** NMR spectra for biogenic amino acids: histidine, isoleucine, leucine and threonine in concentrated sulfuric acid (98% D_2_SO_4_ and 2% D_2_O, by weight) at room temperature after 12-18 h incubation. The solvent signal is suppressed for clarity. The f1 axis: 1D ^13^C, the f2 axis: 1D ^1^H. The 2D ^1^H-^13^C HMQC NMR shows direct bonding between H and C atoms in the amino acid structure (marked in blue in the illustrative amino acid structure). The NMR experiments confirm the stability of the tested amino acids in 98% w/w sulfuric acid. **A)** The 2D ^1^H-^13^C HMQC NMR of histidine. **B)** The 2D ^1^H-^13^C HMQC NMR of isoleucine. **C)** The 2D ^1^H-^13^C HMQC NMR of leucine. **D)** The 2D ^1^H-^13^C HMQC NMR of threonine. Note that threonine side chain hydroxyl (OH) group is likely sulfated in concentrated sulfuric acid. The ^13^C assignments (left axis) come from comparison with the literature values from D_2_O (Tables S1-S20; (Saito et al., 2006)). The ^1^H assignments (top axis) also come from D_2_O experiments (Tables S1-S20; (Saito et al., 2006)). The identification of H-C bonds comes from the “spots” at the intersection of the ^1^H and ^13^C axes. Because the relationships between H and C atoms derived from the 2D NMR match the designations in the molecular structure (top left inset), this confirms the structure and hence stability of the tested amino acids in 98% w/w concentrated sulfuric acid. For consistency the C atoms have been numbered accordingly to the convention used in (Saito et al., 2006).


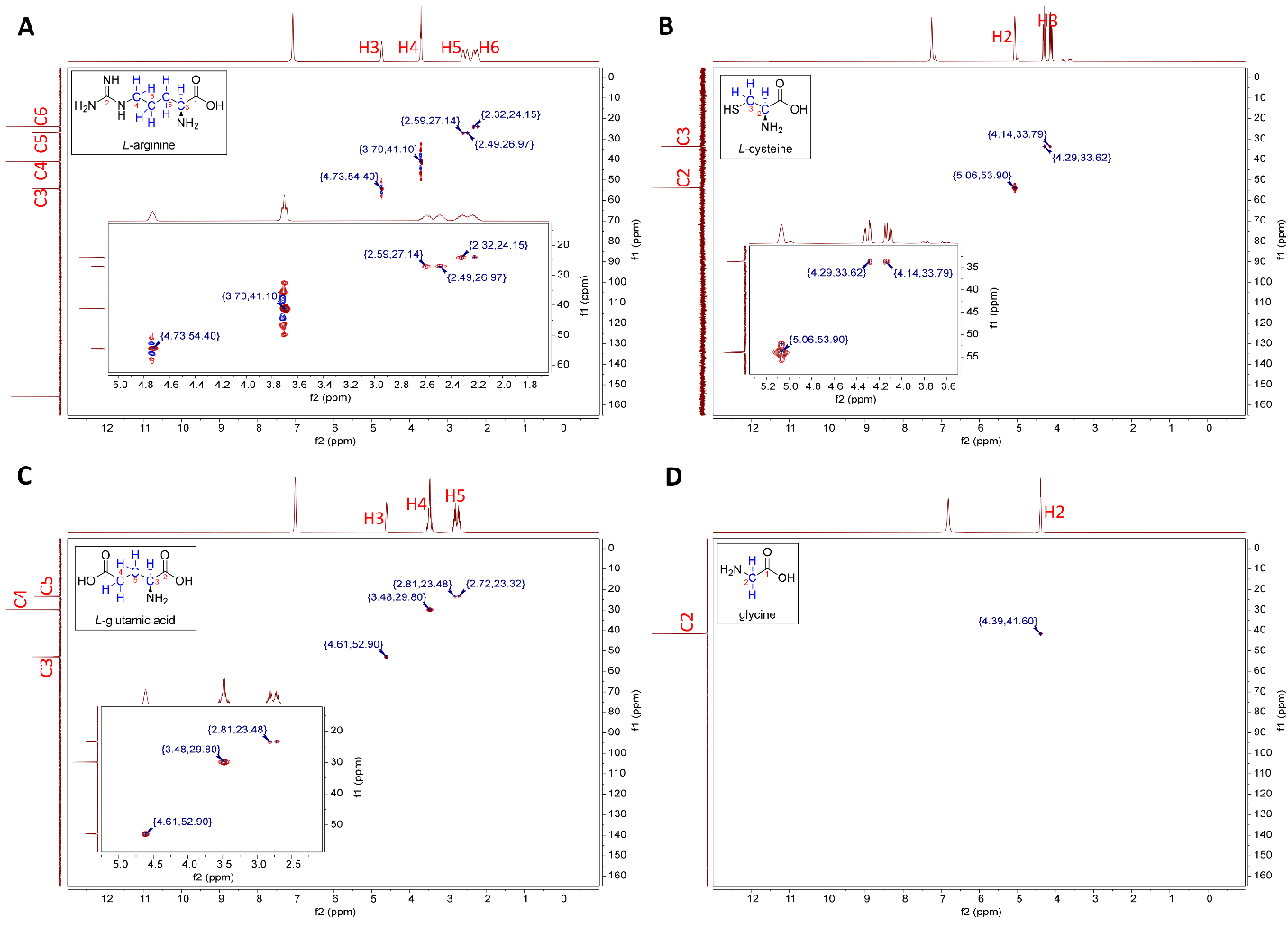
**Figure S3.** NMR spectra for biogenic amino acids: arginine, cysteine, glutamic acid and glycine in concentrated sulfuric acid (98% D_2_SO_4_ and 2% D_2_O, by weight) at room temperature after 5-8 day incubation. The solvent signal is suppressed for clarity. The f1 axis: 1D ^13^C, the f2 axis: 1D ^1^H. The 2D ^1^H-^13^C HMQC NMR shows direct bonding between H and C atoms in the amino acid structure (marked in blue in the illustrative amino acid structure). The NMR experiments confirm the stability of the tested amino acids in 98% w/w sulfuric acid. **A)** The 2D ^1^H-^13^C HMQC NMR of arginine. **B)** The 2D ^1^H-^13^C HMQC NMR of cysteine. Note that cysteine side chain sulfhydryl (SH) group is sulfated in concentrated sulfuric acid. **C)** The 2D ^1^H-^13^C HMQC NMR of glutamic acid. **D)** The 2D ^1^H-^13^C HMQC NMR of glycine. The ^13^C assignments (left axis) come from comparison with the literature values from D_2_O (Tables S1-S20; (Saito et al., 2006)). The ^1^H assignments (top axis) also come from D_2_O experiments (Tables S1-S20; (Saito et al., 2006)). The identification of H-C bonds comes from the “spots” at the intersection of the ^1^H and ^13^C axes. Because the relationships between H and C atoms derived from the 2D NMR match the designations in the molecular structure (top left inset), this confirms the structure and hence stability of the tested amino acids in 98% w/w concentrated sulfuric acid. For consistency the C atoms have been numbered accordingly to the convention used in (Saito et al., 2006).


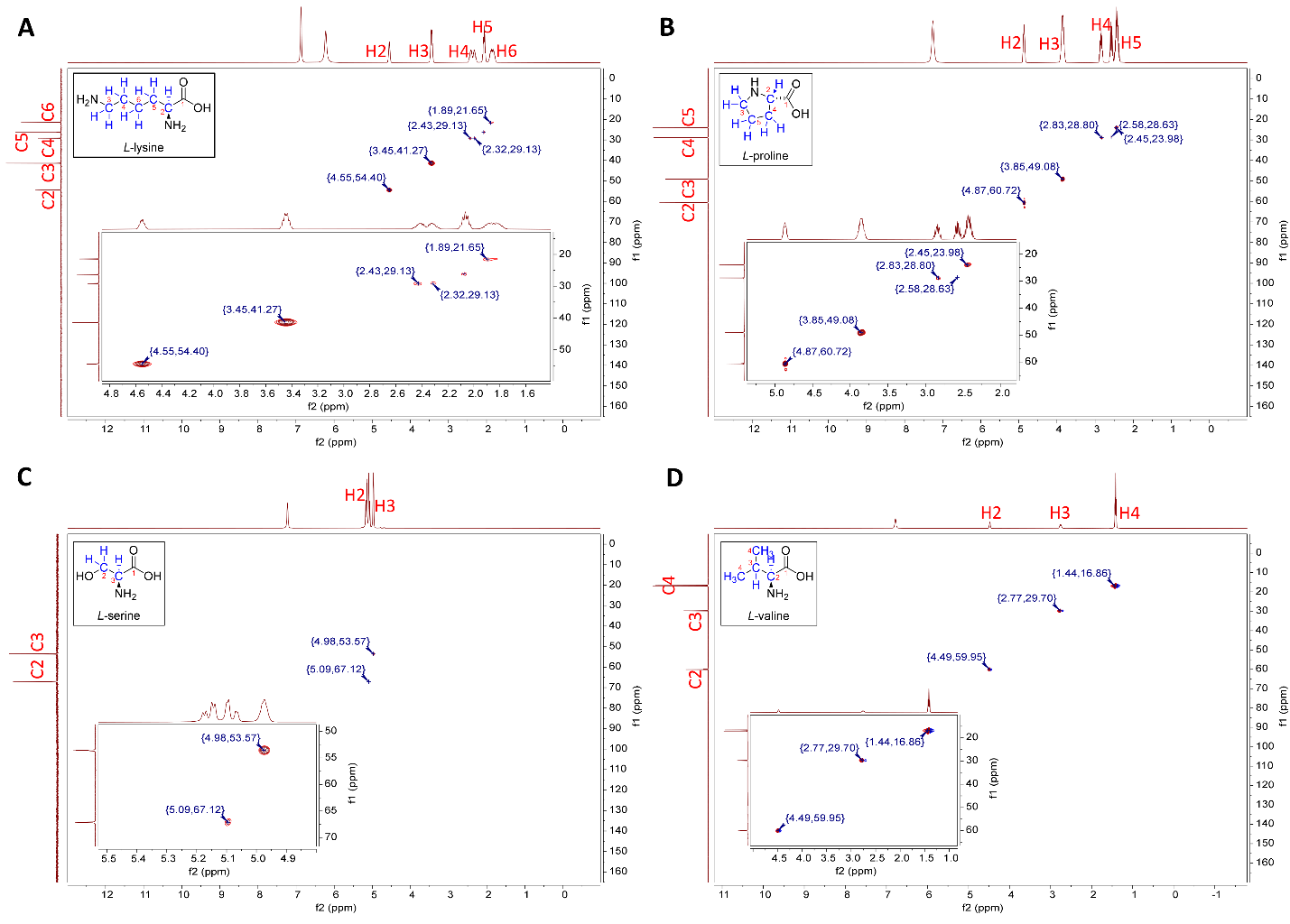
**Figure S4.** NMR spectra for biogenic amino acids: lysine, proline, serine and valine in concentrated sulfuric acid (98% D_2_SO_4_ and 2% D_2_O, by weight) at room temperature after 5-8 day incubation. The solvent signal is suppressed for clarity. The f1 axis: 1D ^13^C, the f2 axis: 1D ^1^H. The 2D ^1^H-^13^C HMQC NMR shows direct bonding between H and C atoms in the amino acid structure (marked in blue in the illustrative amino acid structure). The NMR experiments confirm the stability of the tested amino acids in 98% w/w sulfuric acid. **A)** The 2D ^1^H-^13^C HMQC NMR of lysine. **B)** The 2D ^1^H-^13^C HMQC NMR of proline. **C)** The 2D ^1^H-^13^C HMQC NMR of serine. Note that serine side chain hydroxyl (OH) group is sulfated in concentrated sulfuric acid. **D)** The 2D ^1^H-^13^C HSQC (Heteronuclear Single Quantum Coherence) NMR of valine. The ^13^C assignments (left axis) come from comparison with the literature values from D_2_O (Tables S1-S20; (Saito et al., 2006)). The ^1^H assignments (top axis) also come from D_2_O experiments (Tables S1-S20; (Saito et al., 2006)). The identification of H-C bonds comes from the “spots” at the intersection of the ^1^H and ^13^C axes. Because the relationships between H and C atoms derived from the 2D NMR match the designations in the molecular structure (top left inset), this confirms the structure and hence stability of the tested amino acids in 98% w/w concentrated sulfuric acid. For consistency the C atoms have been numbered accordingly to the convention used in (Saito et al., 2006).

**
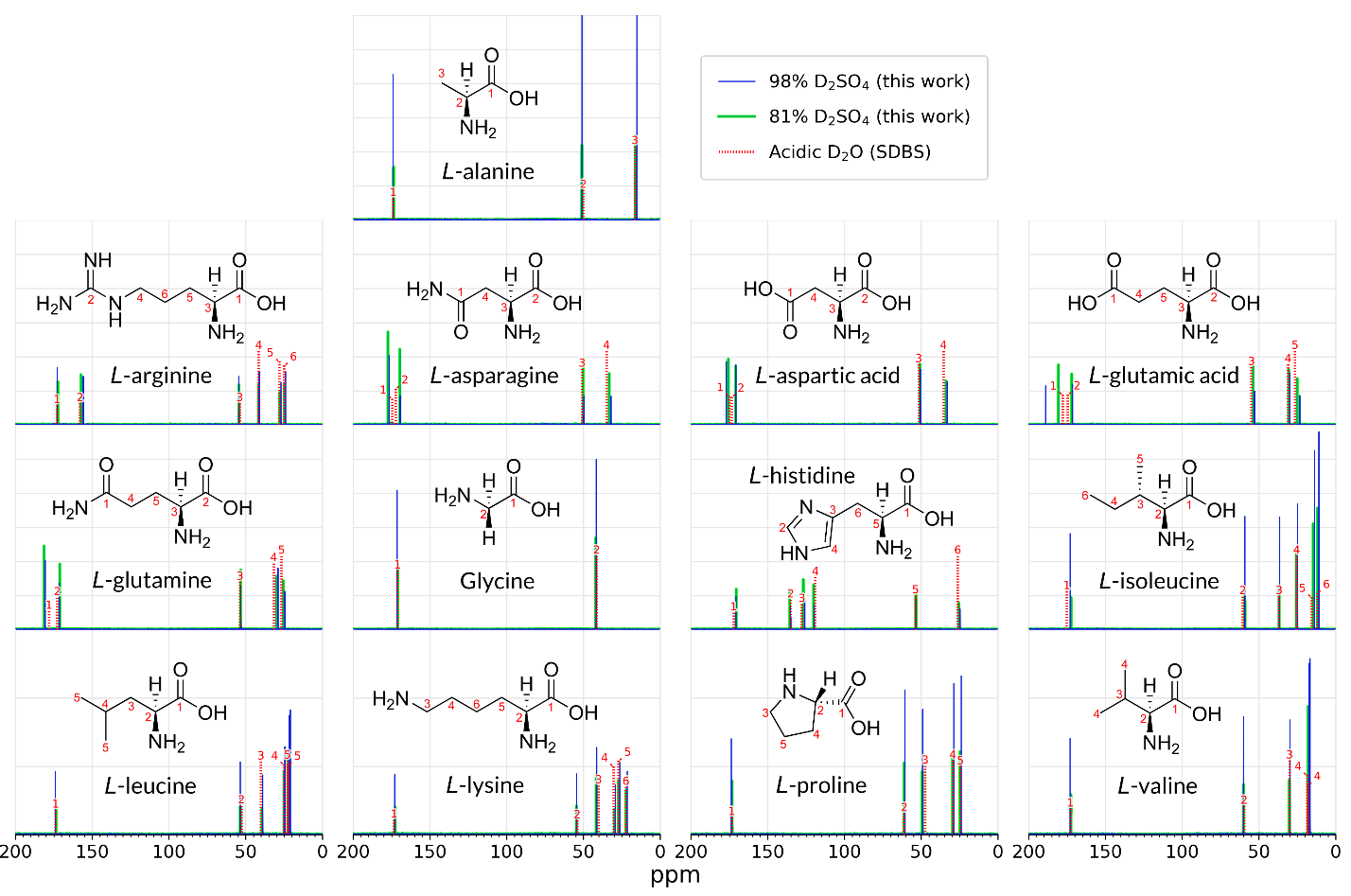
Figure S5.** Biogenic amino acids stable in 98% w/w concentrated sulfuric acid. Each panel shows the ^13^C NMR spectral peak intensities as a function of shifts in ppm for biogenic amino acids (as labeled) after 12 to 18 hours in concentrated sulfuric acid at room temperature. The two different concentrations, 98% w/w D_2_SO_4_ w/w (blue) and 81% w/w sulfuric acid (green) with the rest D_2_O, span the range of sulfuric acid concentrations in the Venus clouds. We show ^13^C NMR spectral peak shifts for the same amino acids in acidic^^[[3]](#footnote-3)^^ D_2_O (red) from literature values (Saito et al., 2006). The spectral peak shifts for both sulfuric acid concentrations agree with the chemical shift values in acidic water (pH 1-3), demonstrating the stability of the amino acids in concentrated sulfuric acid. Out of the amino acids in this figure, only asparagine and glutamine are modified, and only in 81% w/w sulfuric acid. SDBS is the Spectral Database for Organic Compounds (Saito et al., 2006).

**
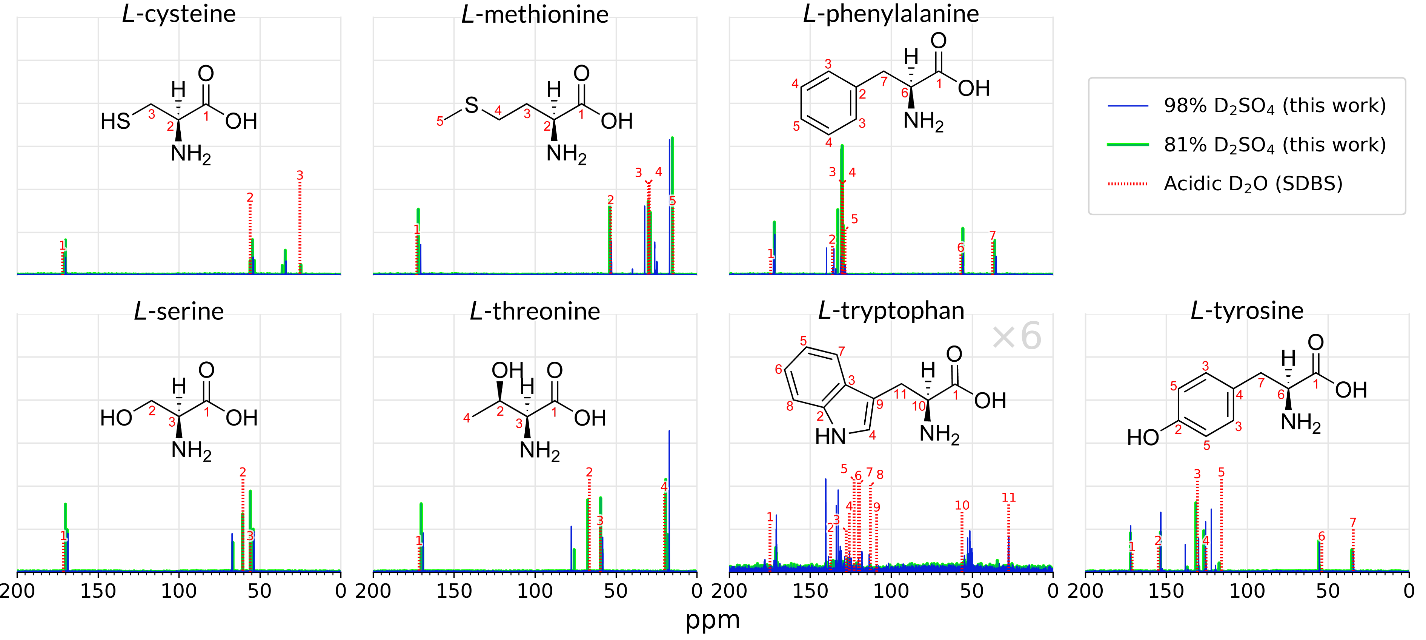
Figure S6.** Biogenic amino acids modified or unstable in concentrated sulfuric acid. Each panel shows the ^13^C NMR spectral peak intensities as a function of shifts in ppm for biogenic amino acids (as labeled) after 12 to 18 hours in concentrated sulfuric acid at room temperature. The two different concentrations, 98% w/w D_2_SO_4_ w/w (blue) and 81% w/w sulfuric acid w/w (green) with the rest D_2_O, span the range of sulfuric acid concentrations in the Venus clouds. We show ^13^C NMR spectral peak shifts for the same amino acids in acidic^^[[4]](#footnote-4)^^ D_2_O (red) from literature values (Saito et al., 2006). The spectral peak shifts for both concentrations disagree with the chemical shift values in acidic water (pH 1-3), demonstrating the reactivity of this set amino acids in concentrated sulfuric acid. Tryptophan intensities in sulfuric acid are multiplied by 6 so the peaks are visible at the scale of the figure. SDBS is the Spectral Database for Organic Componds (Saito et al., 2006).
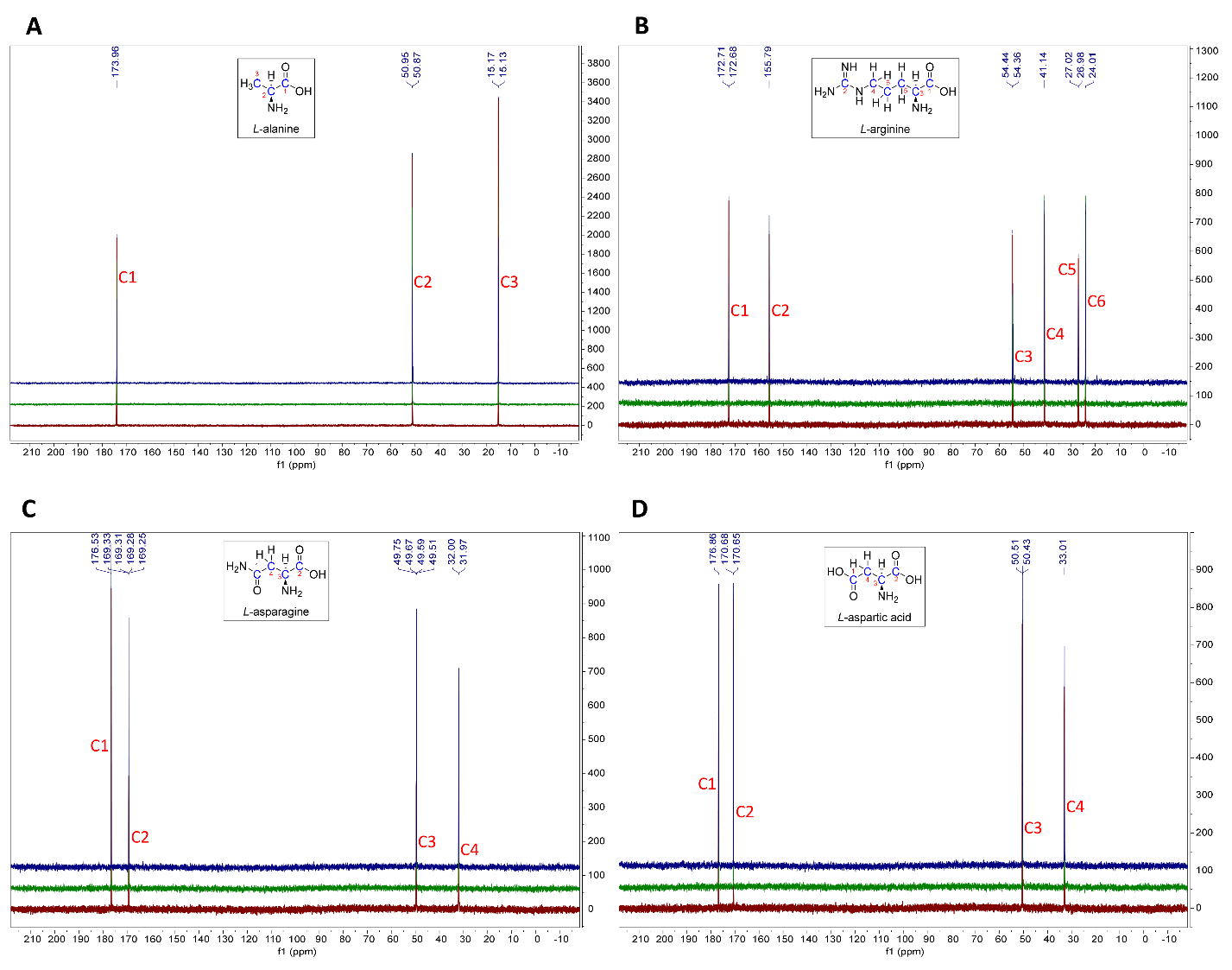
**Figure S7.** The comparison of the ^13^C NMR spectra for biogenic amino acids: alanine, arginine, asparagine, aspartic acid in concentrated sulfuric acid (98% D_2_SO_4_ and 2% D_2_O, by weight), at room temperature, collected after 12-18h incubation (red spectra), 5-8 day incubation (green spectra) to spectra collected after four week incubation (blue spectra). **A)** The ^13^C NMR of alanine. **B)** The ^13^C NMR of arginine. **C)** The ^13^C NMR of asparagine. **D)** The ^13^C NMR of aspartic acid. For consistency the C atoms on the illustrative structures of amino acids have been numbered accordingly to the convention used in (Saito et al., 2006).


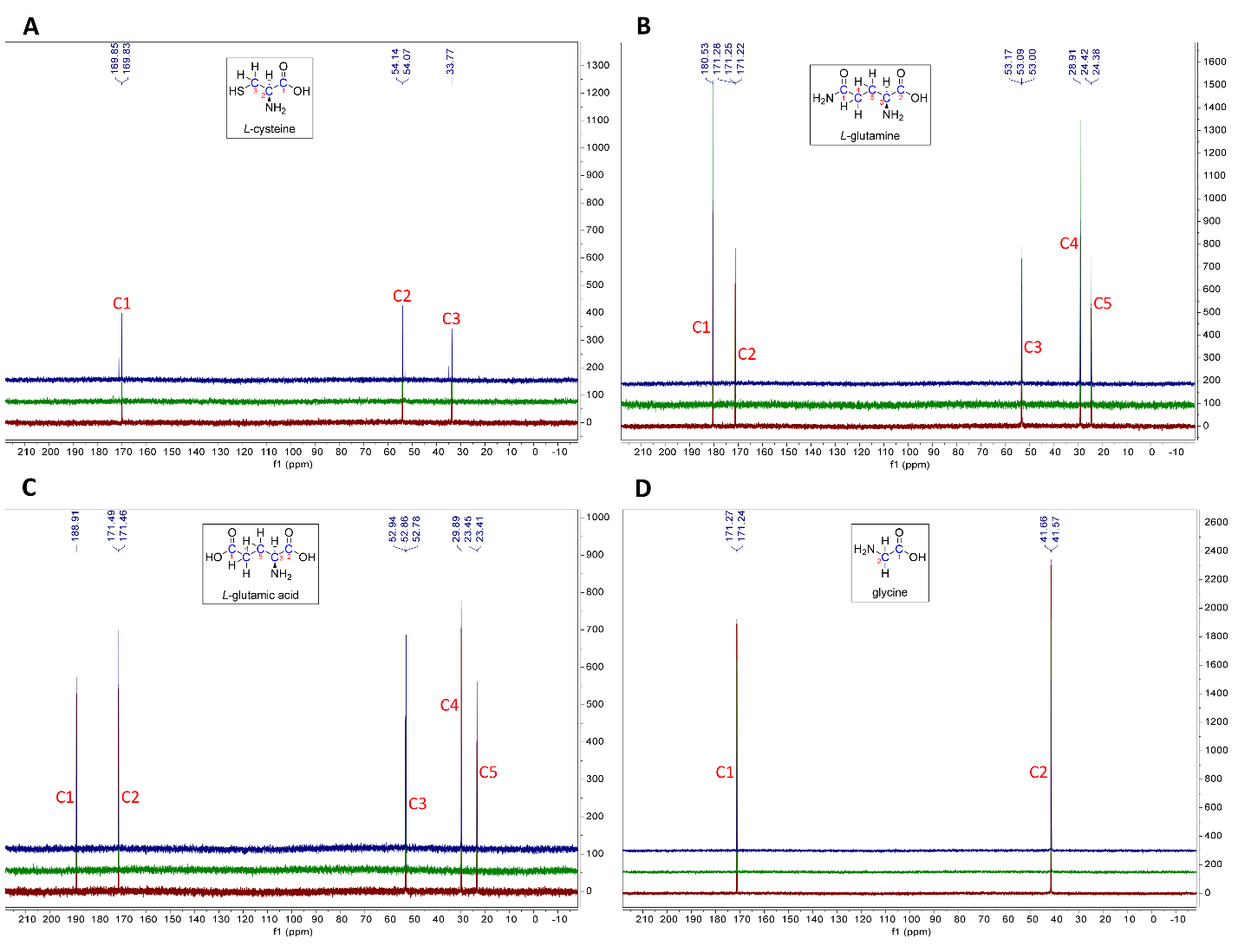
**Figure S8.** The comparison of the ^13^C NMR spectra for biogenic amino acids: cysteine, glutamine, glutamic acid, glycine in concentrated sulfuric acid (98% D_2_SO_4_ and 2% D_2_O, by weight), at room temperature, collected after 12-18h incubation (red spectra), 5-8 day incubation (green spectra) to spectra collected after four week incubation (blue spectra). **A)** The ^13^C NMR of cysteine. Note that cysteine is sulfated in concentrated sulfuric acid. **B)** The ^13^C NMR of glutamine. **C)** The ^13^C NMR of glutamic acid. **D)** The ^13^C NMR of glycine. For consistency the C atoms on the illustrative structures of amino acids have been numbered accordingly to the convention used in (Saito et al., 2006).


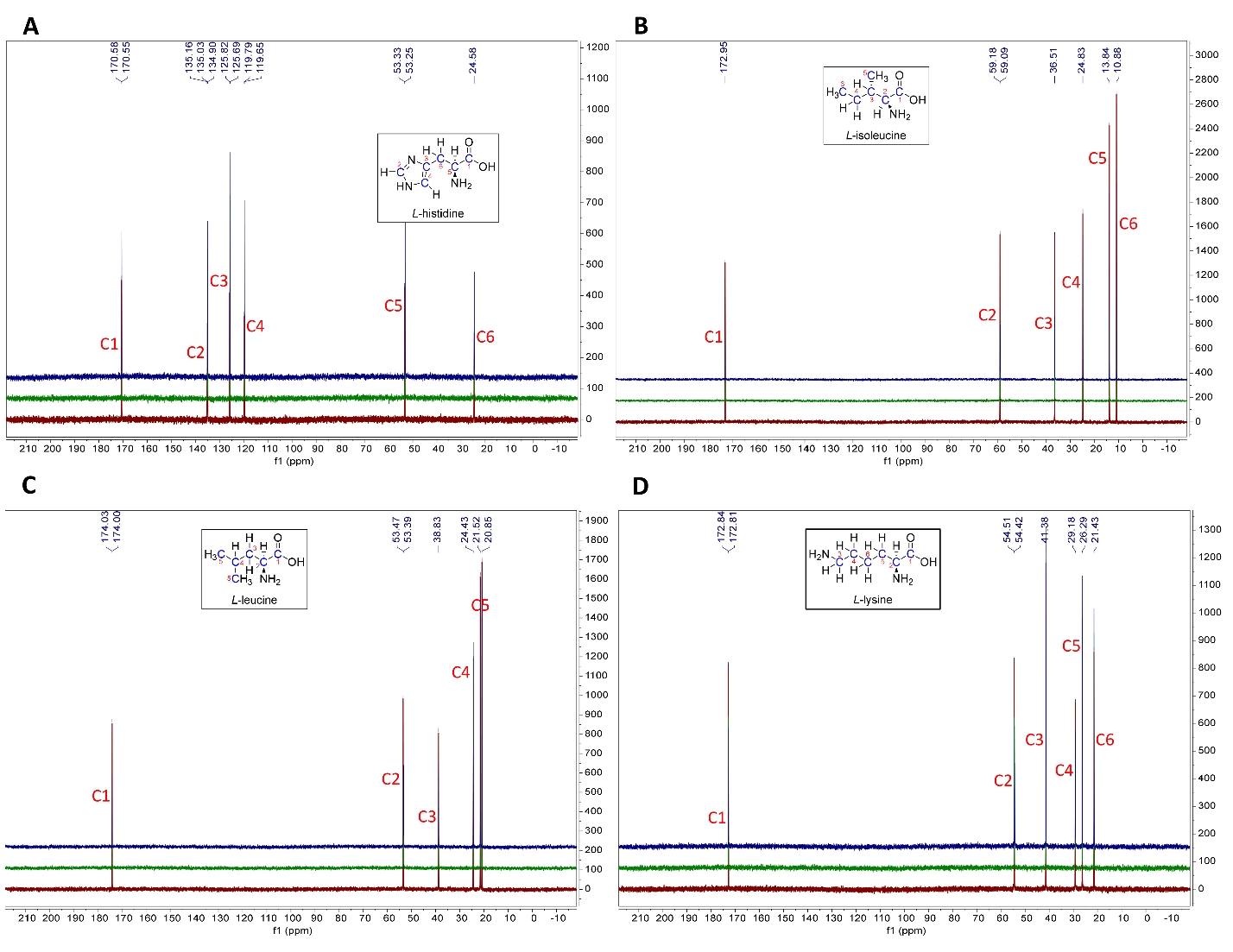
**Figure S9.** The comparison of the ^13^C NMR spectra for biogenic amino acids: histidine, isoleucine, leucine, lysine in concentrated sulfuric acid (98% D_2_SO_4_ and 2% D_2_O, by weight), at room temperature, collected after 12-18h incubation (red spectra), 5-8 day incubation (green spectra) to spectra collected after four week incubation (blue spectra). **A)** The ^13^C NMR of histidine. **B)** The ^13^C NMR of isoleucine. **C)** The ^13^C NMR of leucine. **D)** The ^13^C NMR of lysine. For consistency the C atoms on the illustrative structures of amino acids have been numbered accordingly to the convention used in (Saito et al., 2006).


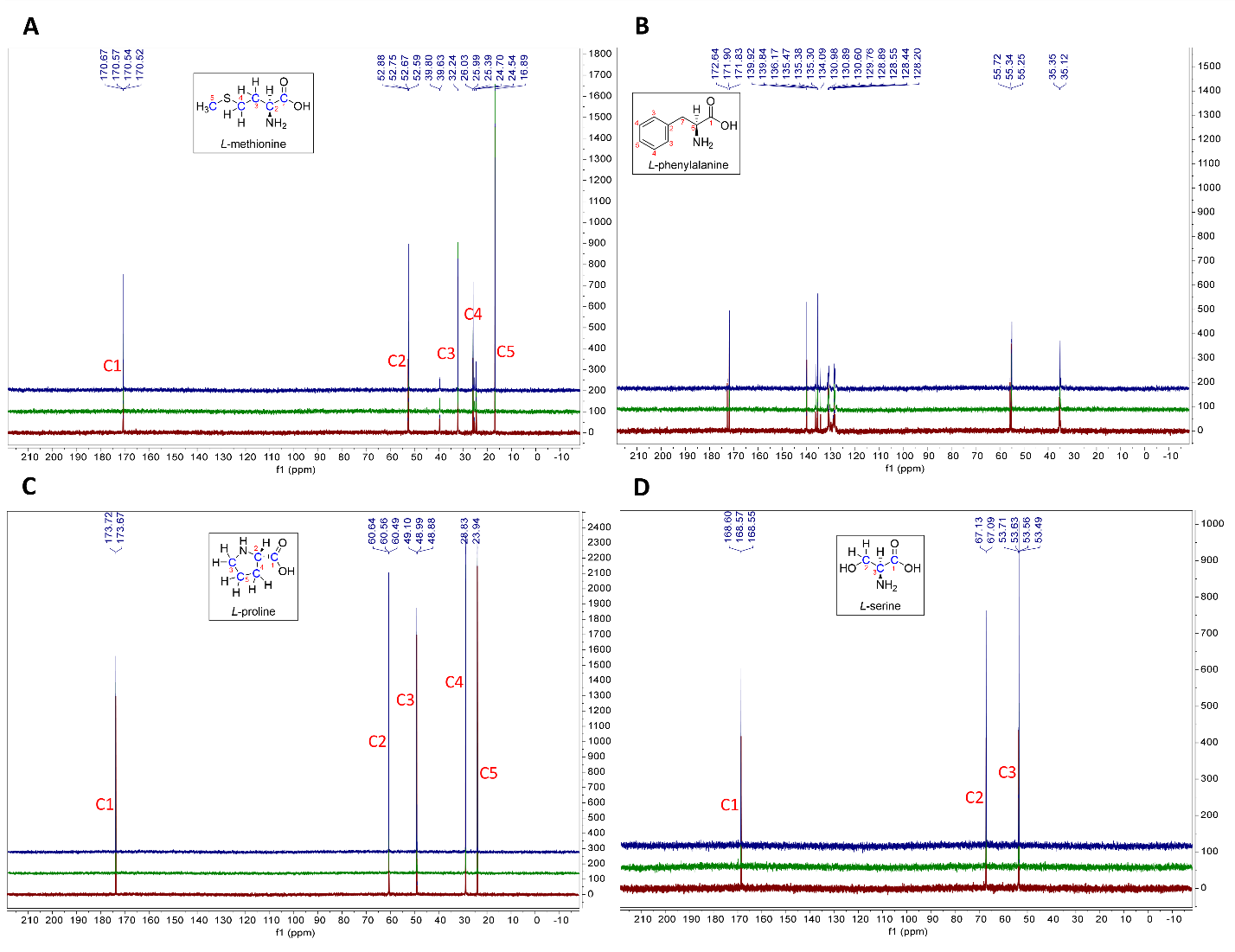
**Figure S10.** The comparison of the ^13^C NMR spectra for biogenic amino acids: methionine, phenylalanine, proline, serine in concentrated sulfuric acid (98% D_2_SO_4_ and 2% D_2_O, by weight), at room temperature, collected after 12-18h incubation (red spectra), 5-8 day incubation (green spectra) to spectra collected after four week incubation (blue spectra). **A)** The ^13^C NMR of methionine. Note that methionine is likely demethylated in concentrated sulfuric acid, which could further promote its reactivity. **B)** The ^13^C NMR of phenylalanine. Phenylalanine aromatic ring is likely sulfonated that could lead to further reactivity in concentrated sulfuric acid. Due to the reactivity of the amino acid side chain in concentrated sulfuric acid C atoms have not been assigned. **C)** The ^13^C NMR of proline. **D)** The ^13^C NMR of serine. Note that the serine side chain hydroxyl (OH) group is sulfated in concentrated sulfuric acid. For consistency the C atoms on the illustrative structures of amino acids have been numbered accordingly to the convention used in (Saito et al., 2006).


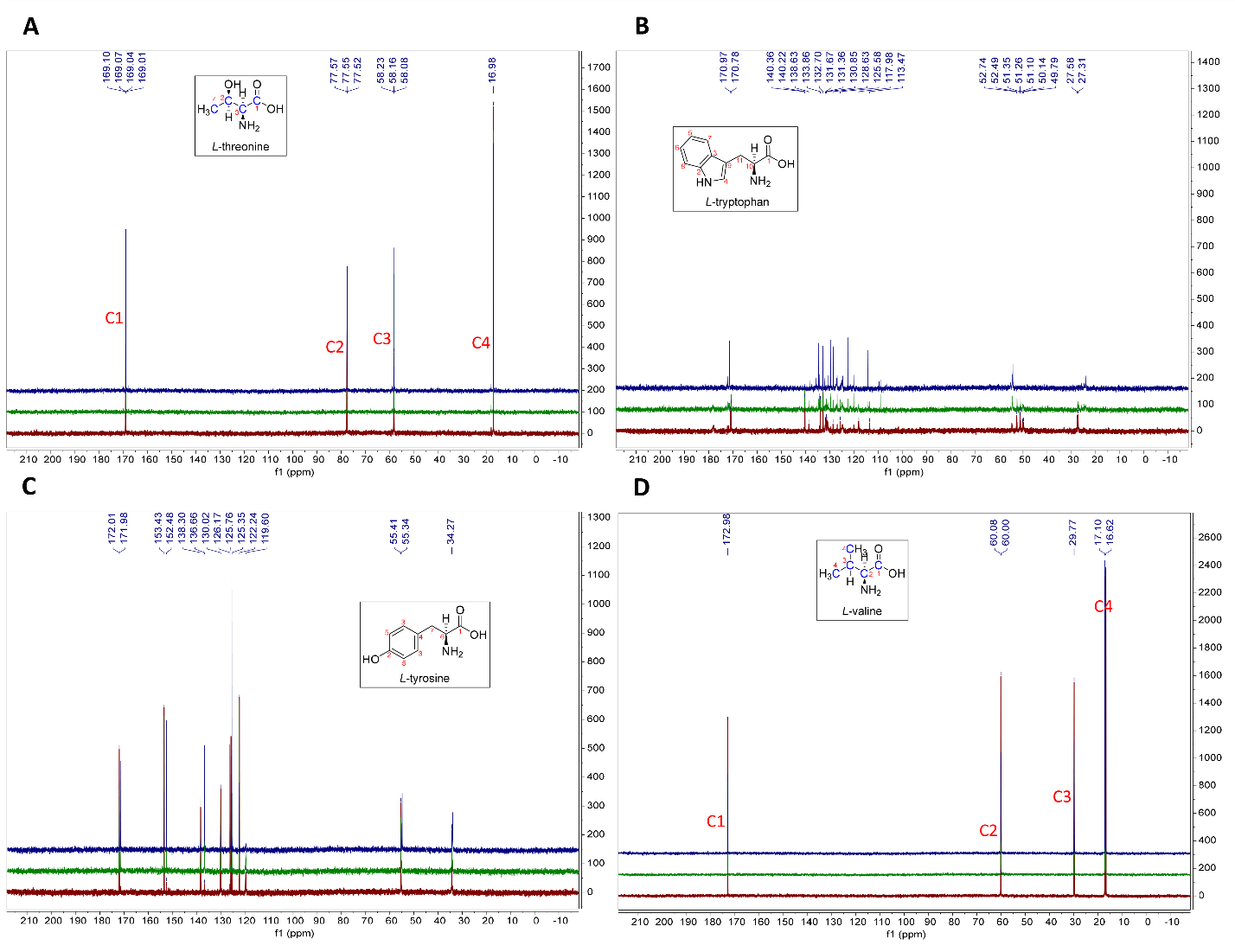
**Figure S11.** The comparison of the ^13^C NMR spectra for biogenic amino acids: threonine, tryptophan, tyrosine, valine in concentrated sulfuric acid (98% D_2_SO_4_ and 2% D_2_O, by weight), at room temperature, collected after 12-18h incubation (red spectra), 5-8 day incubation (green spectra) to spectra collected after four week incubation (blue spectra). **A)** The ^13^C NMR of threonine. Note that threonine side chain hydroxyl (OH) group is likely sulfated in concentrated sulfuric acid. **B)** The ^13^C NMR of tryptophan. Due to the reactivity of the amino acid in concentrated sulfuric acid no C atoms have been assigned. **C)** The ^13^C NMR of tyrosine. Tyrosine likely gets sulfonated and/or sulfated in 98% w/w concentrated sulfuric acid. This modification could lead to its further reactivity. Due to the reactivity of the amino acid side chain in concentrated sulfuric acid C atoms have not been assigned. **D)** The ^13^C NMR of valine. For consistency the C atoms on the illustrative structures of amino acids have been numbered accordingly to the convention used in (Saito et al., 2006).


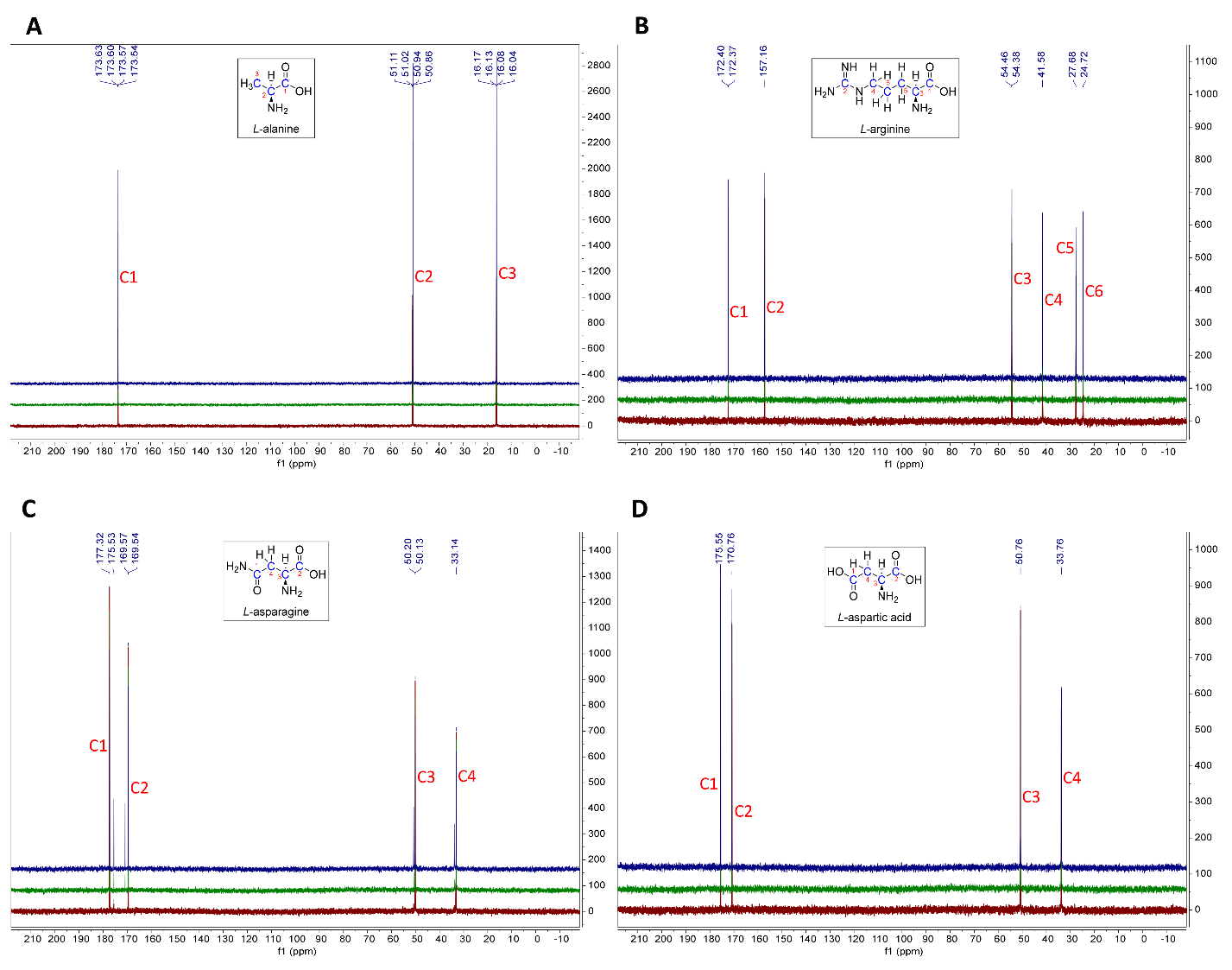
**Figure S12.** The comparison of the ^13^C NMR spectra for biogenic amino acids: alanine, arginine, asparagine, aspartic acid in concentrated sulfuric acid (81% D_2_SO_4_ and 19% D_2_O, by weight), at room temperature, collected after 12-18h incubation (red spectra), 5-8 day incubation (green spectra) to spectra collected after four week incubation (blue spectra). **A)** The ^13^C NMR of alanine. **B)** The ^13^C NMR of arginine. **C)** The ^13^C NMR of asparagine. **D)** The ^13^C NMR of aspartic acid. For consistency the C atoms on the illustrative structures of amino acids have been numbered accordingly to the convention used in (Saito et al., 2006).


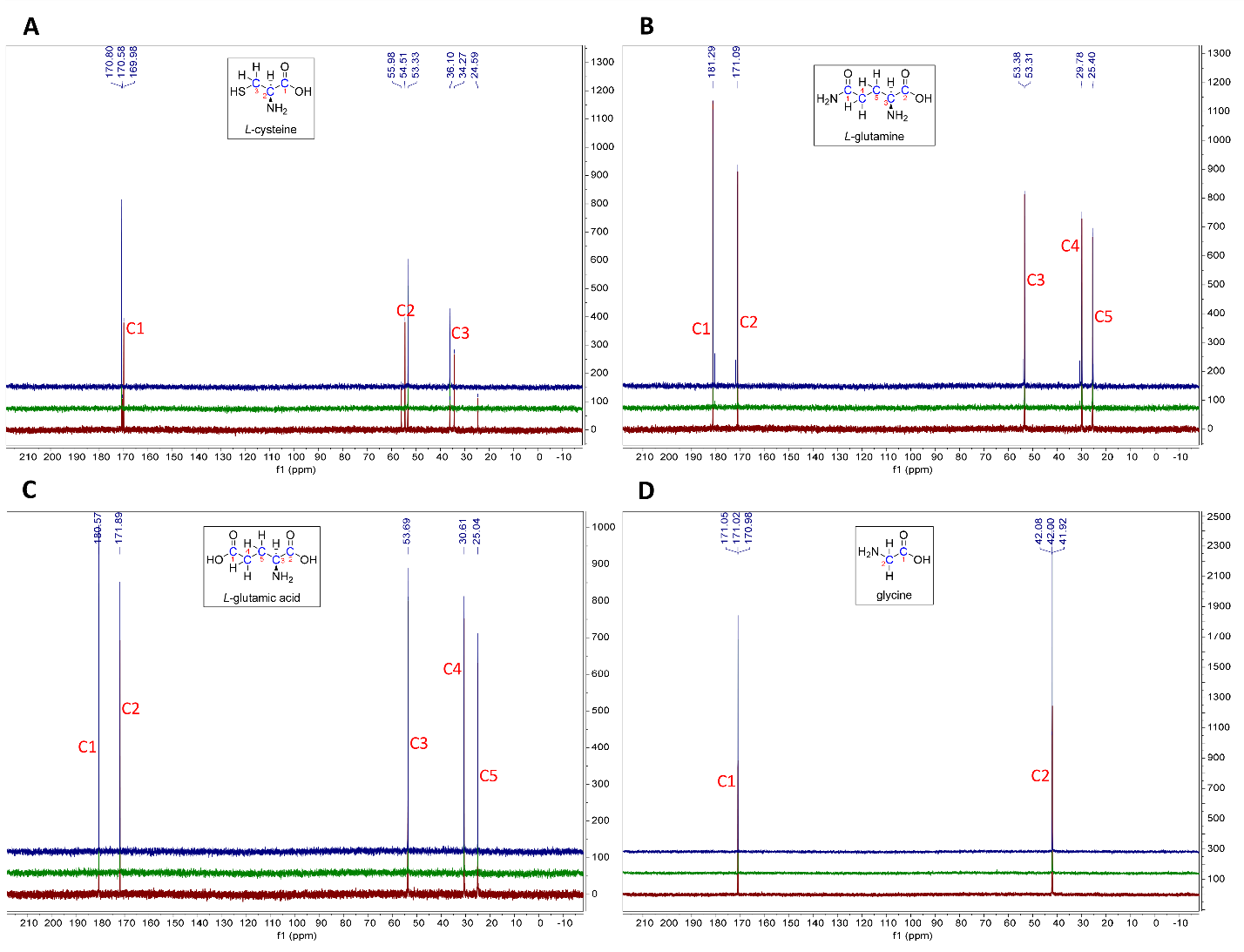
**Figure S13.** The comparison of the ^13^C NMR spectra for biogenic amino acids: cysteine, glutamine, glutamic acid, glycine in concentrated sulfuric acid (81% D_2_SO_4_ and 19% D_2_O, by weight), at room temperature, collected after 12-18h incubation (red spectra), 5-8 day incubation (green spectra) to spectra collected after four week incubation (blue spectra). **A)** The ^13^C NMR of cysteine. Note that cysteine is sulfated in concentrated sulfuric acid. **B)** The ^13^C NMR of glutamine. **C)** The ^13^C NMR of glutamic acid. **D)** The ^13^C NMR of glycine. For consistency the C atoms on the illustrative structures of amino acids have been numbered accordingly to the convention used in (Saito et al., 2006).


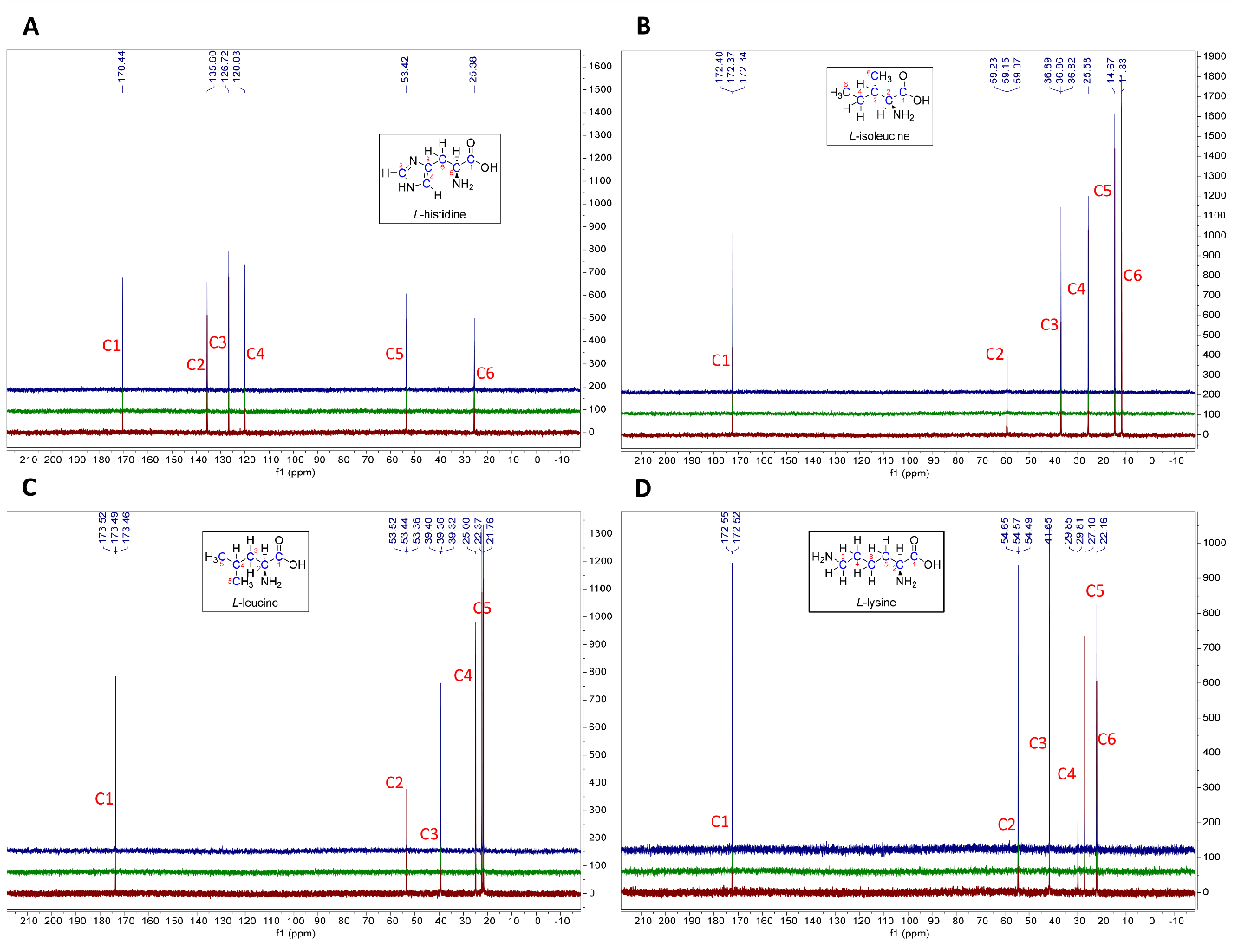
**Figure S14.** The comparison of the ^13^C NMR spectra for biogenic amino acids: histidine, isoleucine, leucine, lysine in concentrated sulfuric acid (81% D_2_SO_4_ and 19% D_2_O, by weight), at room temperature, collected after 12-18h incubation (red spectra), 5-8 day incubation (green spectra) to spectra collected after four week incubation (blue spectra). **A)** The ^13^C NMR of histidine. **B)** The ^13^C NMR of isoleucine. **C)** The ^13^C NMR of leucine. **D)** The ^13^C NMR of lysine. For consistency the C atoms on the illustrative structures of amino acids have been numbered accordingly to the convention used in (Saito et al., 2006).


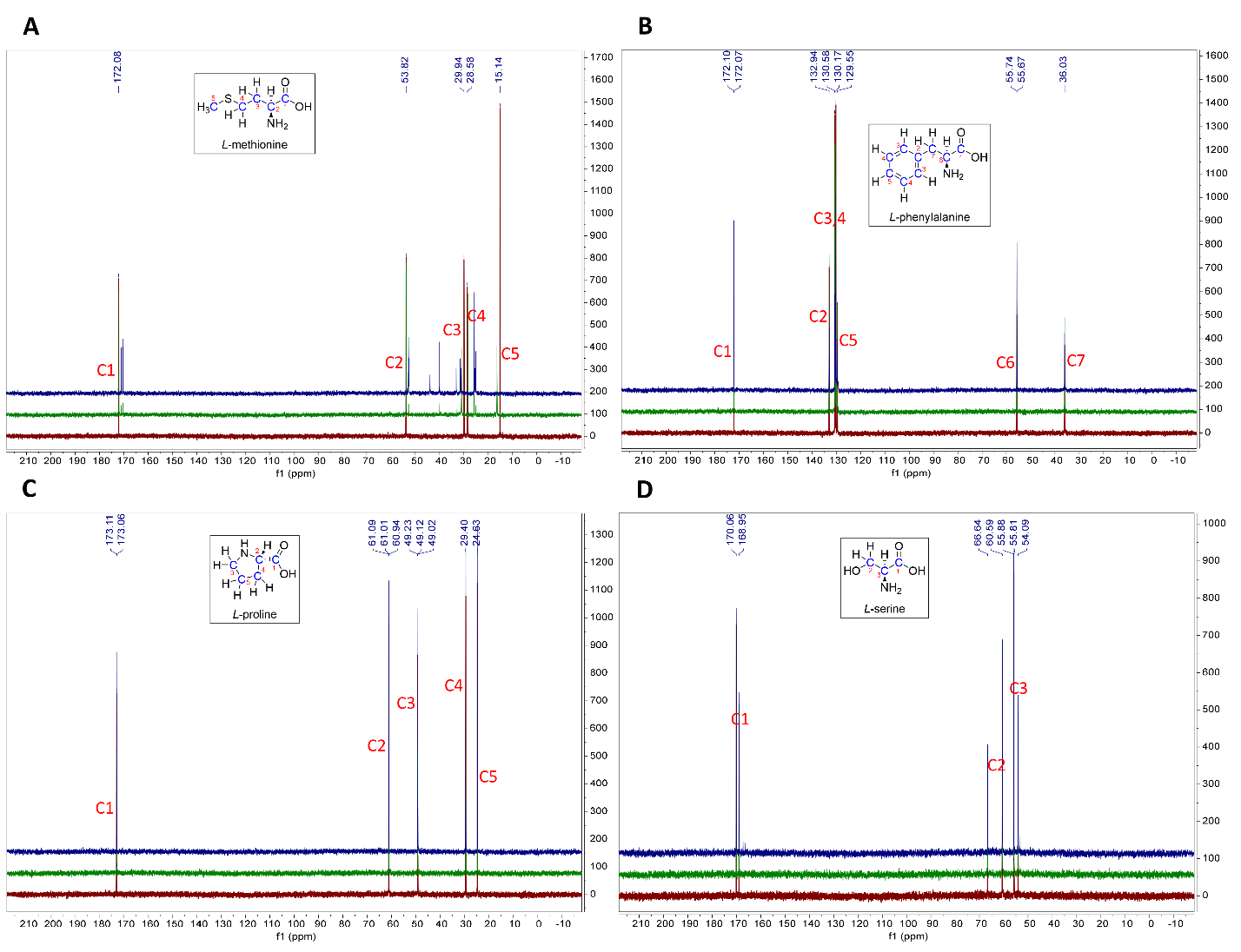
**Figure S15.** The comparison of the ^13^C NMR spectra for biogenic amino acids: methionine, phenylalanine, proline, serine in concentrated sulfuric acid (81% D_2_SO_4_ and 19% D_2_O, by weight), at room temperature, collected after 12-18h incubation (red spectra), 5-8day incubation (green spectra) to spectra collected after four week incubation (blue spectra). **A)** The ^13^C NMR of methionine. Note that methionine is likely demethylated in concentrated sulfuric acid, which could further promote its reactivity. **B)** The ^13^C NMR of phenylalanine. **C)** The ^13^C NMR of proline. **D)** The ^13^C NMR of serine. Note that the serine side chain hydroxyl (OH) group is sulfated in concentrated sulfuric acid. For consistency the C atoms on the illustrative structures of amino acids have been numbered accordingly to the convention used in (Saito et al., 2006).


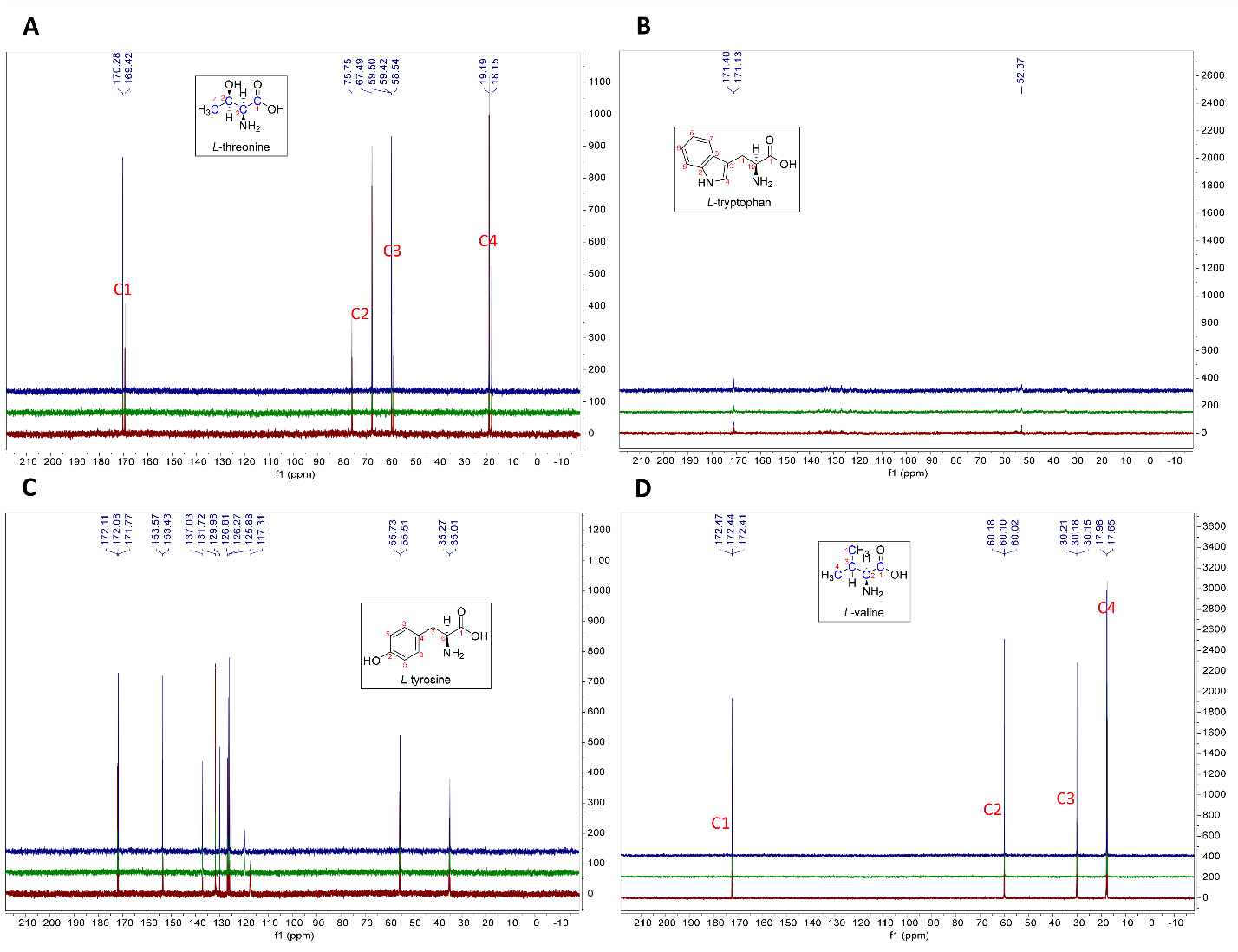
 **Figure S16.** The comparison of the ^13^C NMR spectra for biogenic amino acids: threonine, tryptophan, tyrosine, valine in concentrated sulfuric acid (81% D_2_SO_4_ and 19% D_2_O, by weight), at room temperature, collected after 12-18h incubation (red spectra), 5-8 day incubation (green spectra) to spectra collected after four week incubation (blue spectra). **A)** The ^13^C NMR of threonine. Note that threonine side chain hydroxyl (OH) group is likely sulfated in concentrated sulfuric acid. **B)** The ^13^C NMR of tryptophan. Due to the reactivity of the amino acid in concentrated sulfuric acid no C atoms have been assigned. **C)** The ^13^C NMR of tyrosine. Tyrosine could get sulfated and or sulfonated in 81% w/w sulfuric acid. Due to the reactivity of the amino acid side chain in concentrated sulfuric acid C atoms have not been assigned. **D)** The ^13^C NMR of valine. For consistency the C atoms on the illustrative structures of amino acids have been numbered accordingly to the convention used in (Saito et al., 2006).

**
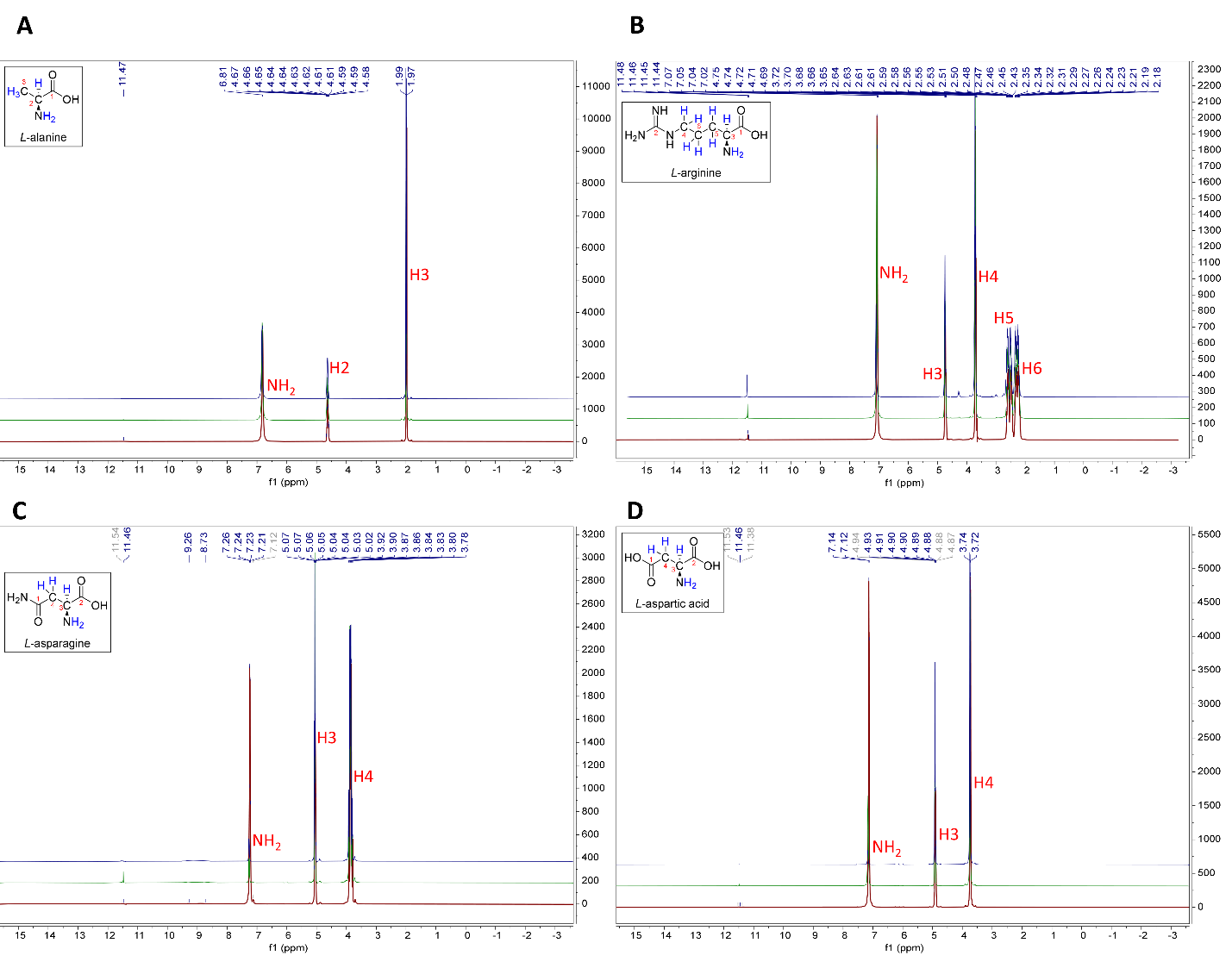
Figure S17.** The comparison of the ^1^H NMR spectra for biogenic amino acids: alanine, arginine, asparagine, aspartic acid in concentrated sulfuric acid (98% D_2_SO_4_ and 2% D_2_O, by weight), at room temperature, collected after 12-18h incubation (red spectra), 5-8 day incubation (green spectra) to spectra collected after four week incubation (blue spectra). The solvent signal is suppressed for clarity. **A)** The ^1^H NMR of alanine. **B)** The ^1^H NMR of arginine. **C)** The ^1^H NMR of asparagine. **D)** The ^1^H NMR of aspartic acid. For consistency the C atoms on the illustrative structures of amino acids have been numbered accordingly to the convention used in (Saito et al., 2006).


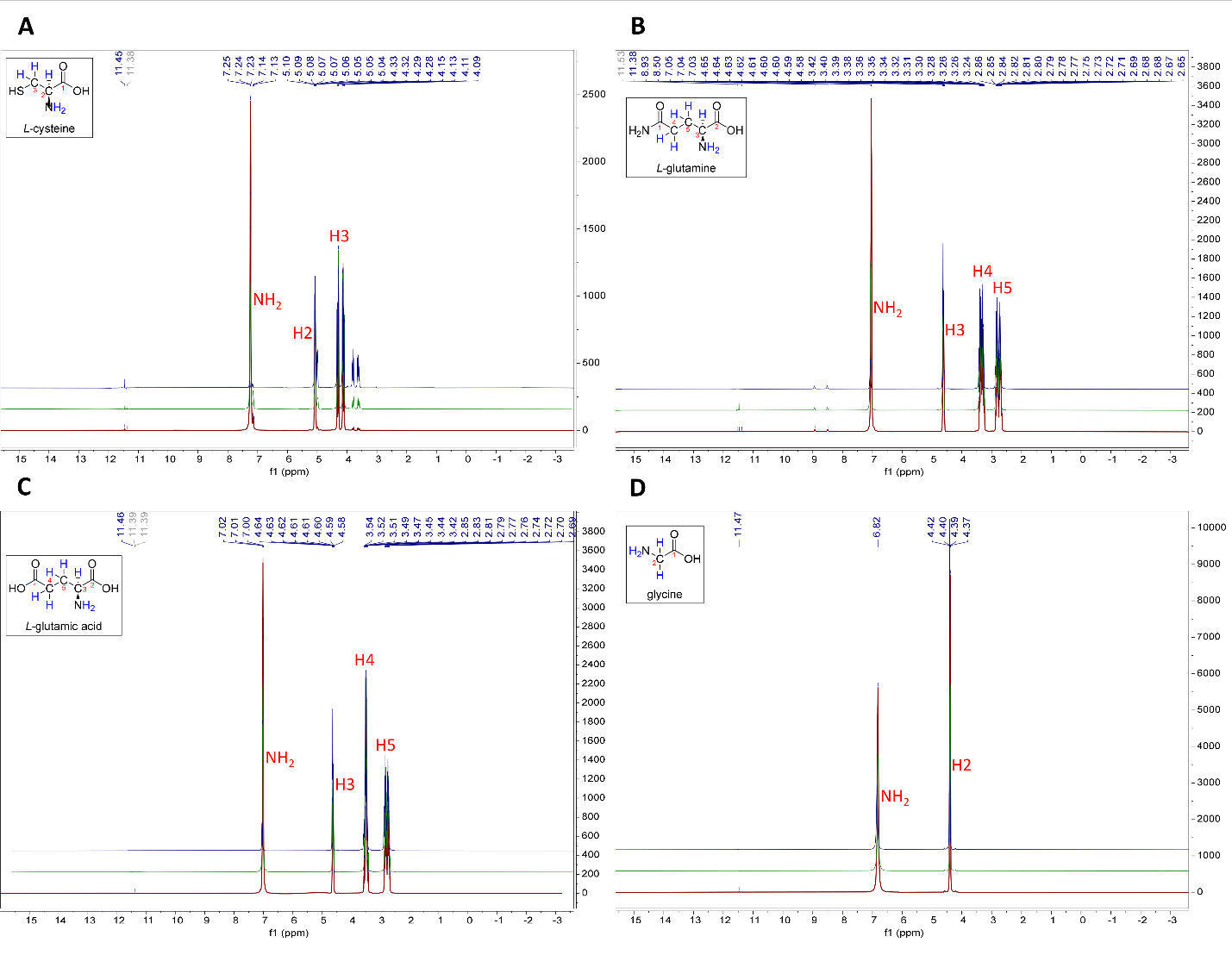
**Figure S18.** The comparison of the ^1^H NMR spectra for biogenic amino acids: cysteine, glutamine, glutamic acid, glycine in concentrated sulfuric acid (98% D_2_SO_4_ and 2% D_2_O, by weight), at room temperature, collected after 12-18h incubation (red spectra), 5-8 day incubation (green spectra) to spectra collected after four week incubation (blue spectra). The solvent signal is suppressed for clarity. **A)** The ^1^H NMR of cysteine. Note that cysteine is sulfated in concentrated sulfuric acid. **B)** The ^1^H NMR of glutamine. **C)** The ^1^H NMR of glutamic acid. **D)** The ^1^H NMR of glycine. For consistency the C atoms on the illustrative structures of amino acids have been numbered accordingly to the convention used in (Saito et al., 2006).


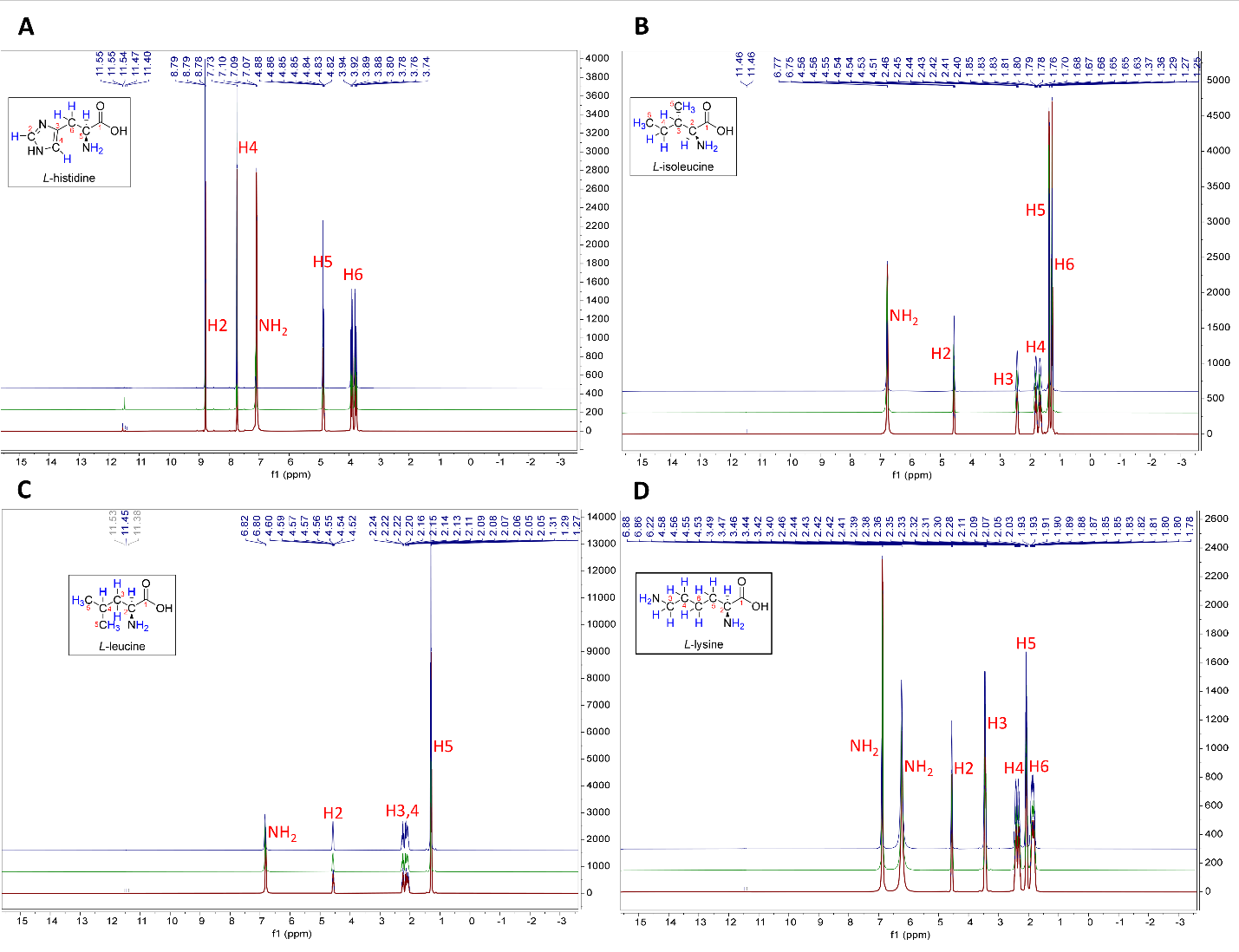
**Figure S19.** The comparison of the ^1^H NMR spectra for biogenic amino acids: histidine, isoleucine, leucine, lysine in concentrated sulfuric acid (98% D_2_SO_4_ and 2% D_2_O, by weight), at room temperature, collected after 12-18h incubation (red spectra), 5-8 day incubation (green spectra) to spectra collected after four week incubation (blue spectra). The solvent signal is suppressed for clarity. **A)** The ^1^H NMR of histidine. **B)** The ^1^H NMR of isoleucine. **C)** The ^1^H NMR of leucine. **D)** The ^1^H NMR of lysine. For consistency the C atoms on the illustrative structures of amino acids have been numbered accordingly to the convention used in (Saito et al., 2006).


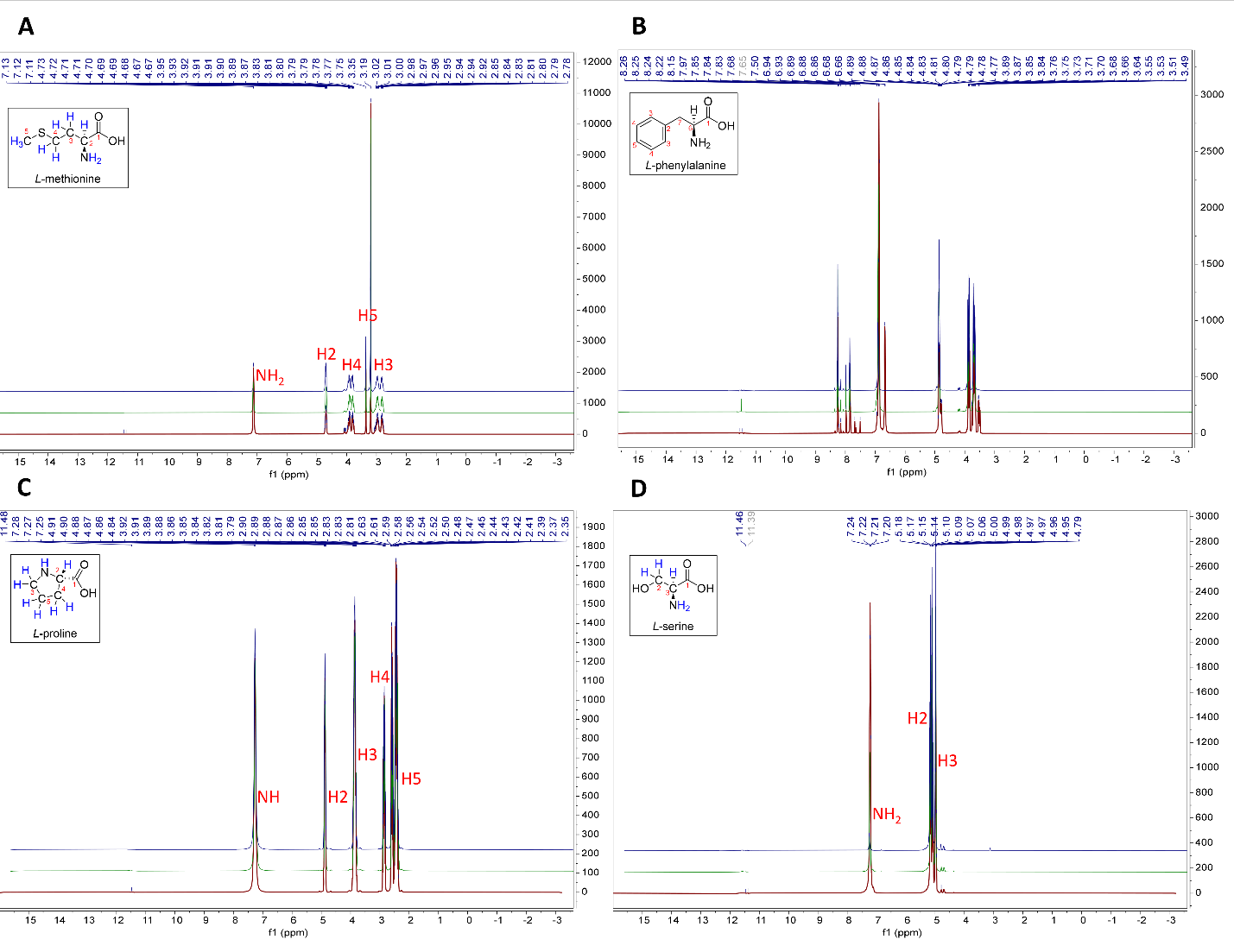
**Figure S20.** The comparison of the ^1^H NMR spectra for biogenic amino acids: methionine, phenylalanine, proline, serine in concentrated sulfuric acid (98% D_2_SO_4_ and 2% D_2_O, by weight), at room temperature, collected after 12-18h incubation (red spectra), 5-8 day incubation (green spectra) to spectra collected after four week incubation (blue spectra). The solvent signal is suppressed for clarity. **A)** The ^1^H NMR of methionine. Note that methionine is likely demethylated in concentrated sulfuric acid, which could further promote its reactivity. The H atoms in methionine have been assigned by comparison to ^1^H NMR spectra in D_2_O (Saito et al., 2006). **B)** The ^1^H NMR of phenylalanine. Phenylalanine aromatic ring is likely sulfonated in 98% w/w sulfuric acid. Due to the reactivity of the amino acid side chain in concentrated sulfuric acid H atoms have not been assigned. **C)** The ^1^H NMR of proline. **D)** The ^1^H NMR of serine. Note that serine side chain hydroxyl (OH) group is sulfated in concentrated sulfuric acid. For consistency the C atoms on the illustrative structures of amino acids have been numbered accordingly to the convention used in (Saito et al., 2006).


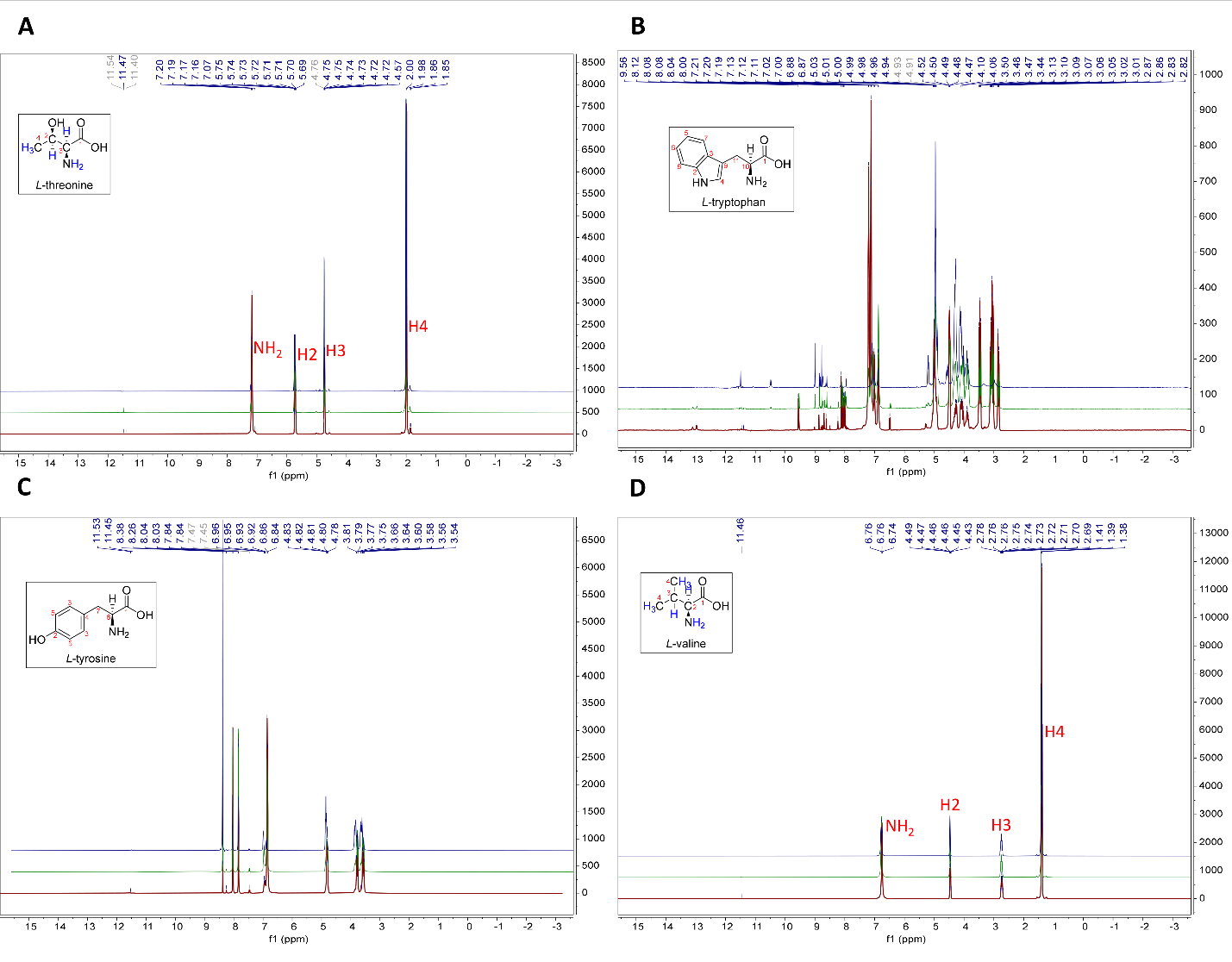
**Figure S21.** The comparison of the ^1^H NMR spectra for biogenic amino acids: threonine, tryptophan, tyrosine, valine in concentrated sulfuric acid (98% D_2_SO_4_ and 2% D_2_O, by weight), at room temperature, collected after 12-18h incubation (red spectra), 5-8 day incubation (green spectra) to spectra collected after four week incubation (blue spectra). The solvent signal is suppressed for clarity. **A)** The ^1^H NMR of threonine. Note that threonine side chain hydroxyl (OH) group is likely sulfated in concentrated sulfuric acid. **B)** The ^1^H NMR of tryptophan. Due to the reactivity of the amino acid in concentrated sulfuric acid no H atoms have been assigned. **C)** The ^1^H NMR of tyrosine. Tyrosine likely gets sulfated and/or sulfonated in 98% w/w sulfuric acid. This modification could lead to its further reactivity. Due to the reactivity of the amino acid side chain in concentrated sulfuric acid H atoms have not been assigned. **D)** The ^1^H NMR of valine. For consistency the C atoms on the illustrative structures of amino acids have been numbered accordingly to the convention used in (Saito et al., 2006).

**
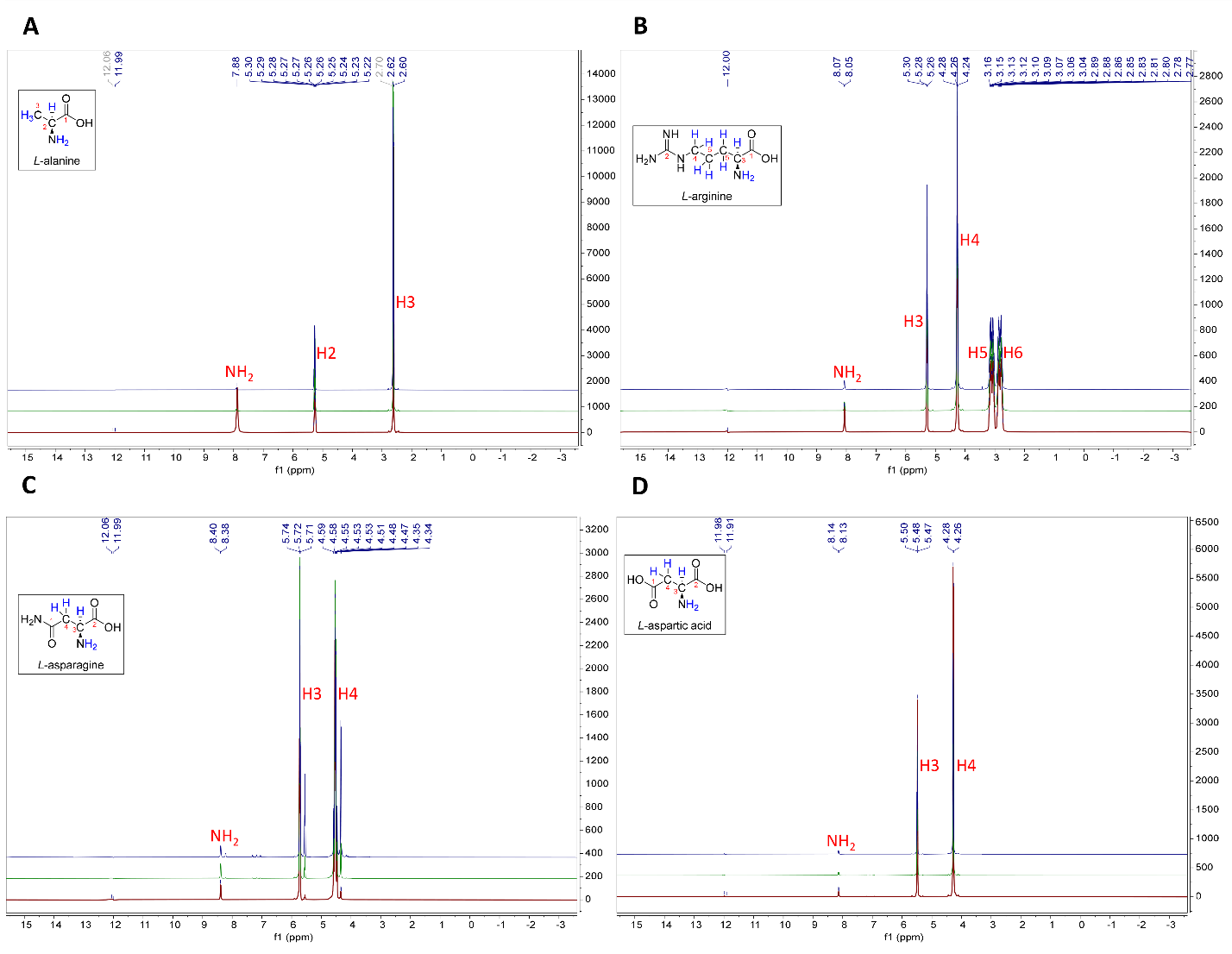
Figure S22.** The comparison of the ^1^H NMR spectra for biogenic amino acids: alanine, arginine, asparagine, aspartic acid in concentrated sulfuric acid (81% D_2_SO_4_ and 19% D_2_O, by weight), at room temperature, collected after 12-18h incubation (red spectra), 5-8 day incubation (green spectra) to spectra collected after four week incubation (blue spectra). The solvent signal is suppressed for clarity. **A)** The ^1^H NMR of alanine. **B)** The ^1^H NMR of arginine. **C)** The ^1^H NMR of asparagine. **D)** The ^1^H NMR of aspartic acid. For consistency the C atoms on the illustrative structures of amino acids have been numbered accordingly to the convention used in (Saito et al., 2006).


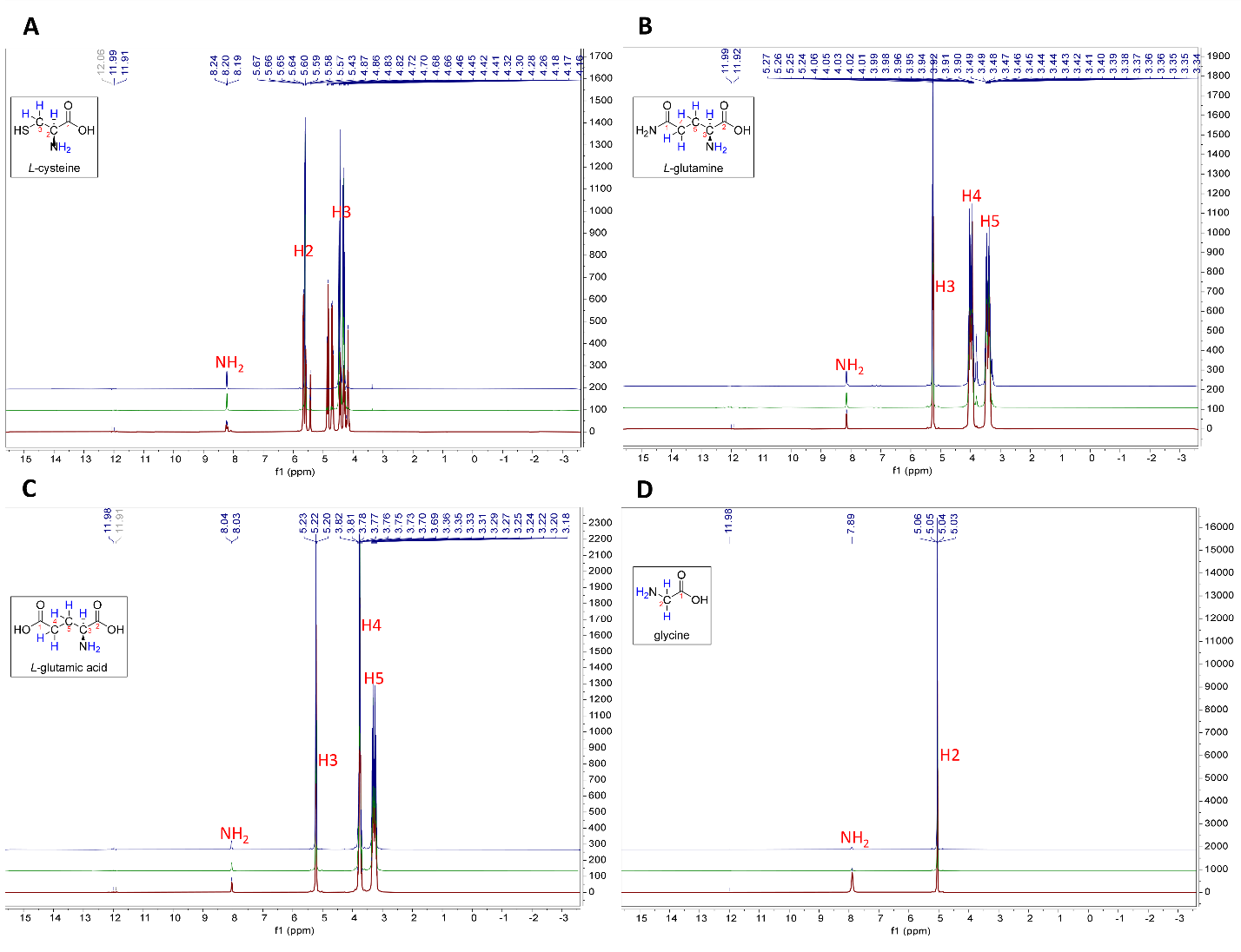
**Figure S23.** The comparison of the ^1^H NMR spectra for biogenic amino acids: cysteine, glutamine, glutamic acid, glycine in concentrated sulfuric acid (81% D_2_SO_4_ and 19% D_2_O, by weight), at room temperature, collected after 12-18h incubation (red spectra), 5-8 day incubation (green spectra) to spectra collected after four week incubation (blue spectra). The solvent signal is suppressed for clarity. **A)** The ^1^H NMR of cysteine. Note that cysteine is sulfated in concentrated sulfuric acid. **B)** The ^1^H NMR of glutamine. **C)** The ^1^H NMR of glutamic acid. **D)** The ^1^H NMR of glycine. For consistency the C atoms on the illustrative structures of amino acids have been numbered accordingly to the convention used in (Saito et al., 2006).


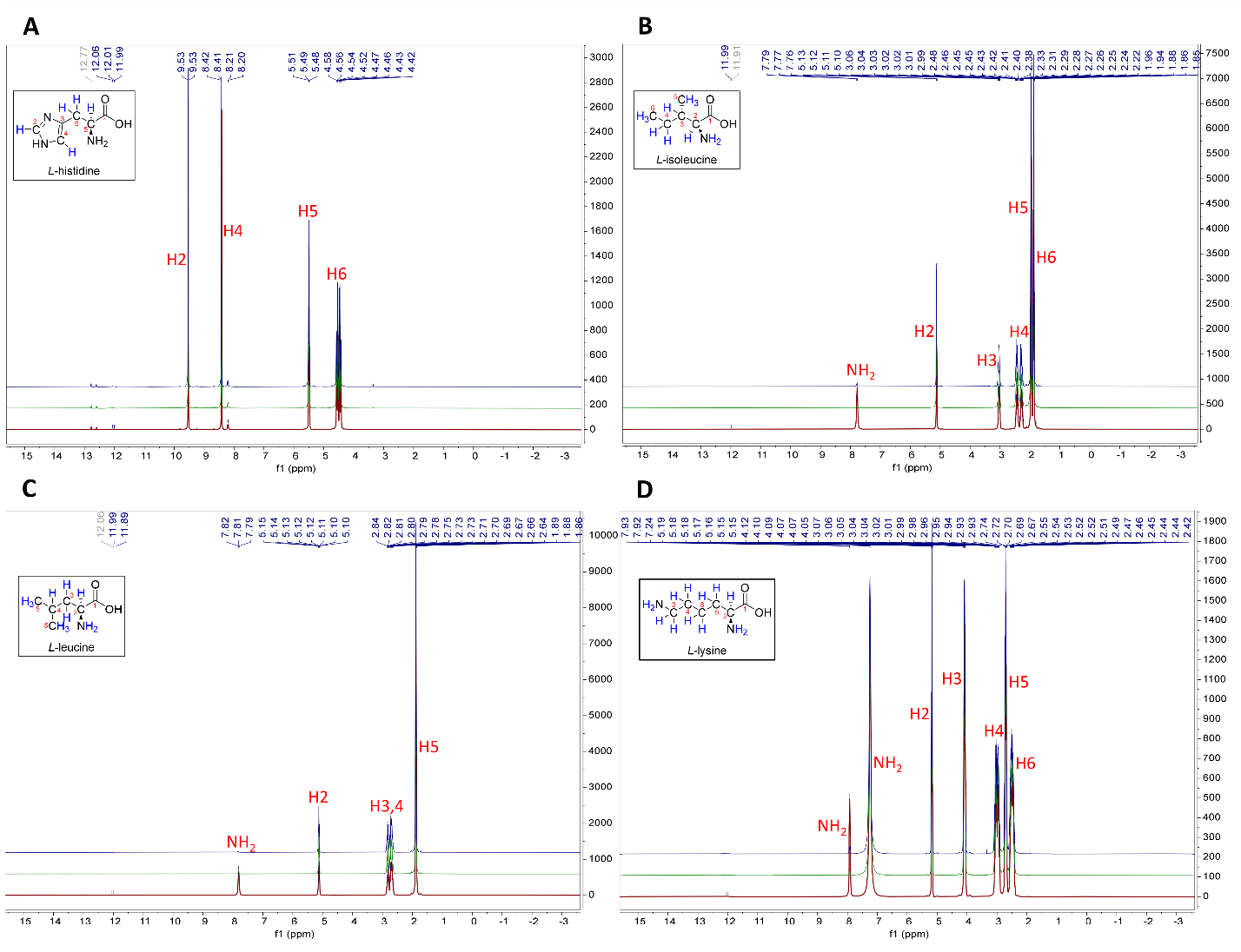
**Figure S24.** The comparison of the ^1^H NMR spectra for biogenic amino acids: histidine, isoleucine, leucine, lysine in concentrated sulfuric acid (81% D_2_SO_4_ and 19% D_2_O, by weight), at room temperature, collected after 12-18h incubation (red spectra), 5-8 day incubation (green spectra) to spectra collected after four week incubation (blue spectra). The solvent signal is suppressed for clarity. **A)** The ^1^H NMR of histidine. **B)** The ^1^H NMR of isoleucine. **C)** The ^1^H NMR of leucine. **D)** The ^1^H NMR of lysine. For consistency the C atoms on the illustrative structures of amino acids have been numbered accordingly to the convention used in (Saito et al., 2006).


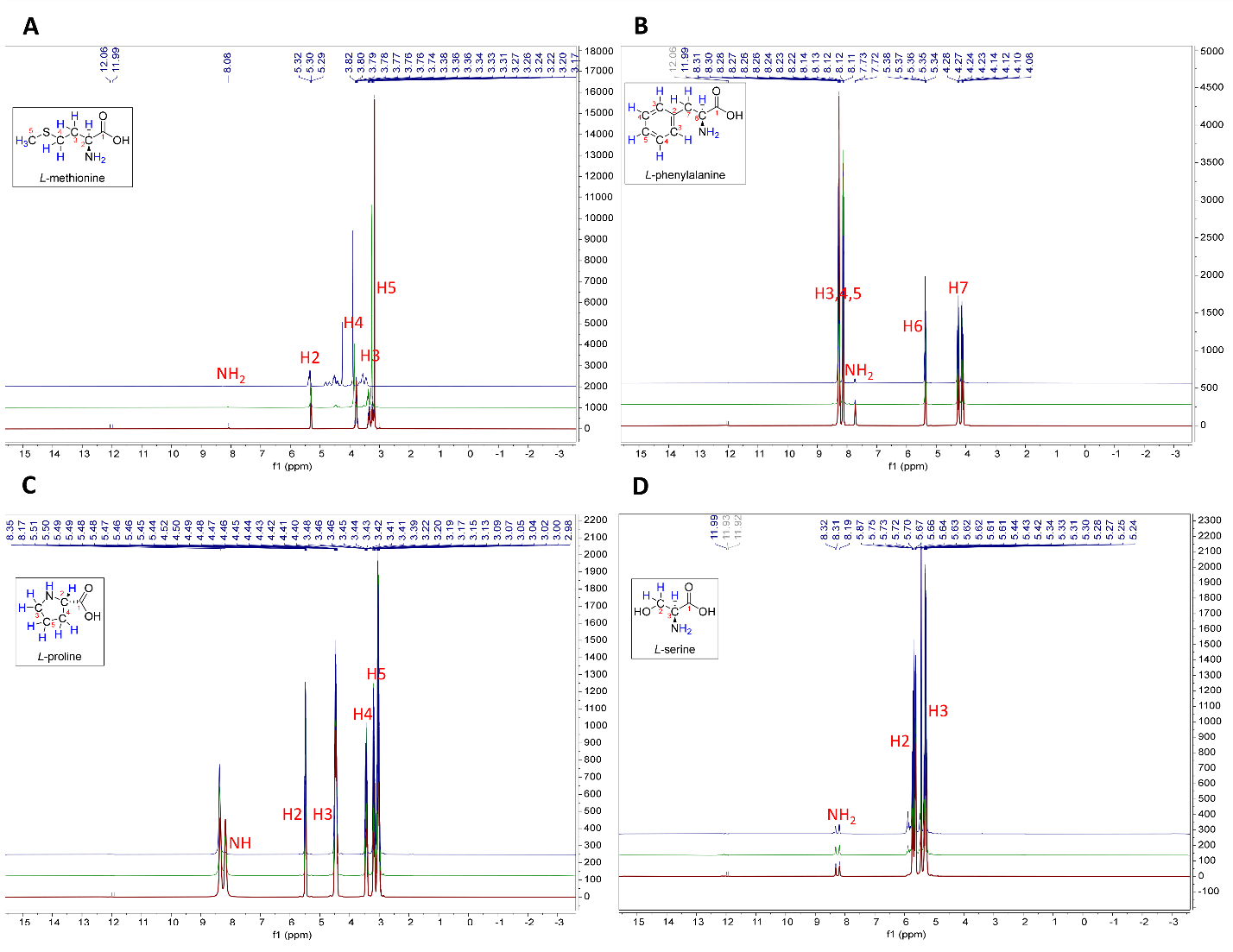
**Figure S25.** The comparison of the ^1^H NMR spectra for biogenic amino acids: methionine, phenylalanine, proline, serine in concentrated sulfuric acid (81% D_2_SO_4_ and 19% D_2_O, by weight), at room temperature, collected after 12-18h incubation (red spectra), 5-8 day incubation (green spectra) to spectra collected after four week incubation (blue spectra). The solvent signal is suppressed for clarity. **A)** The ^1^H NMR of methionine. Note that methionine is likely demethylated in concentrated sulfuric acid, which could further promote its reactivity. The H atoms in methionine have been assigned by comparison to ^1^H NMR spectra in D_2_O (Saito et al., 2006). **B)** The ^1^H NMR of phenylalanine. In contrast to 98% w/w sulfuric acid phenylalanine in 81% w/w sulfuric acid is stable and unreactive for at least four weeks. The H atoms in phenylalanine have been assigned by comparison to ^1^H NMR spectra in D_2_O (Saito et al., 2006). **C)** The ^1^H NMR of proline. **D)** The ^1^H NMR of serine. Note that serine side chain hydroxyl (OH) group is sulfated in concentrated sulfuric acid, although this process is less efficient than in 98% w/w (Figure S5; Figure S6). For consistency the C atoms on the illustrative structures of amino acids have been numbered accordingly to the convention used in (Saito et al., 2006).


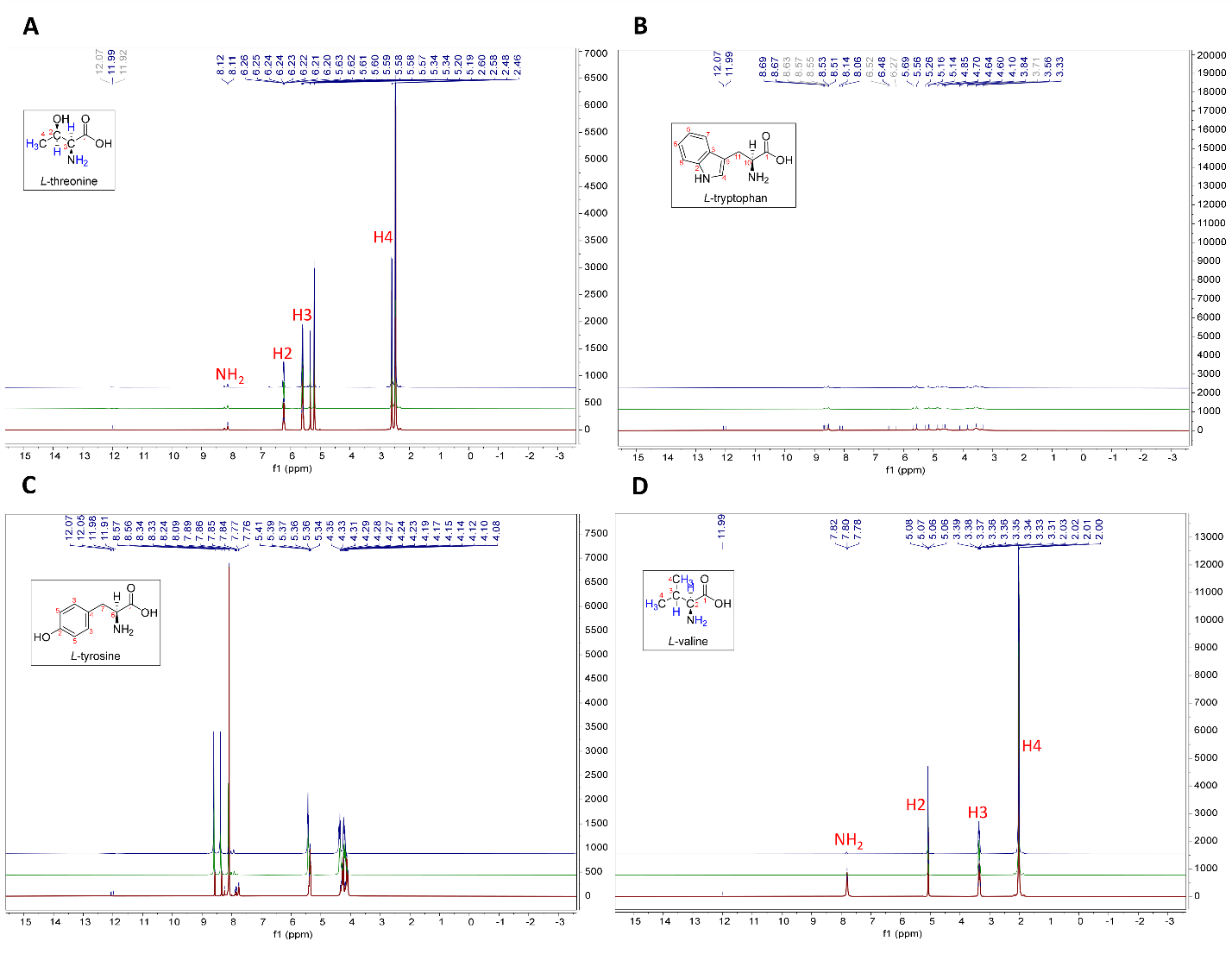
**Figure S26.** The comparison of the ^1^H NMR spectra for biogenic amino acids: threonine, tryptophan, tyrosine, valine in concentrated sulfuric acid (81% D_2_SO_4_ and 19% D_2_O, by weight), at room temperature, collected after 12-18h incubation (red spectra), 5-8 day incubation (green spectra) to spectra collected after four week incubation (blue spectra). The solvent signal is suppressed for clarity. **A)** The ^1^H NMR of threonine. Note that threonine side chain hydroxyl (OH) group is likely sulfated in concentrated sulfuric acid, although this process is less efficient than in 98% w/w (Figure S5; Figure S6). **B)** The ^1^H NMR of tryptophan. Due to the reactivity of the amino acid in concentrated sulfuric acid no H atoms have been assigned. **C)** The ^1^H NMR of tyrosine. Tyrosine likely gets sulfated and/or sulfonated in 81% w/w concentrated sulfuric acid. Due to the reactivity of the amino acid side chain in concentrated sulfuric acid H atoms have not been assigned. **D)** The ^1^H NMR of valine. For consistency the C atoms on the illustrative structures of amino acids have been numbered accordingly to the convention used in (Saito et al., 2006).


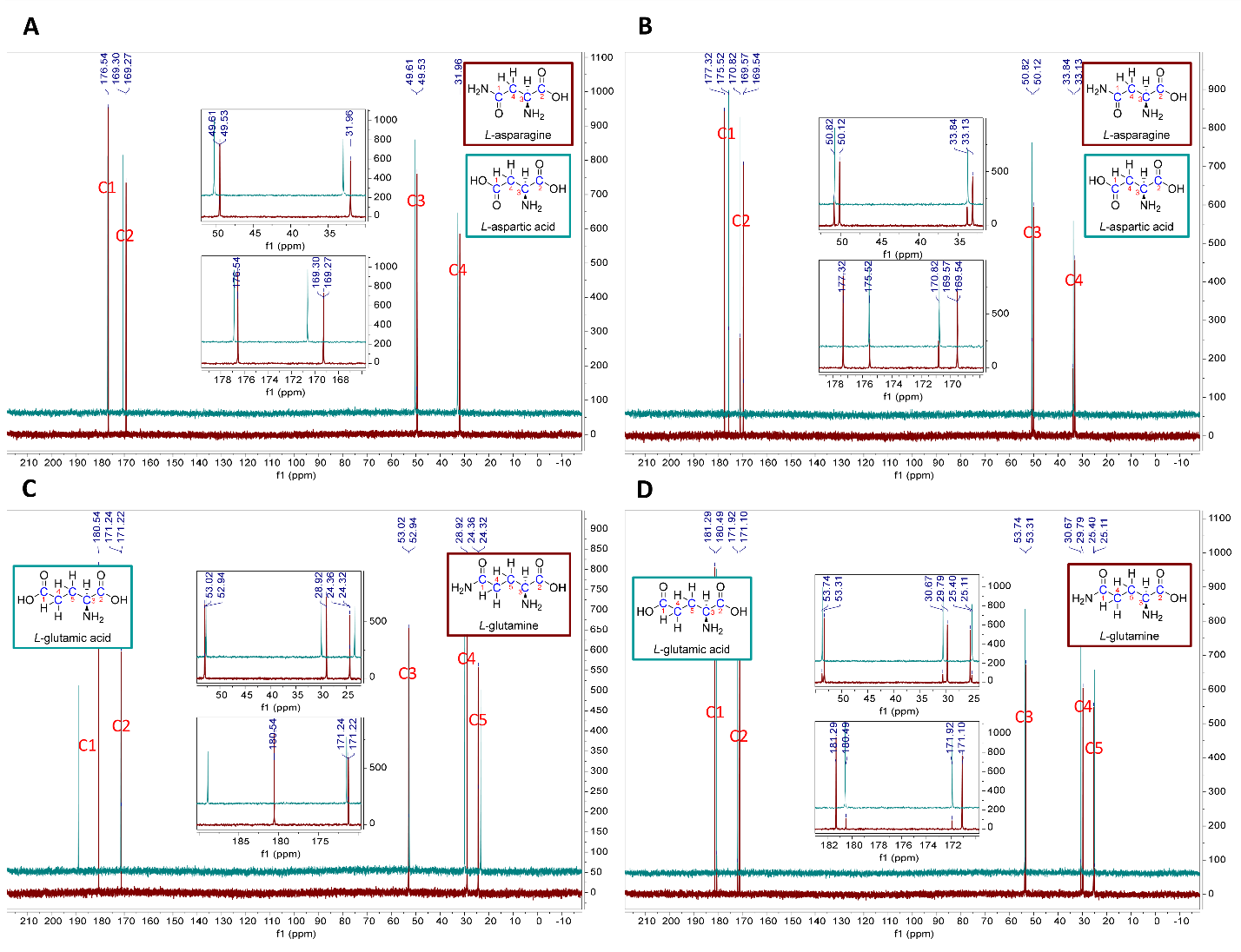
**Figure S27.** Asparagine and glutamine undergo deamidation in 81% w/w concentrated sulfuric acid. **A)** Comparison of asparagine and aspartic acid ^13^C NMR spectra collected after four weeks of incubation in 98% w/w acid. Asparagine does not undergo deamidation reaction and is stable in 98% w/w acid. **B)** Comparison of asparagine and aspartic acid ^13^C NMR spectra collected after four weeks of incubation in 81% w/w acid. Asparagine undergoes deamidation reaction in 81% w/w sulfuric acid and gets gradually converted to aspartic acid. **C)** Comparison of glutamine and glutamic acid ^13^C NMR spectra collected after four weeks of incubation in 98% w/w acid. Glutamine does not undergo deamidation reaction and is stable in 98% w/w acid. **D)** Comparison of glutamine and glutamic acid ^13^C NMR spectra collected after four weeks of incubation in 81% w/w acid. Glutamine undergoes deamidation reaction in 81% w/w sulfuric acid and gets gradually converted to glutamic acid. For consistency the C atoms on the illustrative structures of amino acids have been numbered accordingly to the convention used in (Saito et al., 2006).

**
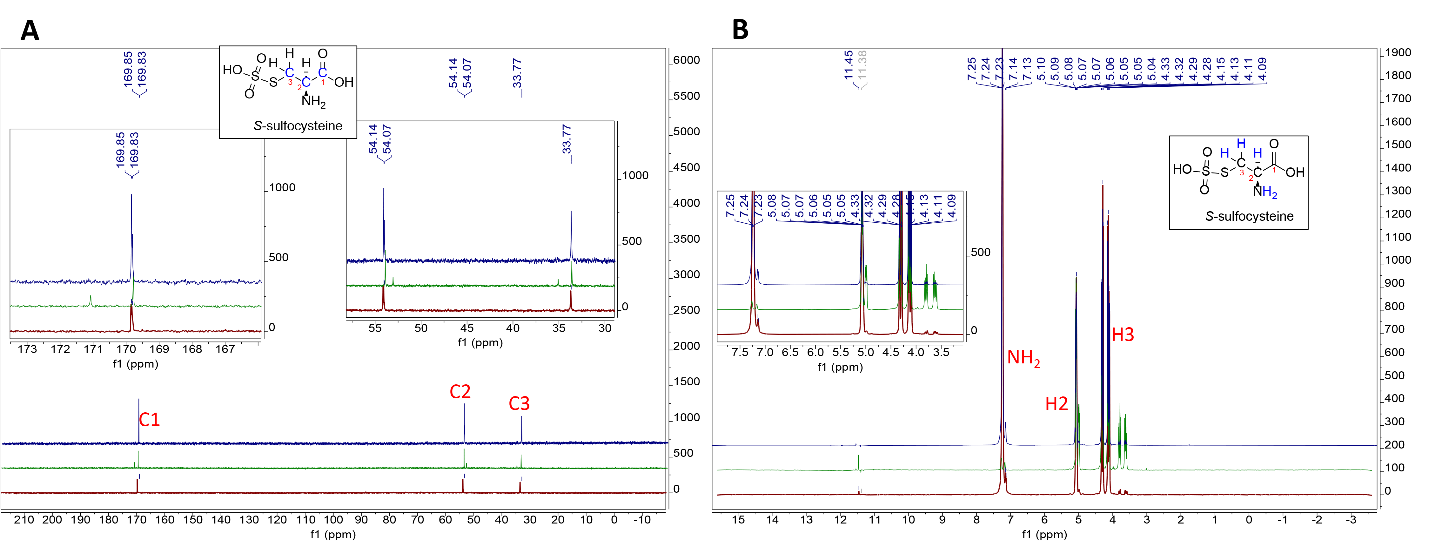
Figure S28.** Comparison of the ^13^C (**A**) and ^1^H (**B**) NMR spectra of cysteine, collected after 12-18h incubation (red spectra) and four-week incubation (green spectra), to spectra of *S*-sulfocysteine (blue spectra) in concentrated sulfuric acid (98% D_2_SO_4_ and 2% D_2_O, by weight), at room temperature. The solvent signal in ^1^H NMR spectra is suppressed for clarity. The *S*-sulfocysteine spectra match the cysteine spectra (red, green spectra) confirming the sulfation of cysteine in concentrated sulfuric acid. For consistency the C atoms on the illustrative structures of amino acids have been numbered accordingly to the convention used in (Saito et al., 2006).

**
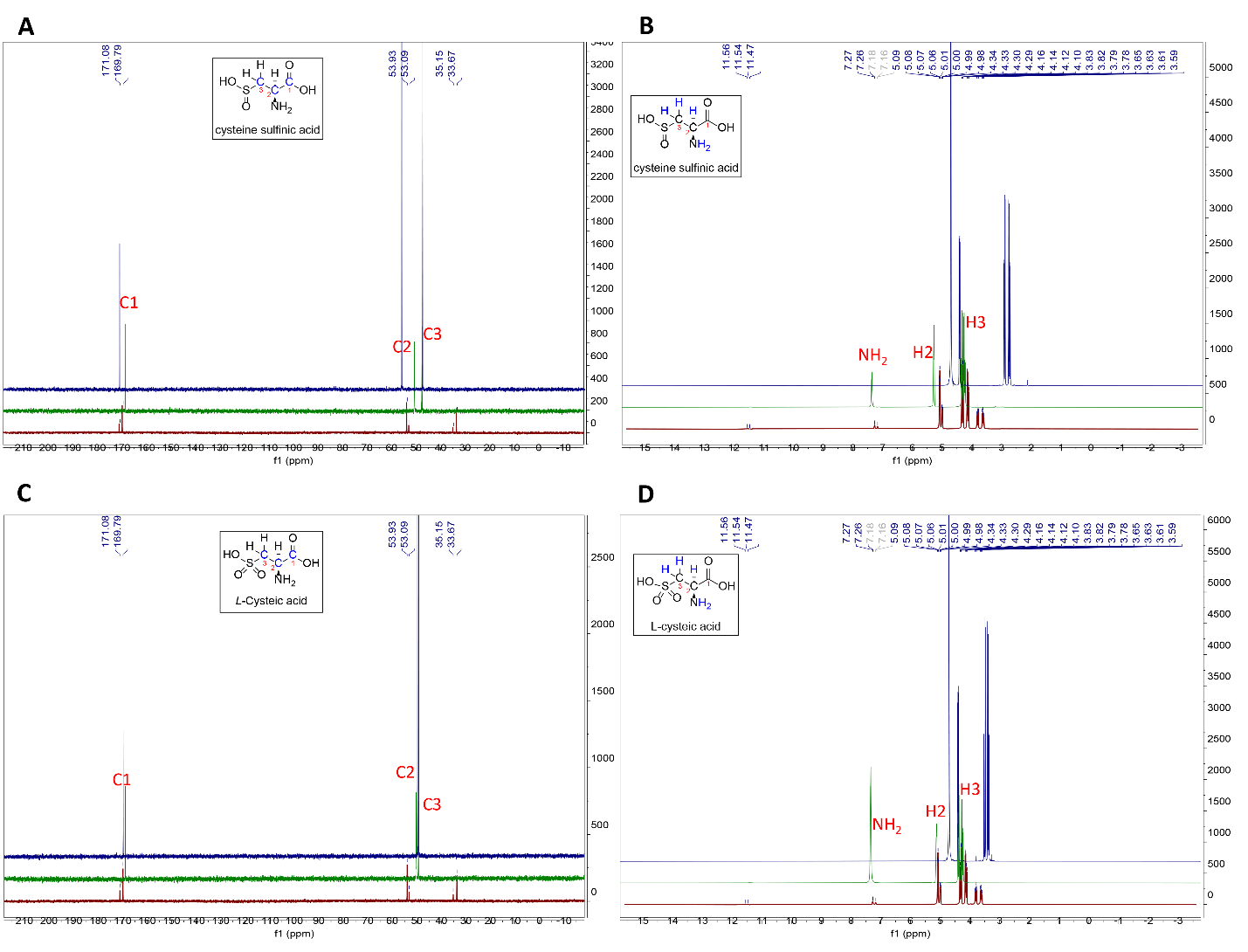
Figure S29.** Comparison of the NMR spectra of cysteine in concentrated sulfuric acid (98% D_2_SO_4_ and 2% D_2_O, by weight) to spectra of cysteine sulfinic acid or cysteic acid. **A)** Comparison of the ^13^C NMR spectra of cysteine, collected after four-week incubation in concentrated sulfuric acid (red spectra), to spectra of cysteine sulfinic acid in concentrated sulfuric acid (green spectra) and D_2_O (blue spectra). **B)** Comparison of the ^1^H NMR spectra of cysteine, collected after four-week incubation (red spectra), to spectra of cysteine sulfinic acid in concentrated sulfuric acid (green spectra) and D_2_O (blue spectra). The solvent signal in ^1^H NMR spectra is suppressed for clarity. **C)** Comparison of the ^13^C NMR spectra of cysteine, collected after four-week incubation (red spectra), to spectra of cysteic acid in concentrated sulfuric acid (green spectra) and D_2_O (blue spectra). **D)** Comparison of the ^1^H NMR spectra of cysteine, collected after four-week incubation (red spectra), to spectra of cysteic acid in concentrated sulfuric acid (green spectra) and D_2_O (blue spectra). The solvent signal in ^1^H NMR spectra is suppressed for clarity. The spectra of cysteine sulfinic acid and cysteic acid do not match the cysteine spectra (red spectra), ruling out the direct oxidation of cysteine in concentrated sulfuric acid. For consistency the C atoms on the illustrative structures of amino acids have been numbered accordingly to the convention used in (Saito et al., 2006).


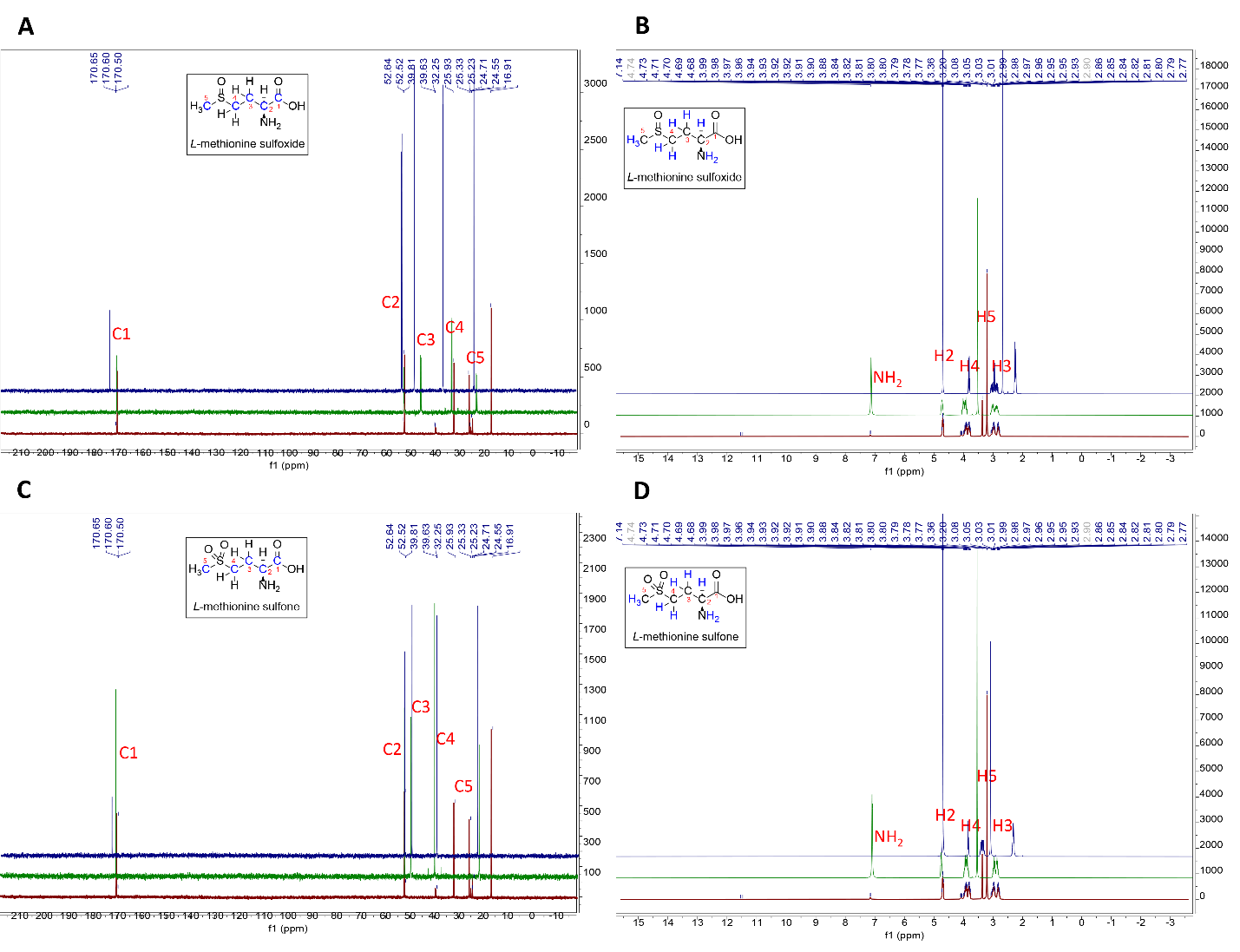
**Figure S30.** Comparison of the NMR spectra of methionine in concentrated sulfuric acid (98% D_2_SO_4_ and 2% D_2_O, by weight) to spectra of methionine sulfoxide or methionine sulfone. **A)** Comparison of the ^13^C NMR spectra of methionine, collected after four-week incubation in concentrated sulfuric acid (red spectra), to spectra of methionine sulfoxide in concentrated sulfuric acid (green spectra) and D_2_O (blue spectra). **B)** Comparison of the ^1^H NMR spectra of methionine, collected after four-week incubation (red spectra), to spectra of methionine sulfoxide in concentrated sulfuric acid (green spectra) and D_2_O (blue spectra). The solvent signal in ^1^H NMR spectra is suppressed for clarity. **C)** Comparison of the ^13^C NMR spectra of methionine, collected after four-week incubation (red spectra), to spectra of methionine sulfone in concentrated sulfuric acid (green spectra) and D_2_O (blue spectra). **D)** Comparison of the ^1^H NMR spectra of methionine, collected after four-week incubation (red spectra), to spectra of methionine sulfone in concentrated sulfuric acid (green spectra) and D_2_O (blue spectra). The solvent signal in ^1^H NMR spectra is suppressed for clarity. The spectra of methionine sulfoxide and methionine sulfone do not match the methionine spectra (red spectra), ruling out the direct oxidation of methionine in concentrated sulfuric acid. For consistency the C atoms on the illustrative structures of amino acids have been numbered accordingly to the convention used in (Saito et al., 2006).

**
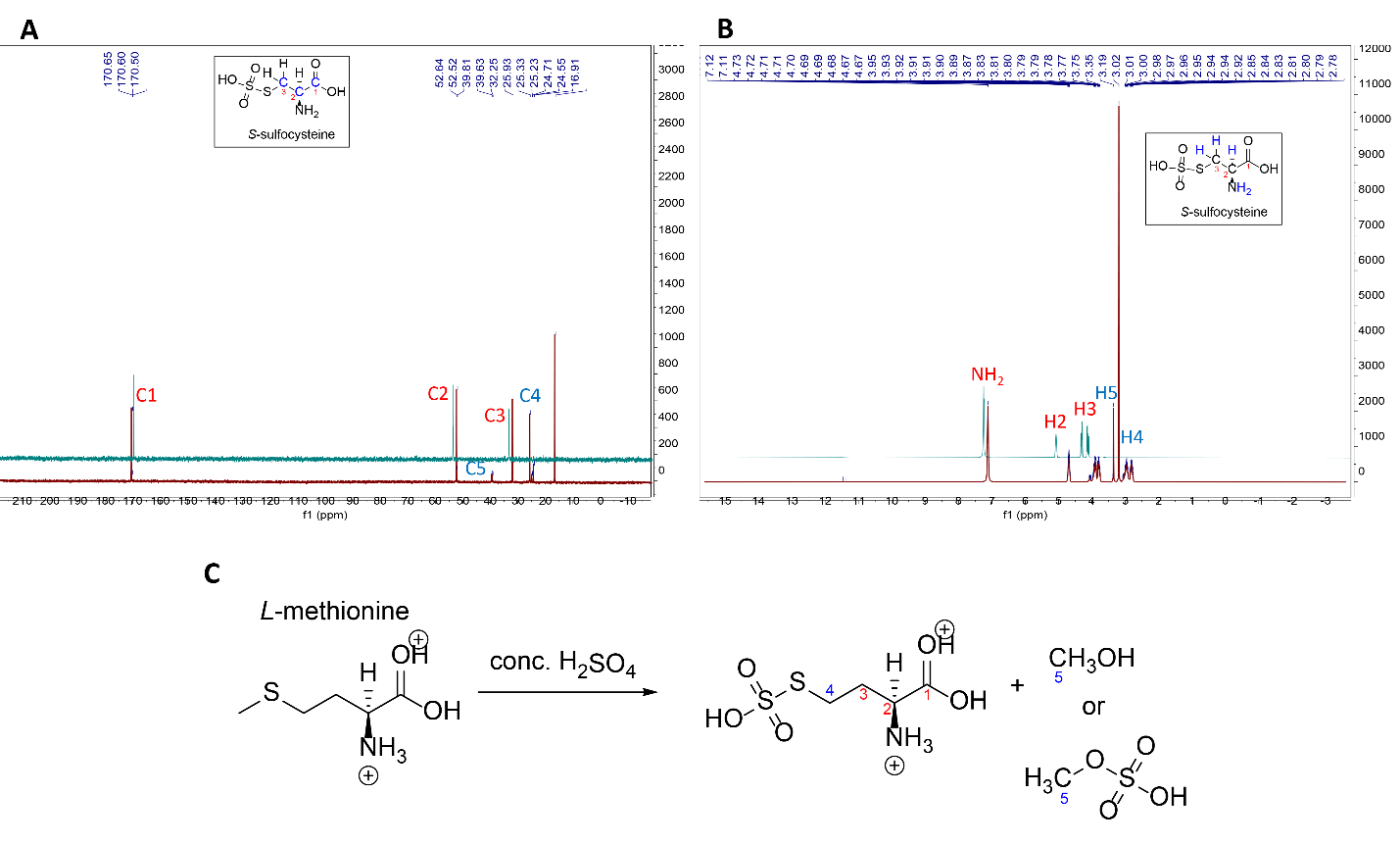
Figure S31.** Comparison of the NMR spectra of methionine in concentrated sulfuric acid (98% D_2_SO_4_ and 2% D_2_O, by weight) to spectra of *S*-sulfocysteine. **A)** Comparison of the ^13^C NMR spectra of methionine, collected after four-week incubation in concentrated sulfuric acid (red spectra), to spectra of *S*-sulfocysteine in concentrated sulfuric acid (teal spectra). **B)** Comparison of the ^1^H NMR spectra of methionine, collected after four-week incubation in concentrated sulfuric acid (red spectra), to spectra of *S*-sulfocysteine in concentrated sulfuric acid (teal spectra). The solvent signal in ^1^H NMR spectra is suppressed for clarity. **C)** Proposed reaction of methionine in concentrated sulfuric acid that yields *S*-sulfated homocysteine and methanol or sulfated methanol. The *S*-sulfocysteine spectra (teal) are similar to methionine spectra in concentrated sulfuric acid, supporting the hypothesis of *S*-sulfation of homocysteine in concentrated sulfuric acid. For consistency the C atoms on the illustrative structures of amino acids have been numbered accordingly to the convention used in (Saito et al., 2006).

**
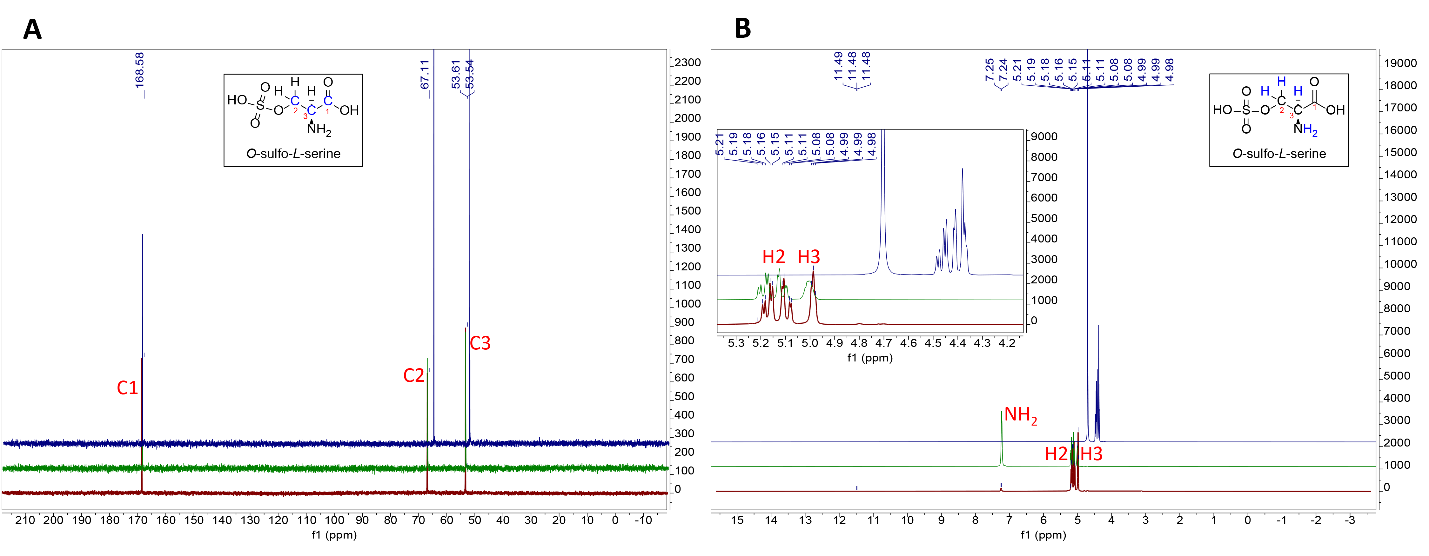
Figure S32.** Comparison of the ^13^C (**A**) and ^1^H (**B**) NMR spectra of serine, collected after four-week incubation (red spectra), to spectra of *O*-sulfoserine in concentrated sulfuric acid (98% D_2_SO_4_ and 2% D_2_O, by weight) (green spectra) and D_2_O (blue spectra), at room temperature. The solvent signal in ^1^H NMR spectra is suppressed for clarity. The *O*-sulfoserine spectra match the serine spectra (red spectra) confirming the sulfation of serine in concentrated sulfuric acid. For consistency the C atoms on the illustrative structures of amino acids have been numbered accordingly to the convention used in (Saito et al., 2006).

**
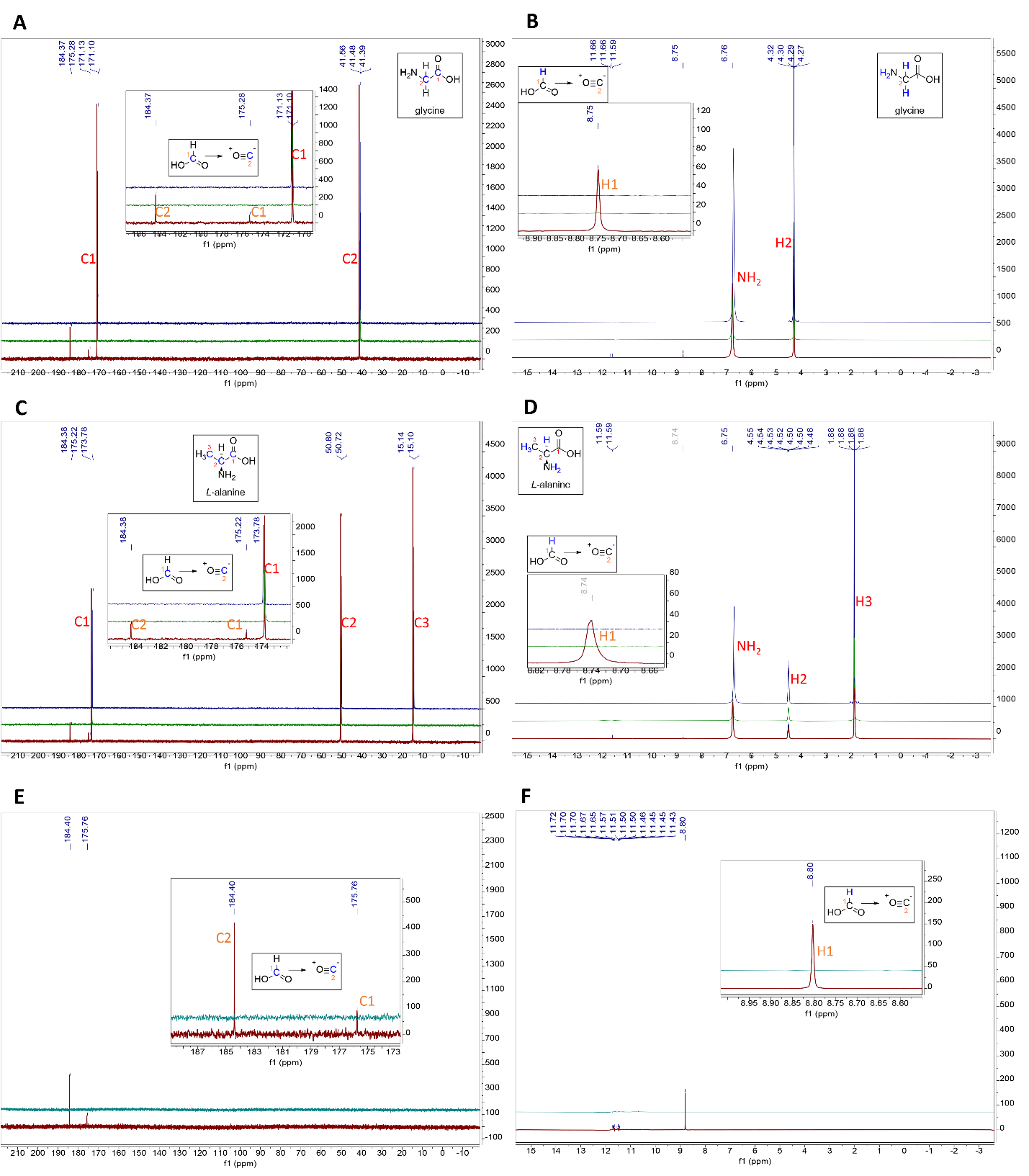
Figure S33.** Stability of glycine and alanine in 98% w/w sulfuric acid in the presence of carbon monoxide, at room temperature. **A)** Comparison of the ^13^C NMR spectra of glycine mixed in 1:1 molar ratio with formic acid (H_2_CO_2_; HCOOH), collected after 12h (red spectra) and 7-day (green spectra) incubation in concentrated sulfuric acid, to spectra of pure glycine in concentrated sulfuric acid (blue spectra). **B)** Comparison of the ^1^H NMR spectra of glycine mixed in 1:1 molar ratio with formic acid (H_2_CO_2_; HCOOH), collected after 12h (red spectra) and 7-day (green spectra) incubation in concentrated sulfuric acid, to spectra of pure glycine in concentrated sulfuric acid (blue spectra). The solvent signal in ^1^H NMR spectra is suppressed for clarity. **C)** Comparison of the ^13^C NMR spectra of alanine mixed in 1:1 molar ratio with formic acid (H_2_CO_2_; HCOOH), collected after 12h (red spectra) and 7-day (green spectra) incubation in concentrated sulfuric acid, to spectra of pure alanine in concentrated sulfuric acid (blue spectra). **D)** Comparison of the ^1^H NMR spectra of alanine mixed in 1:1 molar ratio with formic acid (H_2_CO_2_; HCOOH), collected after 12h (red spectra) and 7-day (green spectra) incubation in concentrated sulfuric acid, to spectra of pure alanine in concentrated sulfuric acid (blue spectra). The solvent signal in ^1^H NMR spectra is suppressed for clarity. **E)** Comparison of the ^13^C NMR spectra of formic acid (H_2_CO_2_; HCOOH) collected after 12h (red spectra) and 7-day (teal spectra) incubation in concentrated sulfuric acid. **F)** Comparison of the ^1^H NMR spectra of formic acid (H_2_CO_2_; HCOOH) collected after 12h (red spectra) and 7-day (teal spectra) incubation in concentrated sulfuric acid. The solvent signal in ^1^H NMR spectra is suppressed for clarity. Tested amino acids are stable for at least a week in concentrated sulfuric acid mixed with formic acid at room temperature. For consistency the C atoms on the illustrative structures of amino acids have been numbered accordingly to the convention used in (Saito et al., 2006).

**
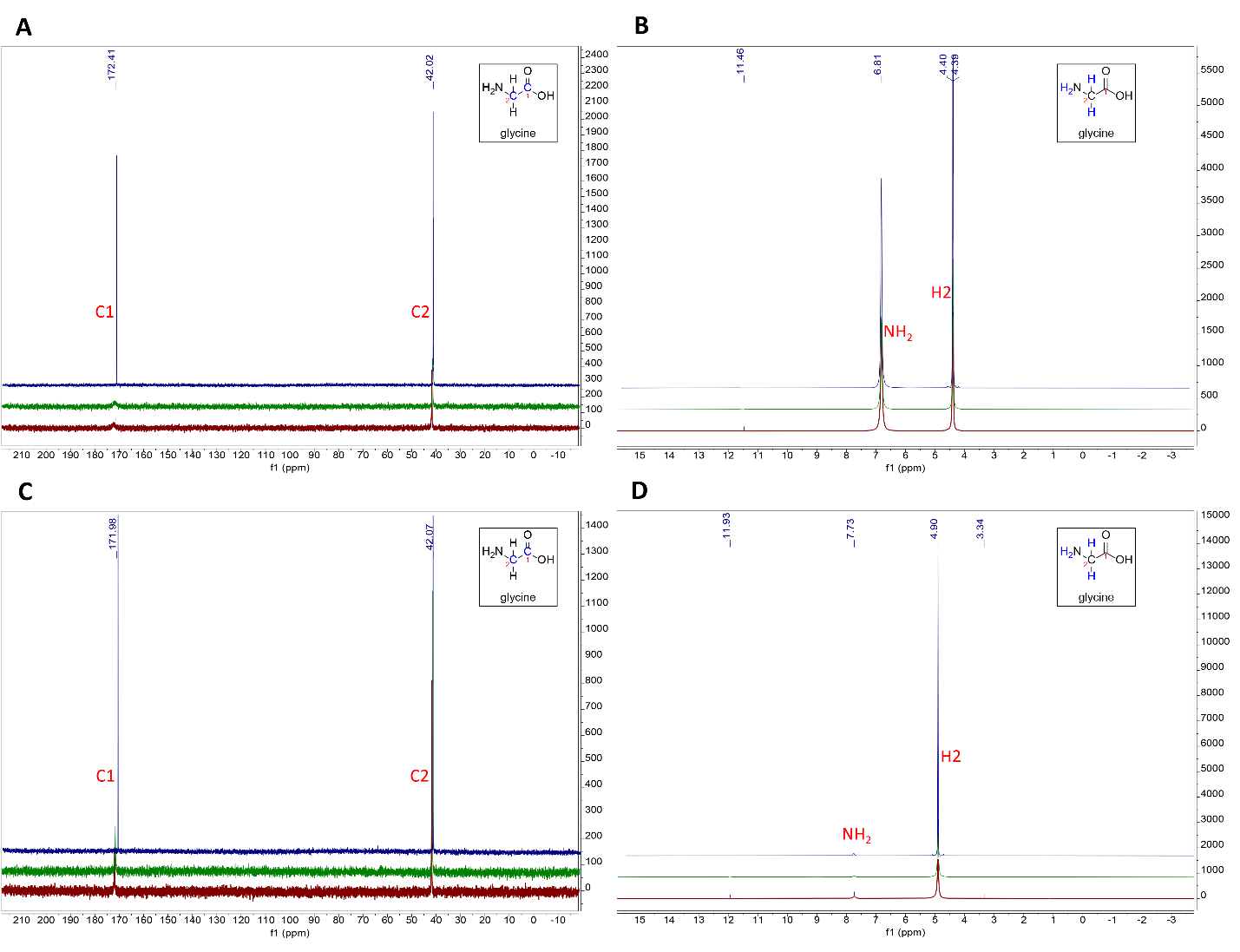
Figure S34.** Stability of glycine in 98% w/w and 81% w/w sulfuric acid in the presence of iron(II)oxide (FeO), at room temperature. **A)** Comparison of the ^13^C NMR spectra of glycine mixed in 1:1 molar ratio with FeO, collected after 12h (red spectra) and 7-day (green spectra) incubation in 98% w/w concentrated sulfuric acid, to spectra of pure glycine in 98% w/w concentrated sulfuric acid (blue spectra). **B)** Comparison of the ^1^H NMR spectra of glycine mixed in 1:1 molar ratio with FeO, collected after 12h (red spectra) and 7-day (green spectra) incubation in 98% w/w concentrated sulfuric acid, to spectra of pure glycine in 98% w/w concentrated sulfuric acid (blue spectra). The solvent signal in ^1^H NMR spectra is suppressed for clarity. **C)** Comparison of the ^13^C NMR spectra of glycine mixed in 1:1 molar ratio with FeO, collected after 12h (red spectra) and 7-day (green spectra) incubation in 81% w/w concentrated sulfuric acid, to spectra of pure glycine in 81% w/w concentrated sulfuric acid (blue spectra). **D)** Comparison of the ^1^H NMR spectra of glycine mixed in 1:1 molar ratio with FeO, collected after 12h (red spectra) and 7-day (green spectra) incubation in 81% w/w concentrated sulfuric acid, to spectra of pure glycine in 81% w/w concentrated sulfuric acid (blue spectra). The solvent signal in ^1^H NMR spectra is suppressed for clarity. Glycine is stable for at least a week in concentrated sulfuric acid mixed with FeO at room temperature. For consistency the C atoms on the illustrative structures of amino acids have been numbered accordingly to the convention used in (Saito et al., 2006).

**
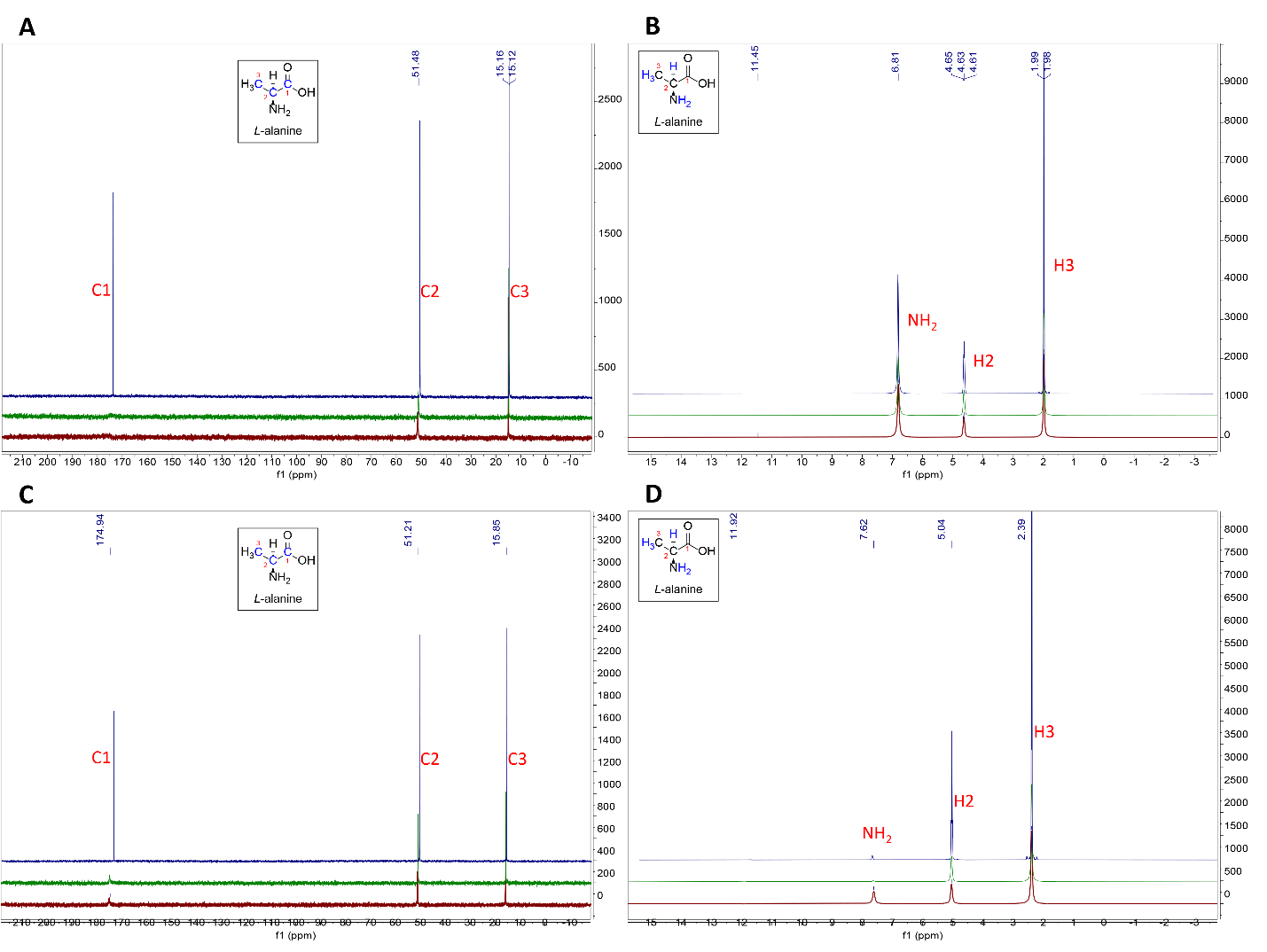
Figure S35.** Stability of alanine in 98% w/w and 81% w/w sulfuric acid in the presence of iron(II)oxide (FeO), at room temperature. **A)** Comparison of the ^13^C NMR spectra of alanine mixed in 1:1 molar ratio with FeO, collected after 12h (red spectra) and 7-day (green spectra) incubation in 98% w/w concentrated sulfuric acid, to spectra of pure alanine in 98% w/w concentrated sulfuric acid (blue spectra). **B)** Comparison of the ^1^H NMR spectra of alanine mixed in 1:1 molar ratio with FeO, collected after 12h (red spectra) and 7-day (green spectra) incubation in 98% w/w concentrated sulfuric acid, to spectra of pure alanine in 98% w/w concentrated sulfuric acid (blue spectra). The solvent signal in ^1^H NMR spectra is suppressed for clarity. **C)** Comparison of the ^13^C NMR spectra of alanine mixed in 1:1 molar ratio with FeO, collected after 12h (red spectra) and 7-day (green spectra) incubation in 81% w/w concentrated sulfuric acid, to spectra of pure alanine in 81% w/w concentrated sulfuric acid (blue spectra). **D)** Comparison of the ^1^H NMR spectra of alanine mixed in 1:1 molar ratio with FeO, collected after 12h (red spectra) and 7-day (green spectra) incubation in 81% w/w concentrated sulfuric acid, to spectra of pure alanine in 81% w/w concentrated sulfuric acid (blue spectra). The solvent signal in ^1^H NMR spectra is suppressed for clarity. Alanine is stable for at least a week in concentrated sulfuric acid mixed with FeO at room temperature. For consistency the C atoms on the illustrative structures of amino acids have been numbered accordingly to the convention used in (Saito et al., 2006).

| ***L*-alanine** | | | |
| --- | --- | --- | --- |
| **Solvent (reference)** | **C1 (ppm)** | **C2 (ppm)** | **C3 (ppm)** |
| D_2_O pH=1.20 (Saito et al., 2006) | 173.83 | 50.01 | 16.42 |
| D_2_SO_4_ 81% (w/w) | 173.54 | 50.86 | 16.08 |
| D_2_SO_4_ 98% (w/w) | 173.96 | 50.95 | 15.17 |

**Table S1.** Comparison of NMR chemical shifts of the tested amino acid obtained after 12-18h incubation in concentrated D_2_SO_4_ to values in acidic D_2_O reported in the literature. For consistency the C atoms have been numbered accordingly to the convention used in (Saito et al., 2006).

| ***L*-arginine** | | | | | | |
| --- | --- | --- | --- | --- | --- | --- |
| **Solvent (reference)** | **C1 (ppm)** | **C2 (ppm)** | **C3 (ppm)** | **C4 (ppm)** | **C5 (ppm)** | **C6 (ppm)** |
| D_2_O pH=1.33 (Saito et al., 2006) | 172.84 | 157.80 | 53.88 | 41.45 | 28.01 | 24.87 |
| D_2_SO_4_ 81% (w/w) | 172.37 | 157.16 | 54.38 | 41.58 | 27.68 | 24.72 |
| D_2_SO_4_ 98% (w/w) | 172.71 | 155.79 | 54.44 | 41.14 | 27.02 | 24.01 |

**Table S2.** Comparison of NMR chemical shifts of the tested amino acid obtained after 12-18h incubation in concentrated D_2_SO_4_ to values in acidic D_2_O reported in the literature. For consistency the C atoms have been numbered accordingly to the convention used in (Saito et al., 2006).

| ***L*-asparagine** | | | | |
| --- | --- | --- | --- | --- |
| **Solvent (reference)** | **C1 (ppm)** | **C2 (ppm)** | **C3 (ppm)** | **C4 (ppm)** |
| D_2_O pH=1.59 (Saito et al., 2006) | 174.46 | 172.26 | 50.85 | 34.92 |
| D_2_SO_4_ 81% (w/w) | 177.32 | 169.54 | 50.13 | 33.14 |
| D_2_SO_4_ 98% (w/w) | 176.53 | 169.28 | 49.59 | 31.97 |

**Table S3.** Comparison of NMR chemical shifts of the tested amino acid obtained after 12-18h incubation in concentrated D_2_SO_4_ to values in acidic D_2_O reported in the literature. For consistency the C atoms have been numbered accordingly to the convention used in (Saito et al., 2006).

| ***L*-aspartic acid** | | | | |
| --- | --- | --- | --- | --- |
| **Solvent (reference)** | **C1 (ppm)** | **C2 (ppm)** | **C3 (ppm)** | **C4 (ppm)** |
| D_2_O pH=2.85 (Saito et al., 2006) | 174.96 | 173.36 | 51.55 | 35.44 |
| D_2_SO_4_ 81% (w/w) | 175.55 | 170.76 | 50.76 | 33.76 |
| D_2_SO_4_ 98% (w/w) | 176.86 | 170.68 | 50.51 | 33.01 |

**Table S4.** Comparison of NMR chemical shifts of the tested amino acid obtained after 12-18h incubation in concentrated D_2_SO_4_ to values in acidic D_2_O reported in the literature. For consistency the C atoms have been numbered accordingly to the convention used in (Saito et al., 2006).

| ***L*-cysteine** | | | |
| --- | --- | --- | --- |
| **Solvent (reference)** | **C1 (ppm)** | **C2 (ppm)** | **C3 (ppm)** |
| D_2_O pH=1.75 (Saito et al., 2006) | 171.85 | 55.92 | 25.14 |
| D_2_SO_4_ 81% (w/w) | 170.80 | 53.33 | 36.10 |
| D_2_SO_4_ 98% (w/w) | 169.83 | 54.14 | 33.77 |

**Table S5.** Comparison of NMR chemical shifts of the tested amino acid obtained after 12-18h incubation in concentrated D_2_SO_4_ to values in acidic D_2_O reported in the literature. For consistency the C atoms have been numbered accordingly to the convention used in (Saito et al., 2006).

| **L-glutamine** | | | | | |
| --- | --- | --- | --- | --- | --- |
| **Solvent (reference)** | **C1 (ppm)** | **C2 (ppm)** | **C3 (ppm)** | **C4 (ppm)** | **C5 (ppm)** |
| D_2_O pH=1.37 (Saito et al., 2006) | 178.09 | 172.72 | 53.61 | 31.64 | 26.60 |
| D_2_SO_4_ 81% (w/w) | 181.29 | 171.09 | 53.31 | 29.78 | 25.40 |
| D_2_SO_4_ 98% (w/w) | 180.53 | 171.28 | 53.17 | 28.91 | 24.42 |

**Table S6.** Comparison of NMR chemical shifts of the tested amino acid obtained after 12-18h incubation in concentrated D_2_SO_4_ to values in acidic D_2_O reported in the literature. For consistency the C atoms have been numbered accordingly to the convention used in (Saito et al., 2006).

| **L-glutamic acid** | | | | | |
| --- | --- | --- | --- | --- | --- |
| **Solvent (reference)** | **C1 (ppm)** | **C2 (ppm)** | **C3 (ppm)** | **C4 (ppm)** | **C5 (ppm)** |
| D_2_O pH=3.34 (Saito et al., 2006) | 177.83 | 174.45 | 54.78 | 30.94 | 26.39 |
| D_2_SO_4_ 81% (w/w) | 180.57 | 171.89 | 53.69 | 30.61 | 25.04 |
| D_2_SO_4_ 98% (w/w) | 188.91 | 171.49 | 52.94 | 29.89 | 23.45 |

**Table S7.** Comparison of NMR chemical shifts of the tested amino acid obtained after 12-18h incubation in concentrated D_2_SO_4_ to values in acidic D_2_O reported in the literature. For consistency the C atoms have been numbered accordingly to the convention used in (Saito et al., 2006).

| **Glycine** | | |
| --- | --- | --- |
| **Solvent (reference)** | **C1 (ppm)** | **C2 (ppm)** |
| D_2_O pH=1.28 (Saito et al., 2006) | 171.02 | 41.36 |
| D_2_SO_4_ 81% (w/w) | 170.98 | 41.92 |
| D_2_SO_4_ 98% (w/w) | 171.27 | 41.66 |

**Table S8.** Comparison of NMR chemical shifts of the tested amino acid obtained after 12-18h incubation in concentrated D_2_SO_4_ to values in acidic D_2_O reported in the literature. For consistency the C atoms have been numbered accordingly to the convention used in (Saito et al., 2006).

| ***L*-histidine** | | | | | | |
| --- | --- | --- | --- | --- | --- | --- |
| **Solvent (reference)** | **C1 (ppm)** | **C2 (ppm)** | **C3 (ppm)** | **C4 (ppm)** | **C5 (ppm)** | **C6 (ppm)** |
| D_2_O pH=1.74 (Saito et al., 2006) | 172.09 | 134.98 | 127.67 | 118.89 | 53.59 | 26.20 |
| D_2_SO_4_ 81% (w/w) | 170.44 | 135.60 | 126.72 | 120.03 | 53.42 | 25.38 |
| D_2_SO_4_ 98% (w/w) | 170.58 | 134.90 | 125.69 | 119.65 | 53.33 | 24.58 |

**Table S9.** Comparison of NMR chemical shifts of the tested amino acid obtained after 12-18h incubation in concentrated D_2_SO_4_ to values in acidic D_2_O reported in the literature. For consistency the C atoms have been numbered accordingly to the convention used in (Saito et al., 2006).

| ***L*-isoleucine** | | | | | | |
| --- | --- | --- | --- | --- | --- | --- |
| **Solvent (reference)** | **C1 (ppm)** | **C2 (ppm)** | **C3 (ppm)** | **C4 (ppm)** | **C5 (ppm)** | **C6 (ppm)** |
| D_2_O (Saito et al., 2006) | 175.04 | 60.51 | 36.80 | 25.38 | 15.61 | 12.02 |
| D_2_SO_4_ 81% (w/w) | 172.34 | 59.07 | 36.82 | 25.58 | 14.67 | 11.83 |
| D_2_SO_4_ 98% (w/w) | 172.95 | 59.18 | 36.51 | 24.83 | 13.84 | 10.88 |

**Table S10.** Comparison of NMR chemical shifts of the tested amino acid obtained after 12-18h incubation in concentrated D_2_SO_4_ to values in acidic D_2_O reported in the literature. For consistency the C atoms have been numbered accordingly to the convention used in (Saito et al., 2006).

| ***L*-leucine** | | | | | |
| --- | --- | --- | --- | --- | --- |
| **Solvent (reference)** | **C1 (ppm)** | **C2 (ppm)** | **C3 (ppm)** | **C4 (ppm)** | **C5 (ppm)** |
| D_2_O pH=1.15 (Saito et al., 2006) | 173.76 | 52.74 | 40.01 | 25.06 | 22.70, 22.13 |
| D_2_SO_4_ 81% (w/w) | 173.46 | 53.36 | 39.32 | 25.00 | 22.37, 21.76 |
| D_2_SO_4_ 98% (w/w) | 174.03 | 53.47 | 38.83 | 24.43 | 21.52, 20.85 |

**Table S11.** Comparison of NMR chemical shifts of the tested amino acid obtained after 12-18h incubation in concentrated D_2_SO_4_ to values in acidic D_2_O reported in the literature. For consistency the C atoms have been numbered accordingly to the convention used in (Saito et al., 2006).

| ***L*-lysine** | | | | | | |
| --- | --- | --- | --- | --- | --- | --- |
| **Solvent (reference)** | **C1 (ppm)** | **C2 (ppm)** | **C3 (ppm)** | **C4 (ppm)** | **C5 (ppm)** | **C6 (ppm)** |
| D_2_O pH=1.68 (Saito et al., 2006) | 173.23 | 54.02 | 40.10 | 30.21 | 27.15 | 22.27 |
| D_2_SO_4_ 81% (w/w) | 172.52 | 54.49 | 41.65 | 29.81 | 27.10 | 22.16 |
| D_2_SO_4_ 98% (w/w) | 172.84 | 54.51 | 41.38 | 29.18 | 26.29 | 21.43 |

**Table S12.** Comparison of NMR chemical shifts of the tested amino acid obtained after 12-18h incubation in concentrated D_2_SO_4_ to values in acidic D_2_O reported in the literature. For consistency the C atoms have been numbered accordingly to the convention used in (Saito et al., 2006).

| ***L*-Methionine** | | | | | |
| --- | --- | --- | --- | --- | --- |
| **Solvent (reference)** | **C1 (ppm)** | **C2 (ppm)** | **C3 (ppm)** | **C4 (ppm)** | **C5 (ppm)** |
| D_2_O pH=1.55 (Saito et al., 2006) | 172.90 | 53.15 | 29.94 | 29.48 | 14.82 |
| D_2_SO_4_ 81% (w/w) | 172.08 | 53.82 | 29.94 | 28.58 | 15.14 |
| D_2_SO_4_ 98% (w/w) | see text | see text | see text | see text | see text |

**Table S13.** Comparison of NMR chemical shifts of the tested amino acid obtained after 12-18h incubation in concentrated D_2_SO_4_ to values in acidic D_2_O reported in the literature. For consistency the C atoms have been numbered accordingly to the convention used in (Saito et al., 2006).

| ***L*-Phenylalanine** | | | | | | | |
| --- | --- | --- | --- | --- | --- | --- | --- |
| **Solvent (reference)** | **C1 (ppm)** | **C2 (ppm)** | **C3 (ppm)** | **C4 (ppm)** | **C5 (ppm)** | **C6 (ppm)** | **C7 (ppm)** |
| D_2_O (Saito et al., 2006) | 174.24 | 136.30 | 130.19 | 129.91 | 128.42 | 56.94 | 37.21 |
| D_2_SO_4_ 81% (w/w) | 172.07 | 132.94 | 130.58 | 130.17 | 129.55 | 55.67 | 36.03 |
| D_2_SO_4_ 98% (w/w) | see text | see text | see text | see text | see text | see text | see text |

**Table S14.** Comparison of NMR chemical shifts of the tested amino acid obtained after 12-18h incubation in concentrated D_2_SO_4_ to values in acidic D_2_O reported in the literature. For consistency the C atoms have been numbered accordingly to the convention used in (Saito et al., 2006).

| ***L*-proline** | | | | | |
| --- | --- | --- | --- | --- | --- |
| **Solvent (reference)** | **C1 (ppm)** | **C2 (ppm)** | **C3 (ppm)** | **C4 (ppm)** | **C5 (ppm)** |
| D_2_O pH=1.27 (Saito et al., 2006) | 173.30 | 61.04 | 47.35 | 29.42 | 24.44 |
| D_2_SO_4_ 81% (w/w) | 173.11 | 61.09 | 49.23 | 29.40 | 24.63 |
| D_2_SO_4_ 98% (w/w) | 173.72 | 60.64 | 49.10 | 28.83 | 23.94 |

**Table S15.** Comparison of NMR chemical shifts of the tested amino acid obtained after 12-18h incubation in concentrated D_2_SO_4_ to values in acidic D_2_O reported in the literature. For consistency the C atoms have been numbered accordingly to the convention used in (Saito et al., 2006).

| ***L*-serine** | | | |
| --- | --- | --- | --- |
| **Solvent (reference)** | **C1 (ppm)** | **C2 (ppm)** | **C3 (ppm)** |
| D_2_O pH=1.12 (Saito et al., 2006) | 171.26 | 60.44 | 55.97 |
| D_2_SO_4_ 81% (w/w) | see text | see text | see text |
| D_2_SO_4_ 98% (w/w) | 168.57 | 67.13 | 53.56 |

**Table S16.** Comparison of NMR chemical shifts of the tested amino acid obtained after 12-18h incubation in concentrated D_2_SO_4_ to values in acidic D_2_O reported in the literature. For consistency the C atoms have been numbered accordingly to the convention used in (Saito et al., 2006).

| ***L*-threonine** | | | | |
| --- | --- | --- | --- | --- |
| **Solvent (reference)** | **C1 (ppm)** | **C2 (ppm)** | **C3 (ppm)** | **C4 (ppm)** |
| D_2_O pH=1.36 (Saito et al., 2006) | 171.67 | 66.34 | 59.76 | 20.15 |
| D_2_SO_4_ 81% (w/w) | see text | see text | see text | see text |
| D_2_SO_4_ 98% (w/w) | 169.04 | 77.52 | 58.16 | 16.98 |

**Table S17.** Comparison of NMR chemical shifts of the tested amino acid obtained after 12-18h incubation in concentrated D_2_SO_4_ to values in acidic D_2_O reported in the literature. For consistency the C atoms have been numbered accordingly to the convention used in (Saito et al., 2006).

| ***L*-Tryptophan** | | | | | | | | | | |
| --- | --- | --- | --- | --- | --- | --- | --- | --- | --- | --- |
| **Solvent (reference)** | **C1 (ppm)** | **C2 (ppm)** | **C3 (ppm)** | **C4 (ppm)** | **C5 (ppm)** | **C6 (ppm)** | **C7 (ppm)** | **C8 (ppm)** | **C9 (ppm)** | **C10 (ppm)** |
| D_2_O (Saito et al., 2006) | 174.74 | 137.28 | 127.69 | 125.76 | 122.93 | 120.27 | 119.27 | 112.74 | 108.74 | 56.10 |
| D_2_SO_4_ 81% (w/w) | see text | see text | see text | see text | see text | see  text | see  text | see text | see text | see  text |
| D_2_SO_4_ 98% (w/w) | see text | see text | see text | see text | see text | see  text | see  text | see text | see text | see  text |

**Table S18.** Comparison of NMR chemical shifts of the tested amino acid obtained after 12-18h incubation in concentrated D_2_SO_4_ to values in acidic D_2_O reported in the literature. For consistency the C atoms have been numbered accordingly to the convention used in (Saito et al., 2006).

| ***L*-Tyrosine** | | | | | | | |
| --- | --- | --- | --- | --- | --- | --- | --- |
| **Solvent (reference)** | **C1 (ppm)** | **C2 (ppm)** | **C3 (ppm)** | **C4 (ppm)** | **C5 (ppm)** | **C6 (ppm)** | **C7 (ppm)** |
| D_2_O acidic (Li et al., 2020) | 171.13 | 155.00 | 130.76 | 125.40 | 115.84 | 53.95 | 34.54 |
| D_2_SO_4_ 81% (w/w) | see text | see text | see text | see text | see text | see text | see text |
| D_2_SO_4_ 98% (w/w) | see text | see text | see text | see text | see text | see text | see text |

**Table S19.** Comparison of NMR chemical shifts of the tested amino acid obtained after 12-18h incubation in concentrated D_2_SO_4_ to values in acidic D_2_O reported in the literature. For consistency the C atoms have been numbered accordingly to the convention used in (Saito et al., 2006).

| ***L*-valine** | | | | |
| --- | --- | --- | --- | --- |
| **Solvent (reference)** | **C1 (ppm)** | **C2 (ppm)** | **C3 (ppm)** | **C4 (ppm)** |
| D_2_O pH=1.34 (Saito et al., 2006) | 172.70 | 59.60 | 30.04 | 18.45, 17.93 |
| D_2_SO_4_ 81% (w/w) | 172.41 | 60.02 | 30.15 | 17.96, 17.65 |
| D_2_SO_4_ 98% (w/w) | 172.98 | 60.08 | 29.77 | 17.10, 16.62 |

**Table S20.** Comparison of NMR chemical shifts of the tested amino acid obtained after 12-18h incubation in concentrated D_2_SO_4_ to values in acidic D_2_O reported in the literature. For consistency the C atoms have been numbered accordingly to the convention used in (Saito et al., 2006).

**Supplementary Datasets:**

*Supplementary Dataset S1:* The original 1D ^1^H and ^13^C NMR data collected in 81% w/w and 98% w/w concentrated sulfuric acid, at all three time intervals, for all 20 amino acids. Supplementary Dataset S1 also contains original 1D ^1^H and ^13^C NMR data for modified amino acids such as *S*-sulfocysteine, *O*-sulfoserine, oxidized cysteine, oxidized methionine as well as original 1D ^1^H and ^13^C NMR data for tests with formic acid and FeO. All data can be downloaded from Zenodo at https://zenodo.org/record/8381013.

*Supplementary Dataset S2:* The original 2D ^1^H-^13^C HMQC NMR data for all 20 amino acids collected in 98% w/w concentrated sulfuric acid can be downloaded from Zenodo at <https://zenodo.org/record/8381013>.

**Supplementary References:**

Aguiar RM, Leão RAC, Mata A, et al. Continuous-Flow Protocol for the Synthesis of Enantiomerically Pure Intermediates of Anti Epilepsy and Anti Tuberculosis Active Pharmaceutical Ingredients. Org Biomol Chem 2019;17(6):1552–1557.

Andrews JC and Bruce RB. The Reactions of the Sulfur-Containing Amino Acids with Phosphoric and Sulfuric Acids. Arch Biochem Biophys 1951;33(3):427–435.

Burzio LA and Waite JH. The Other Topa: Formation of 3, 4, 5-Trihydroxyphenylalanine in Peptides. Anal Biochem 2002;306(1):108–114.

Butz LW and du Vigneaud V. The Formation of a Homologue of Cystine by the Decomposition of Methionine with Sulfuric Acid. J Biol Chem 1932;99(1):135–142.

Elsherbini M, Allemann RK and Wirth T. “Dark” Singlet Oxygen Made Easy. Chem Eur J 2019;25(54):12486–12490.

Emmanuel N, Mendoza C, Winter M, et al. Scalable Photocatalytic Oxidation of Methionine under Continuous-Flow Conditions. Org Process Res Dev 2017;21(9):1435–1438.

Fegley B. Why Pyrite Is Unstable on the Surface of Venus. Icarus 1997;128(2):474–479; doi: https://doi.org/10.1006/icar.1997.5744.

Garrido Ruiz D, Sandoval-Perez A, Rangarajan AV, et al. Cysteine Oxidation in Proteins: Structure, Biophysics, and Simulation. Biochemistry 2022;61(20):2165–2176.

Habeeb A. The Reaction of Sulphuric Acid with Lysozyme and Horse Globin. Can J Biochem Physiol 1961;39(1):31–43.

Hu W, Song H, Sae Her A, et al. Bioinformatic and Biochemical Characterizations of C–S Bond Formation and Cleavage Enzymes in the Fungus Neurospora Crassa Ergothioneine Biosynthetic Pathway. Org Lett 2014;16(20):5382–5385.

Iraci LT, Essin AM and Golden DM. Solubility of Methanol in Low-Temperature Aqueous Sulfuric Acid and Implications for Atmospheric Particle Composition. J Phys Chem A 2002;106(16):4054–4060.

Jeon M, Halbert M V, Stephen ZR, et al. Iron Oxide Nanoparticles as T1 Contrast Agents for Magnetic Resonance Imaging: Fundamentals, Challenges, Applications, and Prospectives. Adv Mater 2021;33(23):1906539.

Kane SM and Leu M-T. Uptake of Methanol Vapor in Sulfuric Acid Solutions. J Phys Chem A 2001;105(9):1411–1415.

Karunakaran-Datt A and Kennepohl P. Redox Photochemistry of Methionine by Sulfur K-Edge X-Ray Absorption Spectroscopy: Potential Implications for Cataract Formation. J Am Chem Soc 2009;131(10):3577–3582.

Kim G, Weiss SJ and Levine RL. Methionine Oxidation and Reduction in Proteins. Biochim Biophys Acta (BBA)-General Subj 2014;1840(2):901–905.

Koch H and Haaf W. Über Die Synthese Verzweigter Carbonsäuren Nach Der Ameisensäure‐Methode. Justus Liebigs Ann Chem 1958;618(1):251–266.

Li G, Lian J, Xue H, et al. Biocascade Synthesis of L‐Tyrosine Derivatives by Coupling a Thermophilic Tyrosine Phenol‐Lyase and L‐Lactate Oxidase. European J Org Chem 2020;2020(8):1050–1054.

Liang X, Kaya A, Zhang Y, et al. Characterization of Methionine Oxidation and Methionine Sulfoxide Reduction Using Methionine-Rich Cysteine-Free Proteins. BMC Biochem 2012;13(1):1–10.

Marcq E, Mills FP, Parkinson CD, et al. Composition and Chemistry of the Neutral Atmosphere of Venus. Space Sci Rev 2018;214(1):10.

Mogul R, Limaye SS, Way MJ, et al. Venus’ Mass Spectra Show Signs of Disequilibria in the Middle Clouds. Geophys Res Lett 2021;e2020GL091327.

Pace AL, Wong RL, Zhang YT, et al. Asparagine Deamidation Dependence on Buffer Type, PH, and Temperature. J Pharm Sci 2013;102(6):1712–1723.

Ponder GR and Richards GN. Pyrolysis of Some 13C-Labeled Glucans: A Mechanistic Study. Carbohydr Res 1993;244(1):27–47.

Popper KR. Pyrite and the Origin of Life. Nature 1990;344(6265):387.

Ramachandran LK and McConnell WB. The Action of Sulphuric Acid on Gliadin: With Special Reference to the N-Peptidyl→ O-Peptidyl Bond Rearrangement. Can J Chem 1955;33(11):1638–1648.

Recky JRN, Serrano MP, Dántola ML, et al. Oxidation of Tyrosine: Antioxidant Mechanism of l-DOPA Disclosed. Free Radic Biol Med 2021;165:360–367.

Reitz HC, Ferrel RE, Fraenkel-Conrat H, et al. Action of Sulfating Agents on Proteins and Model Substances. I. Concentrated Sulfuric Acid. J Am Chem Soc 1946;68(6):1024–1031.

Riggs DL, Silzel JW, Lyon YA, et al. Analysis of Glutamine Deamidation: Products, Pathways, and Kinetics. Anal Chem 2019;91(20):13032–13038.

Rose JE, Leeson PD and Gani D. Mechanisms and Stereochemistry of the Activation of (2 S)-and (2 R)-Serine O-Sulfate as Suicide Inhibitors for Escherichia Coli Glutamic Acid Decarboxylase. J Chem Soc Perkin Trans 1 1994;(21):3089–3094.

Saito T, Hayamizu K, Yanagisawa M, et al. Spectral Database for Organic Compounds (Sdbs). Natl Inst Adv Ind Sci Technol 2006.

Sen KI, Hepler R and Nanda H. Detection and Measurement of Methionine Oxidation in Proteins. Curr Protoc Protein Sci 2017;87(1):14–16.

Skvortsov AN, Zavodnik VE, Stash AI, et al. Molecular Structure and Spectral Properties of Methionine Sulfone, Product of Methionine Oxidation. Russ J Org Chem 2003;39:170–175.

Stanulis A and Barron AR. Process for Converting Disulfides to Conversion Products and Process for Producing Cysteic Acid. 2022.

Stoebenau EJ and Jordan RF. Coordination of Alkenes and Alkynes to a Cationic D0 Zirconocene Alkoxide Complex. J Am Chem Soc 2003;125(11):3222–3223.

Surkov YA, Barsukov VL, Moskalyeva LP, et al. New Data on the Composition, Structure, and Properties of Venus Rock Obtained by Venera 13 and Venera 14. J Geophys Res Solid Earth 1984;89(S02):B393–B402; doi: 10.1029/JB089iS02p0B393.

Surkov YA, Moskalyova LP, Kharyukova VP, et al. Venus Rock Composition at the Vega 2 Landing Site. J Geophys Res Solid Earth 1986;91(B13):E215–E218; doi: 10.1029/JB091iB13p0E215.

Weierbach SM, Reynolds RP, Stephens SM, et al. Chemoselective Oxidation of Thiols with Oxoammonium Cations. J Org Chem 2023;88(16):11392–11410; doi: 10.1021/acs.joc.2c01097.

Xu X, Yan L, Wang S, et al. Selective Synthesis of Sulfoxides and Sulfones via Controllable Oxidation of Sulfides with N-Fluorobenzenesulfonimide. Org Biomol Chem 2021;19(40):8691–8695.

Zolotov MY, Mogul R, Limaye SS, et al. Venus Cloud Composition Suggested From The Pioneer Venus Large Probe Neutral Mass Spectrometer Data. LPI Contrib 2023;2806:2880.

1. We note that cysteine, after ready conversion to *S*-sulfocysteine, appears to slowly undergo further modification. This change is illustrated by the emergence of new peaks on the ^13^C and ^1^H spectra after four-week incubation in 98% w/w sulfuric acid (Figure S8A, Figure S28). We do not know what this additional modification is, it does not however lead to the instability of the overall amino acid structure. [↑](#footnote-ref-1)
2. Note that pyrite is thermodynamically unstable on the surface of Venus, with the possible exception of the Venusian mountain tops (Fegley, 1997). [↑](#footnote-ref-2)
3. In aqueous acidic solutions (pH 1-3) the amino -NH_2_ groups and the carboxylic OH groups of amino acids will be protonated, just as they are in concentrated sulfuric acid, resulting in similar chemical shifts. For that reason we choose to compare known chemical shifts recorded in acidic aqueous solutions to chemical shifts recorded in concentrated sulfuric acid, rather than to comparisons to aqueous solutions of other pH ranges.  [↑](#footnote-ref-3)
4. In aqueous acidic solutions (pH 1-3) the amino -NH_2_ groups and the carboxylic OH groups of amino acids will be protonated, just as they are in concentrated sulfuric acid, resulting in similar chemical shifts. For that reason we choose to compare known chemical shifts recorded in acidic aqueous solutions to chemical shifts recorded in concentrated sulfuric acid, rather than to comparisons to aqueous solutions of other pH ranges.  [↑](#footnote-ref-4)
